# Phase II Metabolite-Inspired Tripeptide Editing Generates Anti-Inflammatory Candidates for Treatment-Refractory Ulcerative Colitis

**DOI:** 10.3390/ijms27188285

**Published:** 2026-09-17

**Authors:** Xiaodi Shi, Rabeya Jafrin Mow, Dingpei Long, Olivier Merlin-Zhang, Emma Xu, Xiaomeng Shi, Pallavi Garg, Shanthi Srinivasan, Chunhua Yang

**Affiliations:** 1 Digestive Disease Research Group, Institute for Biomedical Sciences, Georgia State University, Atlanta, GA 30303, USA; xshi10@student.gsu.edu (X.S.); rmow1@student.gsu.edu (R.J.M.); dlong26@gsu.edu (D.L.); xshi8@gsu.edu (X.S.); pgarg@gsu.edu (P.G.); 2 School of Medicine, Wake Forest University, Winston-Salem, NC 27101, USA; olivier.merlinzhang@wfusm.edu (O.M.-Z.); emma.xu@wfusm.edu (E.X.); 3 Division of Digestive Diseases, Emory University School of Medicine, Atlanta, GA 30307, USA; ssrini2@emory.edu; 4 Atlanta Veterans Affairs Medical Center, Decatur, GA 30302, USA

**Keywords:** inflammatory bowel disease, phenotypic drug screening, bio-inspired structure modification, ex vivo patient biopsy model

## Abstract

Ulcerative colitis remains a major clinical challenge due to limited availability of safe, orally active anti-inflammatory drugs. Although phase II metabolism is traditionally viewed as a detoxification process, certain metabolites retain or exhibit enhanced pharmacological activity, offering an underexplored drug discovery opportunity. Here, we investigated phase II metabolite-inspired tripeptide editing using M13, a glutathione-conjugated metabolite of the anti-inflammatory natural product 6-shogaol, as a lead scaffold. A focused library of 38 analogs was generated by systematic tripeptide editing while preserving the central cysteine-containing 6-shogaol-derived scaffold. Phenotypic nuclear factor-κB (NF-κB) reporter screening identified 29 of 38 analogs with greater inhibitory activity than M13 at the screening concentration. Two selected leads, MLY2 and MLY8, demonstrated enhanced anti-inflammatory activity in Dextran Sulfate Sodium-induced experimental colitis, including restoration of colon length and reductions in fecal lipocalin-2 and pro-inflammatory cytokines. Both compounds also suppressed inflammatory cytokine production more effectively than M13 in ex vivo colonic biopsies from patients with treatment-refractory ulcerative colitis. In parallel, MLY2 and MLY8 showed no detectable mutagenicity under the Ames assay conditions and were well tolerated in a preliminary single-dose maximum tolerated dose study. Collectively, these findings establish phase II metabolite-inspired tripeptide editing as a productive lead-discovery strategy and identify MLY2 and MLY8 as anti-inflammatory leads warranting further preclinical characterization.

## 1. Introduction

Ulcerative colitis (UC) is a chronic inflammatory disease of the gastrointestinal tract characterized by relapsing mucosal inflammation and progressive epithelial barrier damage [1,2]. Despite major advances in biologic and small-molecule therapies, a substantial proportion of patients fail to achieve sustained remission or experience secondary loss of response [3,4,5,6]. Treatment-refractory UC remains a major clinical challenge, with colectomy rates reaching approximately 10% at 10 years post-diagnosis despite advances in biologic therapy, highlighting the need for safer and more effective anti-inflammatory drug candidates [7].

Phase II metabolism is traditionally regarded as a detoxification process that facilitates drug inactivation and elimination [8]. However, emerging evidence suggests that certain phase II metabolites can retain or even exhibit enhanced biological activity relative to their parent compounds, providing an underexplored opportunity for anti-inflammatory lead discovery [9,10,11]. For example, morphine-6-glucuronide (M6G), a major phase II metabolite of morphine, displays potent analgesic activity distinct from the parent drug [12]. Similarly, we previously identified M13, a glutathione-conjugated metabolite of the anti-inflammatory natural product 6-shogaol [13,14,15,16,17], as a biologically active metabolite with enhanced anti-inflammatory activity relative to 6-shogaol [18].

Inspired by these observations, we hypothesized that biologically active phase II metabolites may represent chemically productive scaffolds for medicinal chemistry optimization. Using M13 as a lead structure, we explored systematic amino acid substitution within the glutathione-derived tripeptide moiety while retaining the central cysteine-containing 6-shogaol-derived scaffold. The electrophilic α,β-unsaturated carbonyl (Michael acceptor) was retained as a structural design feature because electrophilic compounds and 6-shogaol-derived metabolites have been associated with anti-inflammatory signaling in prior studies [19,20,21]. However, the present design does not assume that electrophilic reactivity is the experimentally established mechanism of the MLY analogs. A focused library of 38 analogs was generated by replacing the flanking amino acid residues of the glutathione tripeptide with naturally occurring amino acids selected to preserve related structural and polarity characteristics. To evaluate anti-inflammatory activity, we employed an NF-κB/293/GFP-Luc™ reporter system as a phenotypic screening platform centered on inflammatory pathway modulation [22].

Here, we investigated whether phase II metabolite-inspired tripeptide editing could generate anti-inflammatory leads with improved biological activity relative to the parent metabolite scaffold. The NF-κB assay was used as a primary activity screen, followed by concentration–response characterization of selected leads. MLY2 and MLY8 were subsequently evaluated in dextran sulfate sodium (DSS)-induced experimental colitis, ex vivo colonic biopsies from patients with treatment-refractory UC, and preliminary safety/tolerability studies.

## 2. Results

### 2.1. Phase II Metabolite-Inspired Tripeptide Editing Identifies Anti-Inflammatory Analogs

To explore whether phase II metabolites could serve as chemically productive scaffolds for drug discovery, we selected M13, a glutathione-conjugated metabolite of 6-shogaol, as a lead structure for systematic medicinal chemistry optimization. Previous studies demonstrated that M13 exhibits greater anti-inflammatory activity and improved biopharmaceutical properties compared with its parent compound, 6-shogaol, suggesting that metabolite scaffolds may provide unique opportunities for identifying biologically active analogs [18]. Building on this rationale, we investigated whether selective editing of the glutathione tripeptide sequence could improve anti-inflammatory activity while retaining the central cysteine-containing 6-shogaol-derived scaffold. The α,β-unsaturated carbonyl was preserved as a structural feature of the analog series; its direct contribution to the activity of MLY2 and MLY8 was not established in the present study [19,20,21,23].

**Figure 1:**
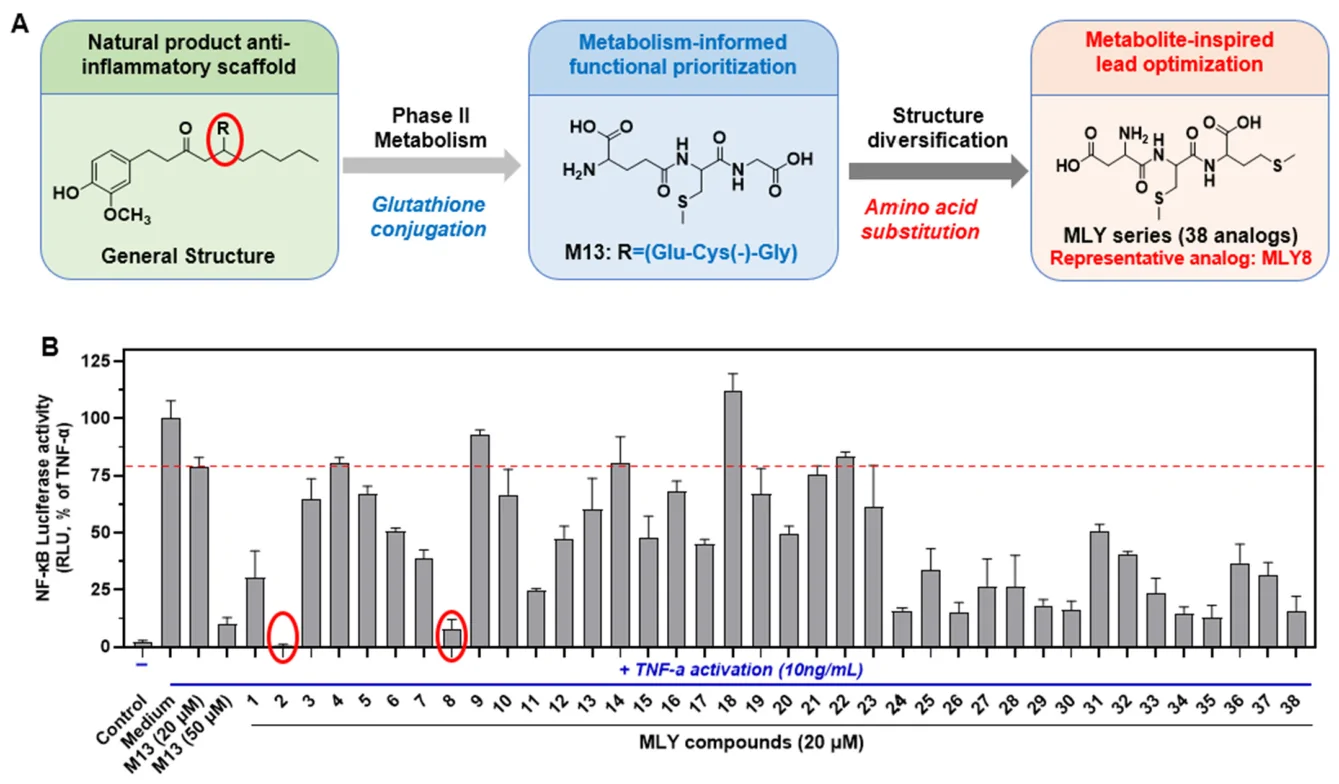
Phase II metabolite-inspired tripeptide editing identifies NF-κB inhibitory analogs. (**A**) Discovery strategy. Metabolic profiling of the anti-inflammatory natural product 6-shogaol identified M13, a glutathione-conjugated phase II metabolite with anti-inflammatory activity. Systematic tripeptide editing of the M13 scaffold generated a focused library of 38 MLY analogs (structures summarized in Table S1), which were evaluated by phenotypic anti-inflammatory screening. Red circles indicate the position of structural modification/substitution during the metabolite-inspired analog design. The structure of MLY8 is shown as a representative analog. (**B**) NF-κB phenotypic screening. NF-κB/293 reporter cells were stimulated with tumor necrosis factor-α (TNF-α; 10 ng/mL, 18 h) in the presence of MLY compounds (20 μM). A total of 29 of 38 analogs (~76%) exhibited greater inhibitory activity than M13 at this screening concentration. The red dashed line indicates the NF-κB activity of M13 at 20 µM, used as the reference level for identifying analogs with greater inhibitory activity. Data are presented as mean ± SD (*n* = 3). Red circles indicate MLY2 and MLY8, which were selected as lead compounds for subsequent biological evaluation.

A focused library of 38 analogs (MLY1–38) was generated through systematic tripeptide editing of the glutathione moiety (Figure 1A). The MLY analogs were synthesized using a general Fmoc-based solid-phase peptide synthesis (SPPS) approach on Wang resin. Briefly, Fmoc-Cys(6-shogaol)-OH was coupled to amino acid-loaded resin, followed by sequential incorporation of the N-terminal amino acid residues and final cleavage/deprotection. The resulting compounds were purified by preparative reverse-phase high-performance liquid chromatography (HPLC). Their identities were confirmed by electrospray ionization mass spectrometry (ESI-MS) and ^1^H nuclear magnetic resonance (NMR) spectroscopy, and their purity (>95%) was confirmed by analytical HPLC. The structures of all analogs are summarized in Table S1, and detailed synthetic procedures and compound characterization data are provided in the Supplementary Materials. The central cysteine residue was retained, whereas the flanking amino acid residues were varied using naturally occurring amino acids spanning related physicochemical characteristics. This design aimed to maintain key structural and polarity features of M13 by substituting the flanking residues with amino acids of broadly similar physicochemical characteristics, while expanding accessible chemical space around the metabolite scaffold.

To evaluate anti-inflammatory activity, all analogs were screened using the NF-κB/293/GFP-Luc™ reporter system [22], a phenotypic assay that quantitatively measures NF-κB pathway activation following TNF-α stimulation. NF-κB signaling plays a central role in inflammatory amplification and cytokine production during intestinal inflammation [24,25,26], making this platform useful for identifying compounds capable of modulating inflammation-associated signaling networks. Treatment of NF-κB reporter cells with TNF-α induced robust pathway activation, which was substantially suppressed by multiple M13 analogs (Figure 1B).

**Figure 2:**
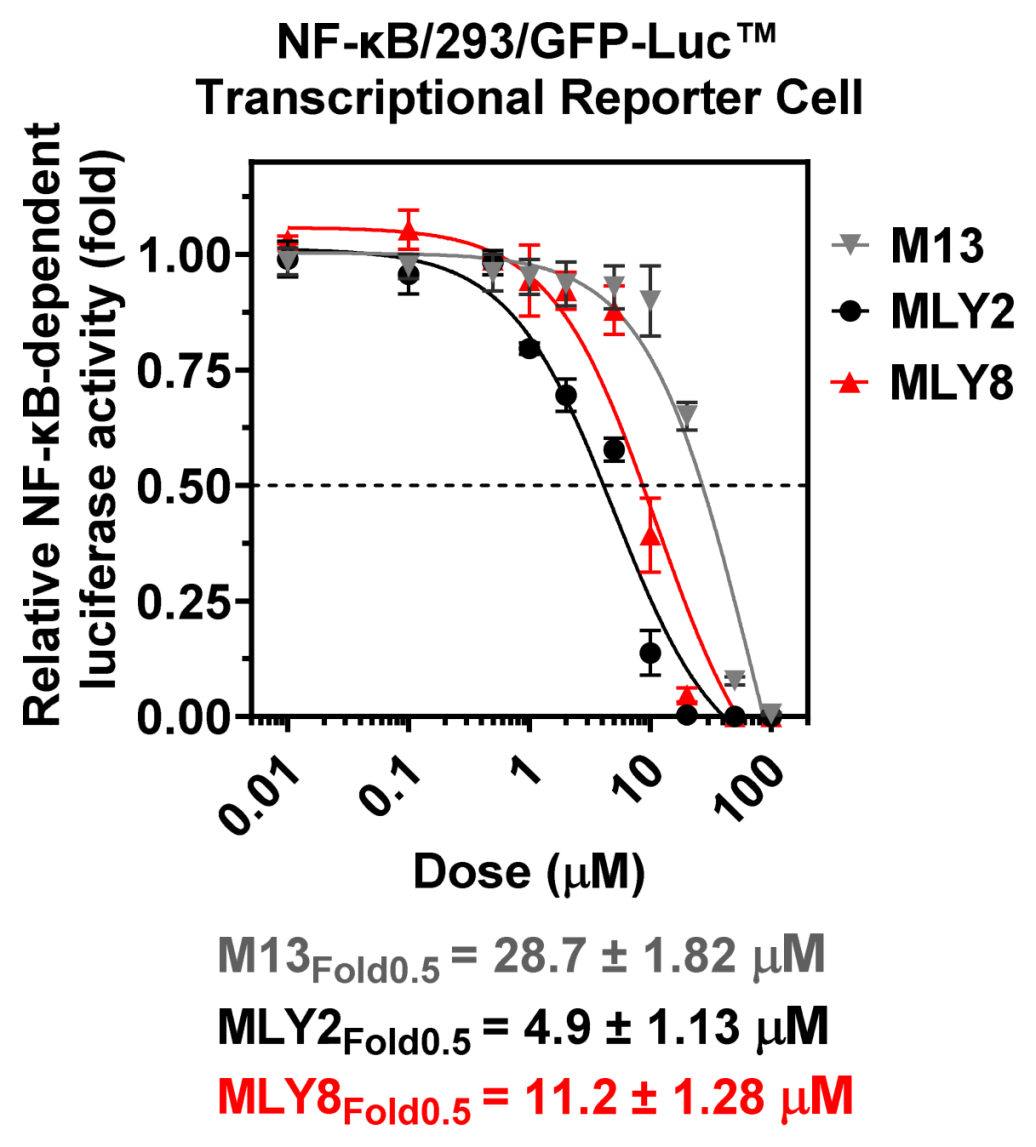
MLY2 and MLY8 exhibit concentration-dependent inhibition of NF-κB activation. NF-κB/293/GFP-Luc reporter cells were stimulated with TNF-α in the presence of increasing concentrations of M13, MLY2, or MLY8. Concentration–response analysis demonstrated greater NF-κB inhibitory activity of MLY2 and MLY8 than M13. Data are presented as mean ± SD (*n* = 3). The dashed line indicates 50% of control NF-κB activity. Fold0.5 denotes the concentration producing a 50% reduction in relative NF-κB-dependent luciferase activity.

At the 20 μM primary screening concentration, 29 of the 38 synthesized analogs (76%) produced greater NF-κB inhibition than M13, defined as non-overlapping mean ± SD relative to M13 (Figure 1B). These single-concentration data were used to identify and prioritize active analogs and were not interpreted as a quantitative ranking of potency across the full series. MLY2 and MLY8 were among the strongest hits, reducing NF-κB activation by approximately 99% and 92%, respectively, at 20 μM (residual activity 0.56 ± 0.62% and 8.04 ± 4.06% of control; Table S1). Structures of all MLY analogs and their corresponding screening activities are summarized in Table S1. Concentration–response analysis was subsequently performed for the selected leads and demonstrated estimated Fold0.5 values of approximately 5 μM for MLY2 and 11 μM for MLY8 (Figure 2), compared with the parent scaffold M13.

Structure–activity relationship (SAR) analysis of the 38-compound series demonstrated that NF-κB inhibitory activity was influenced by both terminal amino acid identity and linkage configuration. Within the Glu(α) series, for example, the Leu-containing MLY2 showed 0.56 ± 0.62% residual NF-κB activity, whereas the corresponding Met-containing MLY13 showed 60.22 ± 13.55% activity at 20 μM. Linkage-dependent differences were also evident among matched terminal residues; Leu-containing analogs ranged from 0.56 ± 0.62% for MLY2 [Glu(α)] to 61.27 ± 18.09% for MLY23 [Glu(γ)], with 38.60 ± 3.96% for MLY7 [Asp(α)] and 23.57 ± 6.50% for MLY33 [Asp(β)]. These findings indicate that activity depends on the combined influence of terminal residue identity and linkage configuration rather than on either structural variable alone. The structures and corresponding 20 μM NF-κB activities of all 38 analogs are provided in Table S1, with additional SAR analysis in the Supplementary Materials. Because the primary screen was conducted at a single concentration, these data define SAR based on relative activity at the screening concentration and are not interpreted as quantitative potency relationships.

Collectively, these findings demonstrate that phase II metabolite-inspired tripeptide editing can efficiently generate structurally diverse analogs with enhanced NF-κB inhibitory activity in a primary phenotypic screen. The high frequency of active compounds supports the use of the M13 scaffold as a productive starting point for lead identification, while the concentration–response studies of MLY2 and MLY8 provide quantitative characterization of the selected leads.

### 2.2. MLY2 and MLY8 Demonstrate Enhanced Anti-Inflammatory Activity in DSS-Induced Experimental Colitis

Following identification of MLY2 and MLY8 as two of the strongest hits in the NF-κB phenotypic screening, we next evaluated their anti-inflammatory activity in vivo using a DSS-induced experimental colitis model. DSS administration induces epithelial injury, mucosal barrier disruption, and inflammatory cytokine production, recapitulating several pathological features of human ulcerative colitis [27,28,29]. The present experiment was designed as a proof-of-concept comparison of the optimized analogs with the parent metabolite scaffold M13.

**Figure 3:**
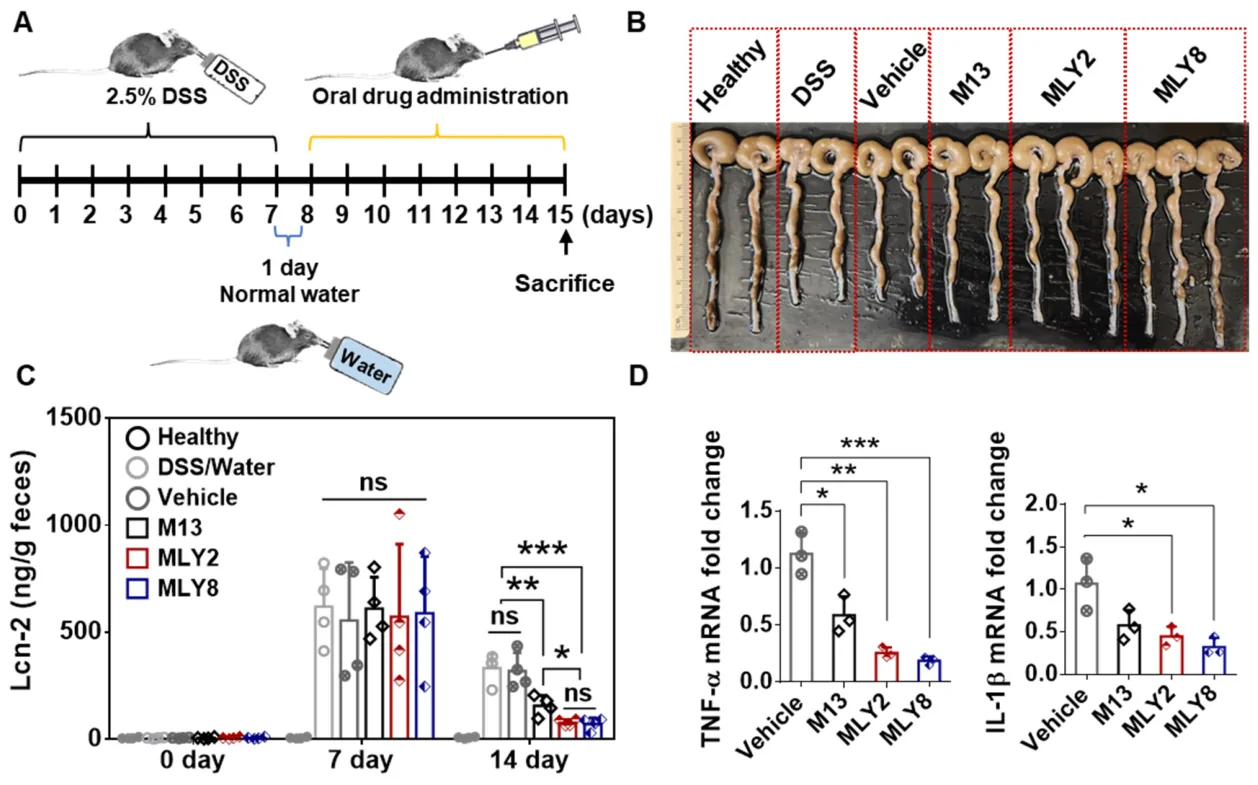
MLY2 and MLY8 demonstrate enhanced anti-inflammatory activity in DSS-induced experimental colitis. (**A**) Experimental timeline for DSS-induced colitis followed by oral treatment with M13, MLY2, or MLY8 during the recovery phase. (**B**) Representative colon lengths were measured as a macroscopic indicator of disease severity. (**C**) Fecal lipocalin-2 was quantified as a marker of intestinal inflammation (* *p* < 0.05, ** *p* < 0.01, *** *p* < 0.001, ns: nonsignificant; *n* = 4 per group). (**D**) Colonic inflammatory cytokine expression (TNF-α and interleukin-1β (IL-1β)) was assessed by qPCR in the DSS + vehicle, M13, MLY2, and MLY8 groups. Data are presented as mRNA fold changes relative to the DSS + vehicle group (* *p* < 0.05, ** *p* < 0.01, *** *p* < 0.001; *n* = 3 per group). Vehicle, phosphate-buffered saline (PBS) administered by oral gavage. In (**B**), the DSS/Water group is labeled “DSS.” Data are presented as mean ± SD. Groups were compared by the Kruskal–Wallis test with Dunn’s multiple comparisons test. Not all pairwise comparisons are annotated; comparisons relevant to the interpretation are shown.

Acute colitis was induced by administration of DSS in drinking water for 7 days, followed by a recovery phase during which mice received oral treatment with M13, MLY2, or MLY8 (Figure 3A). Colon length, fecal lipocalin-2 (Lcn-2), and inflammatory cytokine expression were subsequently assessed as indicators of intestinal inflammation and disease severity. Consistent with severe intestinal inflammation, DSS treatment resulted in marked colon shortening in vehicle-treated mice. Oral administration of M13 partially improved colon length, whereas treatment with either MLY2 or MLY8 produced substantially greater restoration of colon length toward levels observed in healthy control animals (Figure 3B). These findings suggest that tripeptide-edited analogs possess enhanced anti-inflammatory activity relative to the parent metabolite scaffold in vivo.

Fecal Lcn-2, a sensitive biomarker of intestinal inflammation, was markedly elevated following DSS treatment [30]. In contrast, treatment with MLY2 or MLY8 significantly reduced fecal Lcn-2 levels during the recovery phase, with greater suppression observed compared with M13-treated animals (Figure 3C). Similarly, analysis of colonic inflammatory cytokines demonstrated reduced expression of TNF-α and IL-1β following treatment with MLY2 or MLY8 (Figure 3D). These observations further support the enhanced anti-inflammatory activity of the tripeptide-edited analogs in experimental colitis.

Importantly, the enhanced anti-inflammatory activity of MLY2 and MLY8 observed in vivo was consistent with their activity in the NF-κB phenotypic screening assay. Improvements in colon length, fecal Lcn-2, and colonic cytokines provide complementary evidence of anti-inflammatory activity in DSS-induced colitis. Together, these findings demonstrate that tripeptide editing of the M13 scaffold generated lead compounds with enhanced in vivo anti-inflammatory activity relative to the parent metabolite.

### 2.3. MLY2 and MLY8 Suppress Inflammatory Cytokines in Treatment-Refractory Human UC Biopsies

To evaluate whether the lead compounds retained activity in human inflammatory tissue, we next examined MLY2 and MLY8 using ex vivo colonic biopsies obtained from patients with treatment-refractory ulcerative colitis. A substantial proportion of UC patients fail to respond adequately to currently available therapies, including biologics and small-molecule inhibitors [3,31,32]. Human biopsy-based explant systems provide a clinically relevant ex vivo platform for evaluating anti-inflammatory activity within patient-derived inflammatory microenvironments while preserving multicellular tissue architecture and endogenous signaling networks [33,34].

**Figure 4:**
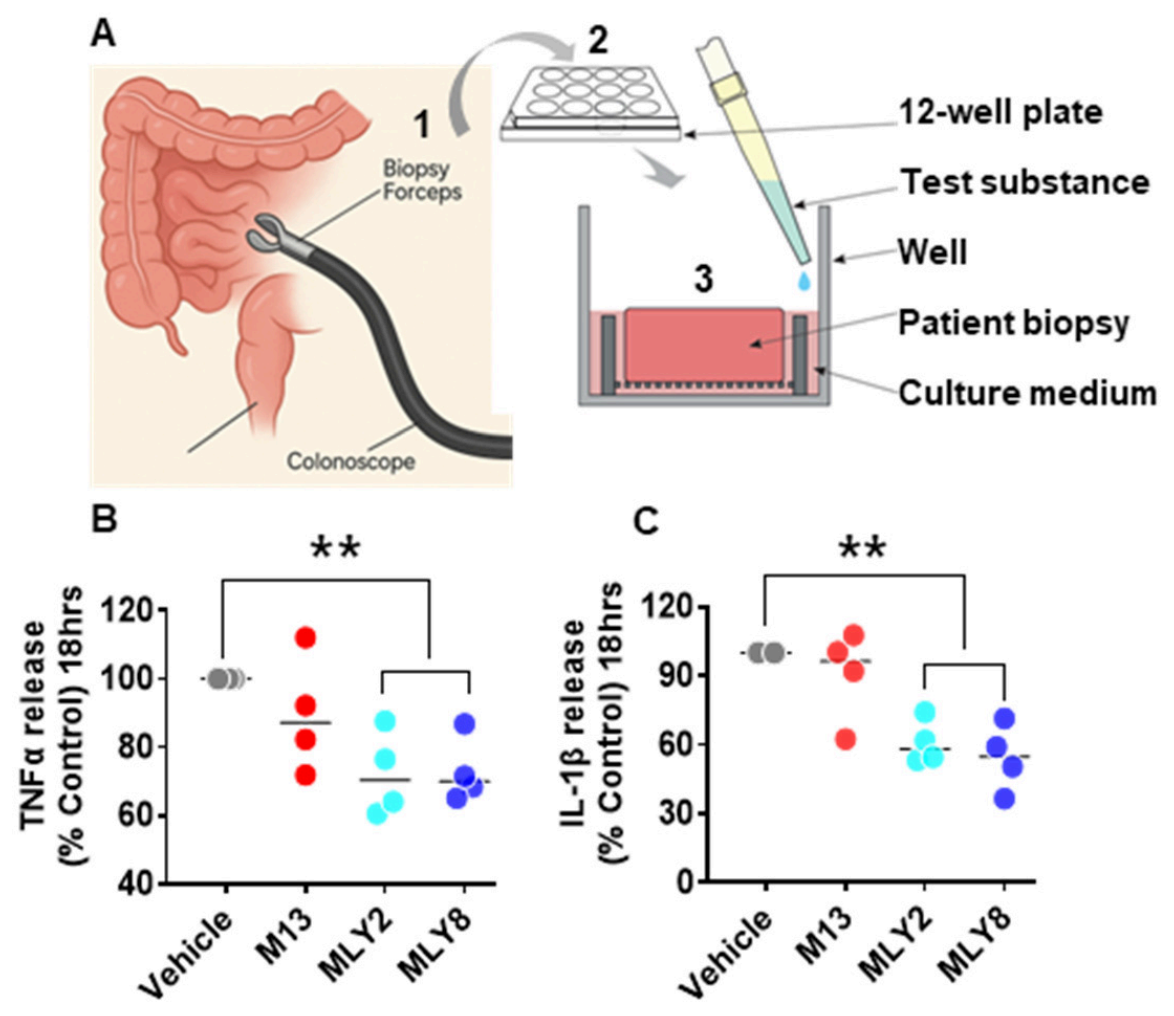
MLY2 and MLY8 suppress inflammatory cytokine production in ex vivo biopsies from patients with treatment-refractory UC. (**A**) Schematic of the ex vivo human biopsy culture workflow. 1. Gut mucosal biopsies were obtained from fresh UC tissue; 2. biopsies were placed in 12-well culture plates; and 3. biopsies were incubated with M13, MLY2, or MLY8. (**B**,**C**) MLY2 and MLY8 reduced production of the pro-inflammatory cytokines TNF-α and IL-1β in biopsies from patients with treatment-refractory UC. (Each dot represents the average value of 4 biopsies from one patient. ** *p* < 0.01, *n* = 4 patients per group.) Data are presented as mean ± SD. Groups were compared by the Kruskal–Wallis test with Dunn’s multiple comparisons test.

Colonic biopsies were obtained from patients with active UC who had previously failed multiple conventional therapies, including anti-TNF agents, integrin inhibitors, JAK inhibitors, and mesalamine-based treatments. Biopsies were cultured ex vivo in the presence or absence of M13, MLY2, or MLY8, and inflammatory cytokine production was subsequently quantified using multiplex cytokine analysis (Figure 4A).

Consistent with the in vitro phenotypic screening and in vivo DSS colitis results, both MLY2 and MLY8 demonstrated greater anti-inflammatory activity than M13 in patient-derived UC biopsies. Treatment with MLY2 or MLY8 significantly reduced production of the pro-inflammatory cytokines TNF-α and IL-1β relative to untreated controls, with stronger suppression observed compared with M13-treated tissues (Figure 4B,C). These findings demonstrate that the enhanced anti-inflammatory activities identified through phase II metabolite-inspired tripeptide editing were preserved within clinically relevant human inflammatory tissues.

Notably, although variability in cytokine magnitude was observed among individual patient samples, suppression of IL-1β was consistently detected across multiple biopsies, suggesting the presence of convergent inflammatory signaling responses despite underlying patient heterogeneity. In contrast, TNF-α responses exhibited greater inter-patient variability, consistent with the heterogeneous inflammatory programs frequently observed in UC patients with refractory disease [28]. These findings suggest that MLY2 and MLY8 may preferentially modulate conserved innate inflammatory amplification pathways that remain active across heterogeneous patient inflammatory states.

The ability of MLY2 and MLY8 to suppress inflammatory cytokine production in biopsies from patients unresponsive to multiple conventional therapies provides complementary evidence that the compounds retain anti-inflammatory activity in complex human inflammatory tissue. This ex vivo finding supports further investigation of the MLY scaffold in clinically relevant human-tissue models and subsequent preclinical studies.

### 2.4. Effects of MLY2 and MLY8 on Human PBMC Viability and T-Cell Populations

Given the central role of immune cells in inflammatory regulation and the potential risk of immunotoxicity associated with electrophilic anti-inflammatory compounds, we next performed preliminary immune tolerability studies using human peripheral blood mononuclear cells (PBMCs) [21]. PBMC-based assays provide a useful primary-human-cell platform for evaluating potential cytotoxic or immunomodulatory liabilities during early lead characterization [35,36].

Human PBMCs obtained from three independent donors were treated with MLY2 or MLY8 at concentrations of 5 or 20 μM for 4 days under resting or IL-2–stimulated conditions, followed by multiparameter flow cytometric analysis of lymphocyte viability and major T-cell populations. Gating strategies included sequential identification of viable lymphocytes, singlets, CD14− lymphocyte populations, and downstream CD4+ and CD8+ T-cell subsets using 7-AAD viability staining and fluorescence-based immune phenotyping.

**Figure 5:**
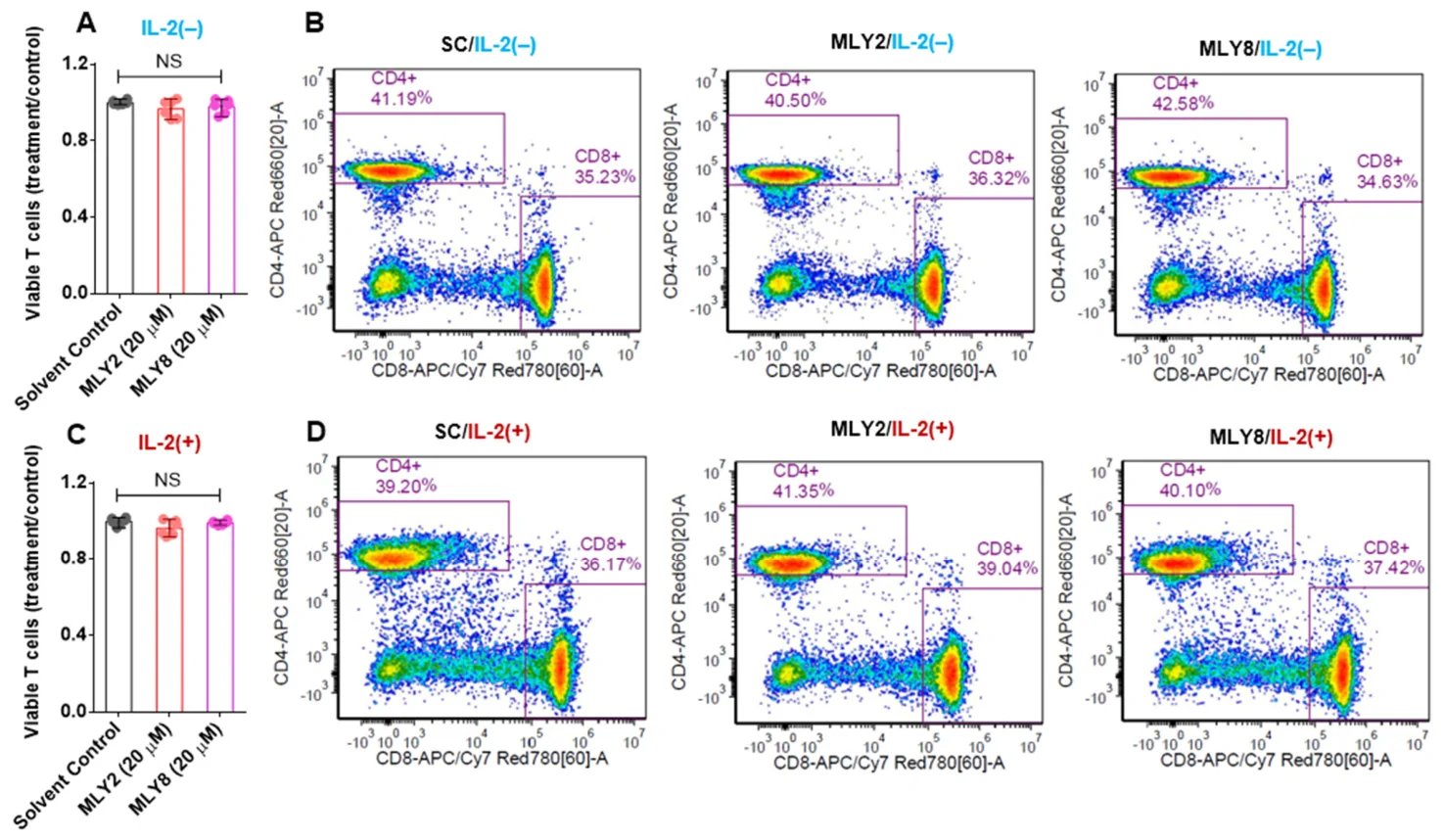
Effects of MLY2 and MLY8 on viable lymphocyte populations and major T-cell subsets in human PBMCs. Human PBMCs from three independent donors were treated with MLY2 or MLY8 at 5 or 20 µM for 4 days and analyzed by flow cytometry. (**A**) Viable T-cell populations under resting (IL-2−) conditions. (**B**) Representative flow cytometry plots showing CD4+/CD8+ T-cell distributions within viable CD14− lymphocytes under resting (IL-2−) conditions. (**C**) Viable T-cell populations under IL-2–stimulated conditions. (**D**) Representative flow cytometry plots showing CD4+/CD8+ T-cell distributions under IL-2–stimulated conditions. In (**B**,**D**), color intensity represents event density in the flow cytometry plots. Across donors, MLY2 and MLY8 did not induce major reductions in viable lymphocyte populations or substantial disruption of CD4+ and CD8+ T-cell populations at concentrations up to 20 µM (ns, nonsignificant; *n* = 3 biological replicates per group, representing 3 independent donors, with each donor tested in duplicate).

Across all three donors, treatment with MLY2 or MLY8 at either 5 or 20 μM did not induce meaningful reductions in viable lymphocyte populations relative to solvent-treated controls under either resting (IL-2−) or IL-2–stimulated conditions (Figure 5A,C). Likewise, CD4+ helper T-cell and CD8+ cytotoxic T-cell populations remained broadly preserved across treatment groups (Figure 5B,D). Although donor-to-donor variability was observed, no consistent evidence of lymphocyte depletion, major CD4+/CD8+ population disruption, or overt acute cytotoxicity was detected at these concentrations.

Collectively, no overt reduction in viable lymphocyte populations or major disruption of CD4+ and CD8+ T-cell subsets was observed following treatment with MLY2 or MLY8 in PBMCs from three independent donors, supporting favorable preliminary immune-cell tolerability under the conditions tested.

### 2.5. MLY2 and MLY8 Exhibit No Detectable Mutagenicity in Ames Assays

As part of preliminary lead characterization, the mutagenic potential of MLY2 and MLY8 was evaluated using the Ames bacterial reverse mutation assay with *Salmonella typhimurium* tester strains TA98 and TA100, which detect frameshift and base-pair substitution mutations, respectively [37]. Sodium azide (NaN_3_) and 2-nitrofluorene (2NF) were used as positive mutagenic controls for TA100 and TA98 strains, respectively, under non-activated (−S9) conditions, and 2-aminoanthracene (2AA) served as the positive control for both strains under metabolically activated (+S9) conditions.

**Figure 6:**
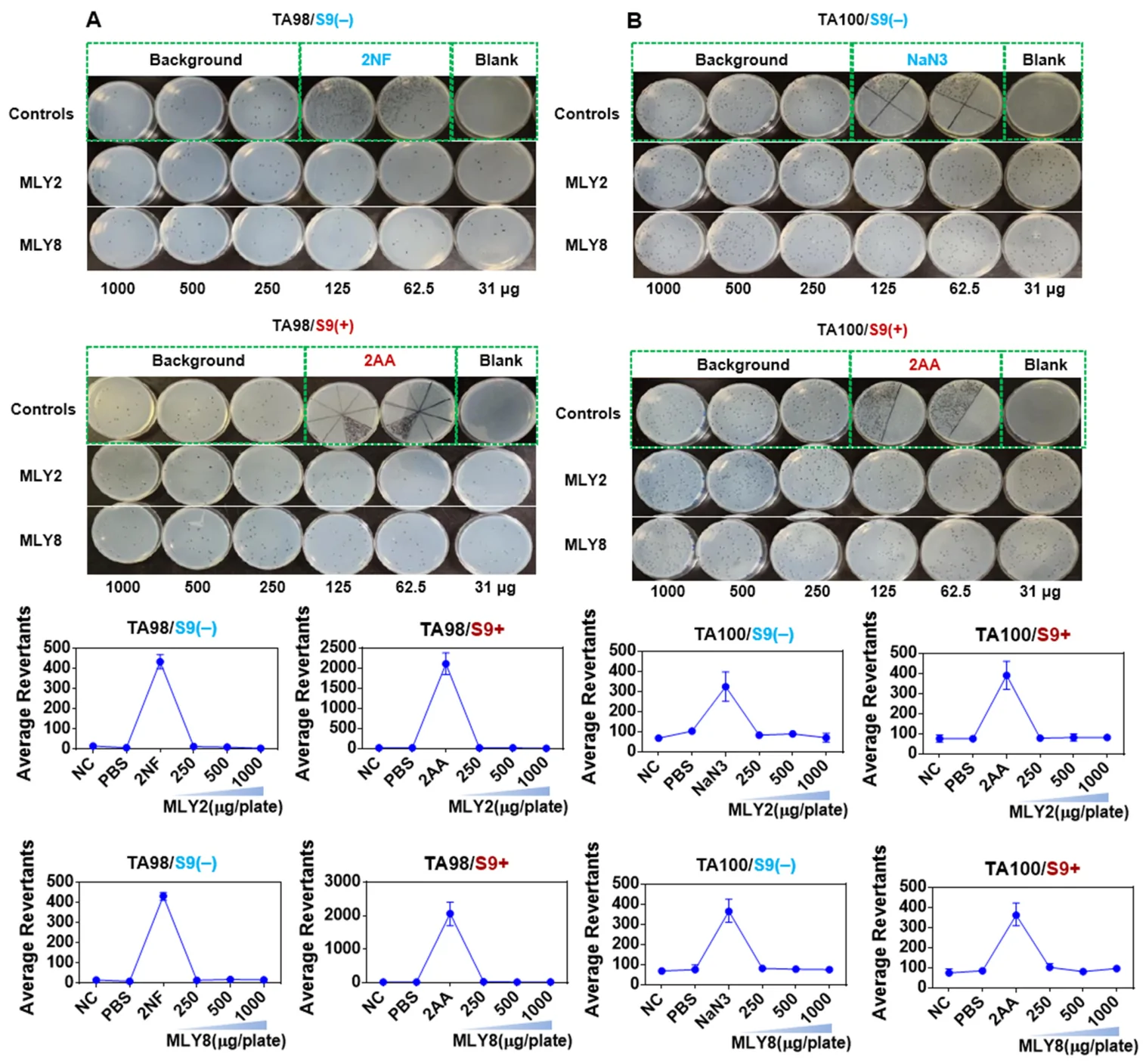
MLY2 and MLY8 exhibit no detectable mutagenic activity in Ames assays. Mutagenic potential was evaluated using *Salmonella typhimurium* tester strains (**A**) TA98 and (**B**) TA100 under both non-activated (−S9) and metabolically activated (+S9) conditions. For each strain, representative plates (**top**) and revertant colony quantification (**bottom**) are shown for MLY2 and MLY8 across the tested concentration range (31–1000 μg/plate). 2-Nitrofluorene (2NF; TA98) and sodium azide (NaN_3_; TA100) served as positive controls under −S9, and 2-aminoanthracene (2AA) for both strains under +S9; vehicle (PBS) and negative controls were included on each plate set. Data are presented as mean ± SD (*n* = 3).

Across the tested concentration range, neither MLY2 nor MLY8 increased revertant colony formation relative to vehicle-treated controls in either tester strain with or without metabolic activation (Figure 6A,B). In contrast, the corresponding positive controls produced robust increases in revertant colony numbers, confirming appropriate assay performance and mutagen sensitivity. No visible evidence of severe bacterial toxicity or abnormal background lawn disruption was observed at the tested concentrations. These findings indicate that MLY2 and MLY8 do not exhibit detectable mutagenic activity in TA98 and TA100 under the tested conditions, either in the absence or presence of S9 metabolic activation.

### 2.6. Single-Dose Oral Tolerability of MLY2 and MLY8 in Mice

**Figure 7:**
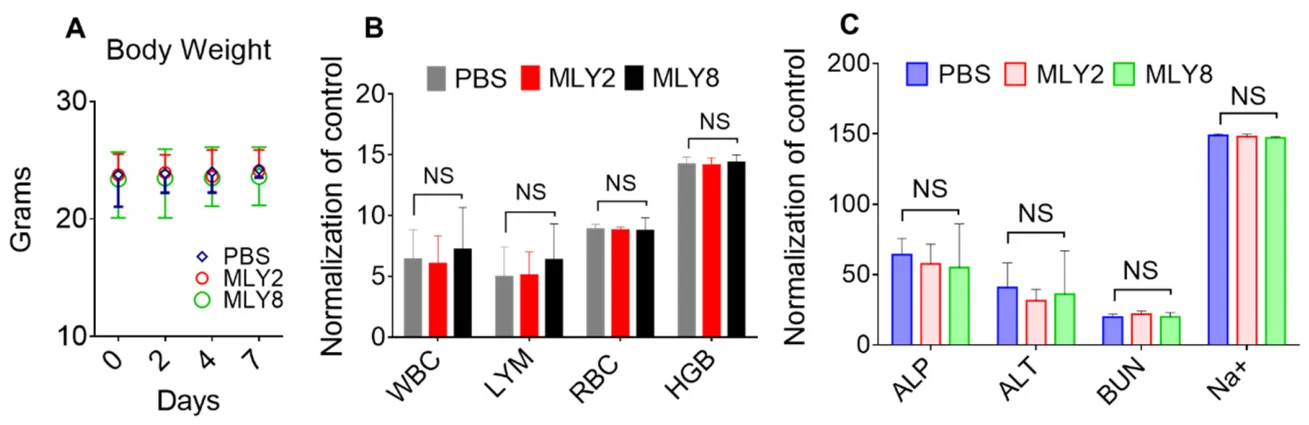
Single-dose oral tolerability of MLY2 and MLY8 in mice. Female CD-1 mice received a single oral dose of vehicle, MLY2, or MLY8 at 1000 mg/kg and were monitored for 7 days. (**A**) Body weight changes during the observation period. All groups exhibited gradual body weight increases without significant differences between treatment and vehicle groups. (**B**) Hematological parameters and (**C**) serum biochemical markers were evaluated at study endpoint. No major treatment-related abnormalities were observed relative to vehicle-treated controls. Data are presented as mean ± SD; ns, nonsignificant; *n* = 5 per group. Groups were compared by the Kruskal–Wallis test with Dunn’s multiple comparisons test.

To further evaluate the preliminary in vivo safety and tolerability of the lead compounds identified through phase II metabolite-inspired tripeptide editing, we performed a single-dose maximum tolerated dose (MTD) study in mice, building on our group’s prior oral MTD-based safety evaluation [38]. Oral administration of MLY2 or MLY8 at 1000 mg/kg did not produce significant acute toxicity during the observation period. All animals survived throughout the study, and no significant abnormal behavioral changes or overt clinical signs of toxicity were observed. Body weight remained generally stable, with no major body weight loss or gain detected relative to vehicle-treated controls. Food and water consumption also remained broadly comparable across treatment groups during the study period. Hematological and serum biochemical analyses similarly did not reveal major alterations in measured parameters relative to control animals (Figure 7). These findings support favorable preliminary in vivo tolerability profiles for both compounds under the experimental conditions evaluated.

Collectively, the PBMC, Ames, and single-dose MTD studies demonstrated favorable preliminary tolerability of MLY2 and MLY8, with no overt immune-cell toxicity, detectable mutagenic activity, or major acute toxicity observed under the conditions tested. Importantly, the enhanced biological activity achieved through phase II metabolite-inspired tripeptide editing was not accompanied by detectable adverse effects in these preliminary assessments. These results support MLY2 and MLY8 as promising anti-inflammatory leads for further preclinical characterization.

## 3. Discussion

Phase II metabolism has historically been viewed primarily as a detoxification process that facilitates drug inactivation and elimination [8]. In contrast, the present study demonstrates that biologically active phase II metabolites may also serve as a chemically productive starting point for medicinal chemistry optimization and lead discovery. Using the glutathione-conjugated 6-shogaol metabolite M13 as a lead scaffold, systematic tripeptide editing generated a high frequency of analogs with enhanced anti-inflammatory activity, with 29 of 38 compounds outperforming the parent metabolite in the NF-κB phenotypic screening platform at the screening concentration. These findings suggest that phase II metabolite scaffolds may possess substantial structural flexibility while retaining biologically favorable features.

An important aspect of this strategy was preservation of the central cysteine-containing 6-shogaol-derived scaffold while systematically modifying the flanking amino acid residues of the glutathione tripeptide. This approach aimed to maintain key structural and polarity features of M13 while expanding accessible chemical space around the metabolite scaffold. The high proportion of active analogs identified in this study further suggests that biologically active phase II metabolites may represent underexplored starting points for generating structurally diverse compounds with enhanced biological activity. Although the precise molecular target was not defined in this study, the observed suppression of NF-κB signaling is consistent with previous studies implicating the electrophilic 6-shogaol-derived α,β-unsaturated carbonyl (Michael acceptor) moiety in modulation of redox-sensitive inflammatory signaling pathways [39]. Such cysteine-directed electrophilic modification together with Keap1–Nrf2-dependent antioxidant signaling has been described as an anti-inflammatory mechanism among Michael-acceptor natural products and their cysteine-conjugated metabolites [20,40]. These observations provide a mechanistic rationale for retaining this structural feature during analog design; however, its direct contribution to the activity of MLY2 and MLY8 remains to be established.

Rather than relying exclusively on individual target-based optimization, we employed an NF-κB-centered phenotypic screening strategy to identify compounds capable of suppressing inflammatory pathway activation at the cellular level. Phenotypic screening approaches can capture integrated biological responses within complex signaling networks and may facilitate identification of compounds with relevant biological activities even when the precise molecular targets remain incompletely defined [41]. The enhanced anti-inflammatory activity identified in the NF-κB reporter assay was subsequently observed in DSS-induced experimental colitis, where MLY2 and MLY8 improved multiple inflammatory readouts relative to the parent metabolite M13. Importantly, both compounds also retained anti-inflammatory activity in patient-derived biopsies from treatment-refractory UC, providing complementary evidence across cellular, animal, and human-tissue experimental systems.

The ex vivo biopsy findings are particularly notable given the substantial heterogeneity that characterizes refractory UC. Although cytokine responses varied among individual patient samples, suppression of IL-1β signaling was consistently observed across multiple biopsies, suggesting that MLY2 and MLY8 may modulate conserved inflammatory pathways that remain active across heterogeneous inflammatory states. In contrast, TNF-α responses demonstrated greater inter-patient variability, consistent with the complex and patient-specific inflammatory programs associated with UC progression and therapeutic resistance. At the same time, ex vivo biopsy models do not reproduce intestinal absorption, systemic pharmacokinetics, metabolism, immune-cell trafficking, or long-term drug exposure. Thus, these experiments are most appropriately viewed as evidence of activity in patient-derived inflammatory tissue rather than as a surrogate for clinical efficacy.

The selected compounds MLY2 and MLY8 also demonstrated favorable preliminary tolerability across the assays performed. No overt disruption of lymphocyte viability or major T-cell populations was observed in PBMCs from three independent donors; neither compound showed detectable mutagenic activity in TA98 or TA100 with or without S9 metabolic activation, and both compounds were well tolerated following single-dose oral administration in mice at the doses evaluated. These complementary assessments provide useful preliminary information for lead characterization. The PBMC donor number was limited, the TA98/TA100 Ames assay does not constitute a comprehensive genotoxicity assessment, and the single-dose MTD experiment cannot address potential effects associated with repeated or prolonged exposure. Accordingly, broader immunotoxicity, genotoxicity, and repeat-dose safety assessments will be important components of subsequent characterization.

Several limitations of this exploratory proof-of-concept study should be acknowledged. **(1)** The proposed contribution of electrophilic reactivity remains inferential. Direct interaction of MLY2 or MLY8 with cysteine-containing targets was not demonstrated, and glutathione depletion, N-acetylcysteine rescue, Nrf2 activation, and direct target engagement were not examined. **(2)** The chemical and metabolic stability of the compounds during gastrointestinal transit was not evaluated. The potential for acid-, enzyme-, or microbiota-mediated transformation of the conjugates, including deconjugation under anaerobic intestinal conditions and possible release of 6-shogaol-derived species, therefore remains to be determined. **(3)** The in vivo safety assessment was limited to a single-dose MTD study and did not include repeat-dose or chronic toxicological evaluation. **(4)** Pharmacokinetic and biodistribution data were not obtained, leaving systemic exposure, colonic accumulation, and metabolic fate undefined. **(5)** The DSS experiment was designed as a proof-of-concept comparison of the optimized analogs with the parent metabolite M13 and did not include a standard-of-care comparator; accordingly, the present findings should not be interpreted as demonstrating superiority to approved UC therapies. **(6)** The ex vivo patient-biopsy cohort was small, which constrains the generalizability of the human tissue findings. **(7)** Mice were group-housed, and treatment groups were maintained in separate cages; cage and treatment group therefore coincided, and cage-level effects could not be formally separated from treatment effects at the sample size used. All cages were maintained under identical housing conditions and underwent identical treatment and measurement procedures, and all outcome measures were determined individually for each mouse, which was treated as the experimental unit for analysis. The study was not blinded. Additional studies using larger and more clinically diverse cohorts will be valuable for determining the generalizability of the observed human-tissue responses. These limitations reflect areas for subsequent mechanistic and preclinical characterization and do not alter the primary objective of the present study, which was to determine whether systematic editing of a biologically active phase II metabolite scaffold could generate anti-inflammatory leads with improved activity.

## 4. Materials and Methods

### 4.1. Chemical Synthesis and Characterization of M13 Analogs

M13 analogs (MLY1–38) were generated through systematic tripeptide editing of the glutathione-conjugated M13 scaffold. The synthesis of M13 was performed as previously described with modifications [17,18]. All compounds were synthesized according to procedures designed by the authors and executed by BOC Sciences (Shirley, NY, USA). Briefly, MLY analogs were synthesized using Fmoc-based solid-phase peptide synthesis (SPPS) on Wang resin by preserving the central cysteine residue and systematically replacing the flanking amino acid residues of the glutathione tripeptide with selected naturally occurring amino acids while seeking to preserve key structural and polarity characteristics of M13. The final compounds were purified by preparative reverse-phase HPLC, and their identities were confirmed by ESI-MS and ^1^H NMR spectroscopy, while their purity (>95%) was confirmed by analytical HPLC. Detailed synthetic procedures, reaction schemes, structures, compound identifiers, analytical characterization, and the corresponding 20 μM NF-κB screening activities are provided in the Supplementary Materials to facilitate structure–activity relationship (SAR) analysis across the 38 compounds.

### 4.2. NF-κB Reporter Assay

Anti-inflammatory activity was evaluated using the NF-κB/293/GFP-Luc™ reporter cell line (System Biosciences [SBI], Palo Alto, CA, USA; Catalog No. TR860A-1) [22], which quantitatively measures NF-κB pathway activation. Cells were cultured according to the manufacturer’s instructions and stimulated with TNF-α (10 ng/mL) for 18 h in the presence or absence of test compounds. Luciferase activity was quantified using a Synergy H4 multi-mode microplate reader (BioTek/Agilent Technologies, Santa Clara, CA, USA) and normalized relative to TNF-α-stimulated controls. For primary screening, compounds were tested at 20 μM. All NF-κB reporter experiments were performed with *n* = 3 independent replicates per condition. Concentration–response studies were subsequently performed for selected lead compounds. Statistical analyses, where applicable, were performed as described in Section 4.10.

### 4.3. DSS-Induced Experimental Colitis

Female C57BL/6 mice (Mus musculus; 6–8 weeks old; Jackson Laboratory, Bar Harbor, ME, USA) were housed under specific pathogen-free (SPF) conditions with a 12-h light/dark cycle and acclimatized for one week prior to experimentation. All animals were wild-type, had no prior procedures, and were in good health. Mice were received from the vendor at similar body weights and housed five per cage, and cages were randomly assigned to control or treatment groups. All cages were maintained under identical housing conditions and underwent the same treatment and measurement procedures. Mice were assigned to six experimental groups (*n* = 5 mice per group): healthy control, DSS/Water, DSS + vehicle, DSS + M13, DSS + MLY2, and DSS + MLY8. Experimental colitis was induced by administration of 2.5% dextran sulfate sodium (DSS; MP Biomedicals, Irvine, CA, USA; CAS 9011-18-1; molecular weight 36,000–50,000 Da) in drinking water for 7 days, followed by one day of normal drinking water and then a recovery phase during which M13, MLY2, or MLY8 (2.5 mg/kg in PBS, 0.2 mL) was administered by oral gavage once daily for an additional 7 days, and animals were euthanized on the last day [27]. All groups except the healthy control received DSS during the induction phase and normal drinking water thereafter. During the recovery phase the DSS/Water group received no gavage, whereas the DSS + vehicle group received a daily oral gavage of PBS to control for the gavage procedure itself. Disease severity was evaluated using colon length measurements, fecal lipocalin-2 (Lcn-2) quantification [30], and inflammatory cytokine analysis. Colon tissues were collected at study endpoint for downstream molecular analyses. Each mouse was treated as an independent experimental unit (*n* = 5 per group). No pre-established inclusion or exclusion criteria were defined, and all animals were monitored for health and behavior throughout the study. All animals survived to the study endpoint in good health, and no animals were excluded from the study. Colon length was assessed in all animals, fecal lipocalin-2 in four animals per group, and colonic cytokine expression in three animals per group. A total of 30 mice were used in the DSS study and 45 mice across all animal experiments in this work (DSS, 30; MTD, 15). Significance was interpreted using the criteria defined in Section 4.10 (* *p* < 0.05, ** *p* < 0.01, *** *p* < 0.001; ns, *p* ≥ 0.05).

### 4.4. Human UC Biopsy Culture

De-identified colonic biopsies from patients with active treatment-refractory ulcerative colitis were obtained through a commercial tissue procurement protocol and ex vivo testing service (REPROCELL Europe Limited, Glasgow, UK). Patients included individuals previously unresponsive to conventional IBD therapies, including anti-TNF agents, integrin inhibitors, JAK inhibitors, and mesalamine-based therapies. Biopsies from four independent patients (*n* = 4 patients per treatment group) were cultured ex vivo in the presence or absence of M13, MLY2, or MLY8 (25 μM final concentration) for approximately 18 h under high-oxygen culture conditions. These short-term culture conditions were selected on the basis of established ex vivo intestinal mucosal biopsy methodology designed to preserve tissue viability and inflammatory readouts during drug exposure [33]. For each patient and treatment condition, four biopsy specimens were analyzed and averaged to generate one patient-level value. The patient, rather than the individual biopsy, was treated as the independent experimental unit. Culture supernatants were subsequently collected for cytokine analysis. The specimens supplied to the investigators were de-identified; patient-level clinical variables not included in the procurement records available to the investigators are therefore not inferred or reconstructed. Tissue was collected under the supplier’s ethically approved surplus-tissue protocol (REC 22-WS-0007; IRAS ID 310642), under which all donors provided written informed consent for the research use of residual tissue. The use of de-identified human colonic biopsy specimens in this study was reviewed and approved as exempt research by the Institutional Review Board of Georgia State University (Exempt Category 4; IRB No. H27079).

### 4.5. Multiplex Cytokine Analysis

Inflammatory cytokines in biopsy culture supernatants were quantified using multiplex bead-based immunoassays according to the manufacturer’s instructions [42]. TNF-α, IL-1β, and additional inflammatory mediators were quantified on a Luminex 200 system (Luminex Corporation, Austin, TX, USA) using an R&D Systems (Minneapolis, MN, USA) multiplex immunoassay (Catalog # LUHM000). Cytokine concentrations were calculated by interpolation against standard calibration curves generated on the same assay plate. For the UC biopsy study, data were summarized at the patient level (*n* = 4 independent patients per treatment group), with each patient-level value representing the average of four biopsies from that patient. Statistical analyses were performed as described in Section 4.10.

### 4.6. Evaluation of Immunotoxicity of MLY2 and MLY8 in Human PBMCs

Cryopreserved human peripheral blood mononuclear cells (PBMCs) from three independent donors were thawed and cultured in ImmunoCult™-XF T Cell Expansion Medium (STEMCELL Technologies, Vancouver, BC, Canada). Cells were labeled with Cell Proliferation Dye eFluor™ 450 (eBioscience/Thermo Fisher Scientific, Waltham, MA, USA) and treated with MLY2 or MLY8 (5 or 20 μM) or vehicle control (0.1% DMSO) for 4 days in the presence or absence of stimulation with recombinant human IL-2 (10 ng/mL; STEMCELL Technologies, cat. no. 78036.1). An ImmunoCult™-XF CD3/CD28 T Cell Activator was included as a positive control for T-cell activation.

At the end of the incubation period, cells were stained with antibodies against CD14, CD4, and CD8 (BioLegend, San Diego, CA, USA) together with 7-AAD viability dye (Sigma-Aldrich, Burlington, MA, USA) and analyzed on a CytoFLEX flow cytometer (Beckman Coulter Life Sciences, Brea, CA, USA). Total cellular events were first visualized using forward-scatter area (FSC-A) versus side-scatter area (SSC-A). Viable lymphocytes were then identified using FSC-A versus 7-AAD, with 7-AAD-negative events retained for subsequent analysis. Singlets were selected from the viable-lymphocyte population using FSC-A versus FSC-H. Within the singlet population, CD14-negative cells were identified using CD14 versus SSC-A to exclude CD14-positive monocytic cells. CD4+ and CD8+ T-cell populations were subsequently identified within the CD14-negative population, and eFluor™ 450 fluorescence was evaluated separately within the CD4+ and CD8+ populations for assessment of cell proliferation. Data from three independent donors (*n* = 3) were used to evaluate the effects of MLY2 and MLY8 on lymphocyte viability and major T-cell subset distribution; each donor was treated as an independent experimental unit. The representative gating strategy is provided in the Supplementary Materials as Figure S42.

### 4.7. Ames Mutagenicity Assay

Mutagenic potential was evaluated using the Ames bacterial reverse mutation assay with *Salmonella typhimurium* tester strains TA98 (Fisher Scientific, Waltham, MA, USA, cat. no. 50-948-937) and TA100 (Fisher Scientific, cat. no. 50-948-938) [37]. Compounds were tested across a range of concentrations (31–1000 μg/plate) both with and without phenobarbital/β-naphthoflavone-induced Sprague–Dawley rat liver S9 metabolic activation (Fisher Scientific, cat. no. 50-948-579), alongside vehicle and strain-appropriate positive controls (2-nitrofluorene for TA98 and sodium azide for TA100 under −S9; 2-aminoanthracene for both strains under +S9). Each experimental condition was evaluated in triplicate (*n* = 3). Revertant colonies were quantified after incubation, and mutagenic activity was assessed relative to control groups. The current dataset was interpreted qualitatively and by revertant-colony counts as shown in Figure 6.

### 4.8. Single-Dose Maximum Tolerated Dose (MTD) Study of MLY2 and MLY8

Female CD-1 mice (Mus musculus; 8 weeks old; Jackson Laboratory, Bar Harbor, ME, USA) were randomly assigned to three groups (*n* = 5 per group). Mice in Group 1 received vehicle control (2% sodium carboxymethyl cellulose, CMC-Na; Sigma-Aldrich, Burlington, MA, USA), whereas mice in Groups 2 and 3 received a single oral dose of MLY2 or MLY8, respectively, formulated in 2% CMC-Na suspension at 1000 mg/kg on day 0 [38]. Following administration, all animals were maintained under standard conditions with free access to food and water for 7 days. Mice were monitored daily for mortality, clinical signs of toxicity, body weight changes, and food consumption throughout the study period. Humane endpoints were defined as body weight loss exceeding 20% or the appearance of a hunched posture; no animal reached these endpoints during the study. Blood samples were collected prior to euthanasia on day 7 for hematological and serum biochemical analyses. Each mouse was treated as an independent experimental unit (*n* = 5 per group). Statistical comparisons were performed as described in Section 4.10; *p* ≥ 0.05 was considered not significant (ns), consistent with Figure 7.

### 4.9. Hematological and Biochemical Analysis of the Blood

Before euthanasia, female CD-1 mice from the single-dose MTD study (*n* = 5 mice per group) were anesthetized with 3% isoflurane, and about 0.3–0.4 mL of fresh blood was immediately collected via the retro-orbital sinus by blood-collecting tubes. Then hematological analyses were performed using an automatic hematology analyzer (VetScan HM5; Abaxis, Union City, CA, USA), and biochemical analyses were performed using a biochemical analyzer (VetScan VS2; Abaxis, Union City, CA, USA) with a comprehensive diagnostic profile reagent rotor. Each mouse was treated as an independent experimental unit, and statistical analyses were performed as described in Section 4.10.

### 4.10. Statistical Analysis

Data are presented as mean ± SD unless otherwise indicated. Statistical analyses were performed using GraphPad Prism version 6.0.1 (GraphPad Software, San Diego, CA, USA). The independent experimental unit was the individual mouse for animal studies, the individual patient for the ex vivo UC biopsy study, and the individual PBMC donor for the PBMC study. For the biopsy experiment, four biopsies from each patient under a given condition were averaged to generate one patient-level value (*n* = 4 patients per treatment group), thereby avoiding treatment of technical biopsy replicates as independent observations. For PBMC experiments, each of the three donors was treated as one biological replicate. The 38-compound NF-κB experiment at 20 μM was used as a primary activity screen and was not used to infer quantitative comparative potency across the series; concentration–response analysis was performed for selected leads. Because the biological sample sizes used in this study are small, normality could not be reliably assessed, and nonparametric methods were therefore used throughout. Group comparisons were analyzed by the Kruskal–Wallis test followed by Dunn’s multiple comparisons test. Differences were considered statistically significant at *p* < 0.05. Figure annotations were defined as * *p* < 0.05, ** *p* < 0.01, *** *p* < 0.001, and ns, *p* ≥ 0.05. Sample sizes for the animal studies (*n* = 5 per group) were based on standard practice for these models and were not derived from an a priori power calculation. Investigators were not blinded to group allocation during the conduct of the experiments, outcome assessment, or data analysis. Results are interpreted cautiously and in conjunction with the biological effect patterns.

## 5. Conclusions

In summary, this work establishes phase II metabolite-inspired tripeptide editing as a productive strategy for phenotypic lead discovery and identifies MLY2 and MLY8 as anti-inflammatory leads with activity in cellular, DSS-colitis, and ex vivo human UC biopsy models. The present findings provide a foundation for subsequent mechanistic, pharmacokinetic, stability, comparative-efficacy, and repeat-dose safety studies. More broadly, biologically active phase II metabolites may represent underutilized scaffolds for medicinal chemistry optimization in inflammatory disease.

## Data Availability

The data underlying this article will be shared on reasonable request to the corresponding author.

## Supplementary Materials

The following supporting information can be downloaded at https://www.mdpi.com/article/10.3390/ijms27188285/s1.
