# Supplementary material for: Phase II Metabolite-Inspired Tripeptide Editing Generates Anti-Inflammatory Candidates for Treatment-Refractory Ulcerative Colitis
Source: Int J Mol Sci. 2026 Sep 17;27(18):8285. doi: 10.3390/ijms27188285 (PMC13607239; doi:10.3390/ijms27188285)

Supplementary Materials

Phase II Metabolite-Inspired Tripeptide Editing Generates Anti-Inflammatory Candidates for Treatment-Refractory Ulcerative Colitis

Xiaodi Shi [1], Rabeya Jafrin Mow [1], Dingpei Long [1], Olivier Merlin-Zhang [2], Emma Xu [2], Xiaomeng Shi [1], Pallavi Garg [1], Shanthi Srinivasan [3,4] and Chunhua Yang [1,*]

1 Digestive Disease Research Group, Institute for Biomedical Sciences, Georgia State University, Atlanta, GA 30303, USA; xshi10@student.gsu.edu (X.S.); rmow1@student.gsu.edu (R.J.M.); dlong26@gsu.edu (D.L.); xshi8@gsu.edu (X.S.); pgarg@gsu.edu (P.G.)
2 School of Medicine, Wake Forest University, Winston-Salem, NC 27101, USA; olivier.merlinzhang@wfusm.edu (O.M.-Z.); emma.xu@wfusm.edu (E.X.)
3 Division of Digestive Diseases, Emory University School of Medicine, Atlanta, GA 30307, USA; ssrini2@emory.edu
4 Atlanta Veterans Affairs Medical Center, Decatur, GA 30302, USA
* Correspondence: cyang16@gsu.edu

Methods

General Semisynthetic Procedure for MLY Analogs (Representative Example: MLY8)

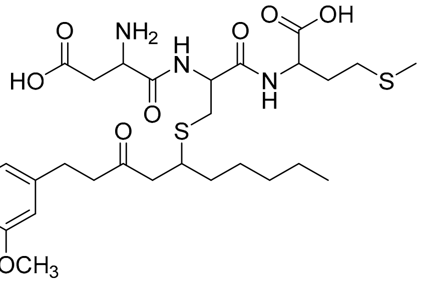

Figure S1. MLY8 (Asp(α)-Cys(6-shogaol)-Met)

Figure S2. Representative semisynthetic route for MLY8:

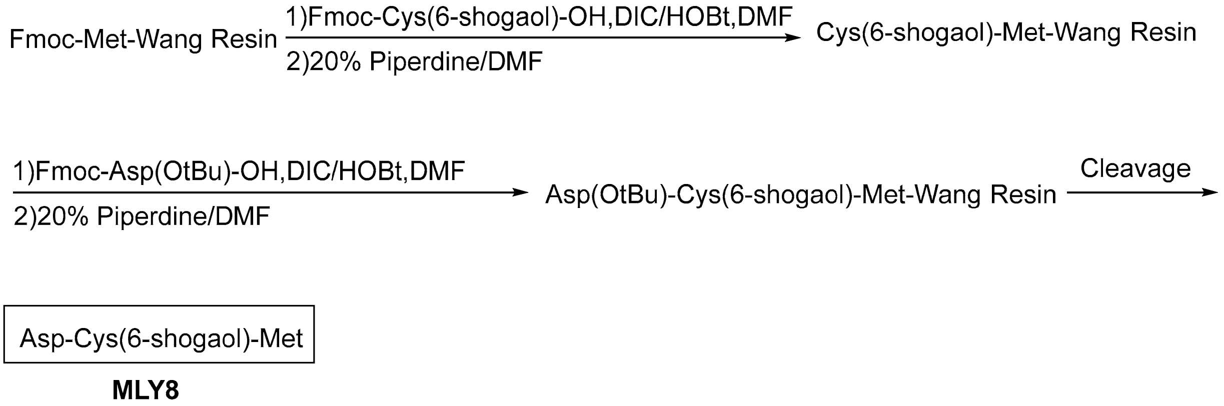

MLY analogs were synthesized by standard Fmoc solid-phase peptide synthesis (SPPS) on Wang resin. Briefly, Fmoc-protected amino acid-loaded Wang resin was swollen in DMF and deprotected using 20% piperidine in DMF. Fmoc-Cys(6-shogaol)-OH was coupled to the resin using DIC/HOBt activation in DMF, followed by Fmoc deprotection. The N-terminal amino acid [e.g., Fmoc-Asp(OtBu)-OH for MLY8] was subsequently coupled under identical conditions, and the Fmoc group was removed. Completion of coupling and deprotection steps was monitored by the Kaiser (ninhydrin) test.

The resin-bound product was cleaved and globally deprotected using a TFA/EDT/TIS/H_2O cocktail (90:5:2.5:2.5, v/v/v/v) at room temperature. The crude product was precipitated with cold methyl tert-butyl ether, collected by filtration, and dried under vacuum.

Crude products were purified by preparative reverse-phase HPLC on a C18 column using a water/acetonitrile gradient containing 0.1% TFA. Fractions containing the desired product were pooled, concentrated, and lyophilized to afford the purified compounds. Final purity (>95%) was confirmed by analytical HPLC, and compound identity was verified by ESI-MS and ^1H NMR spectroscopy for all analogs except MLY18, for which only ESI-MS data were obtained.

Figure S3. MLY8: (Asp(α)-Cys(6-shogaol)-Met). Molecular formula: $C_{29}H_{45}N_3O_9S_2$; molecular weight (MW): 643.81 Da. Representative ESI-MS and 1H NMR spectra of MLY8 are shown below.

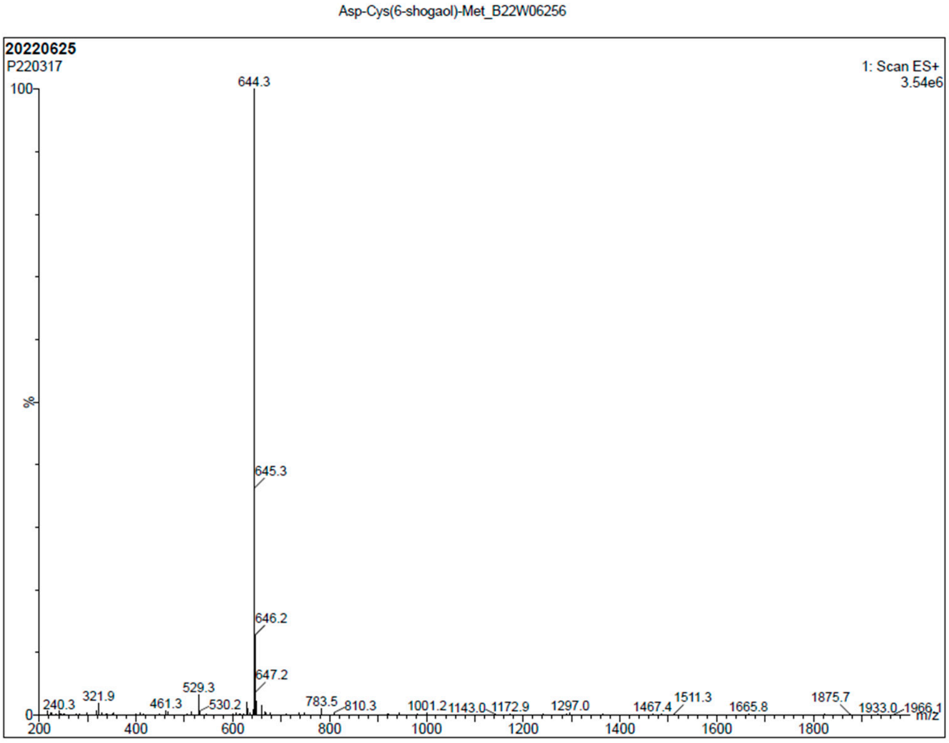

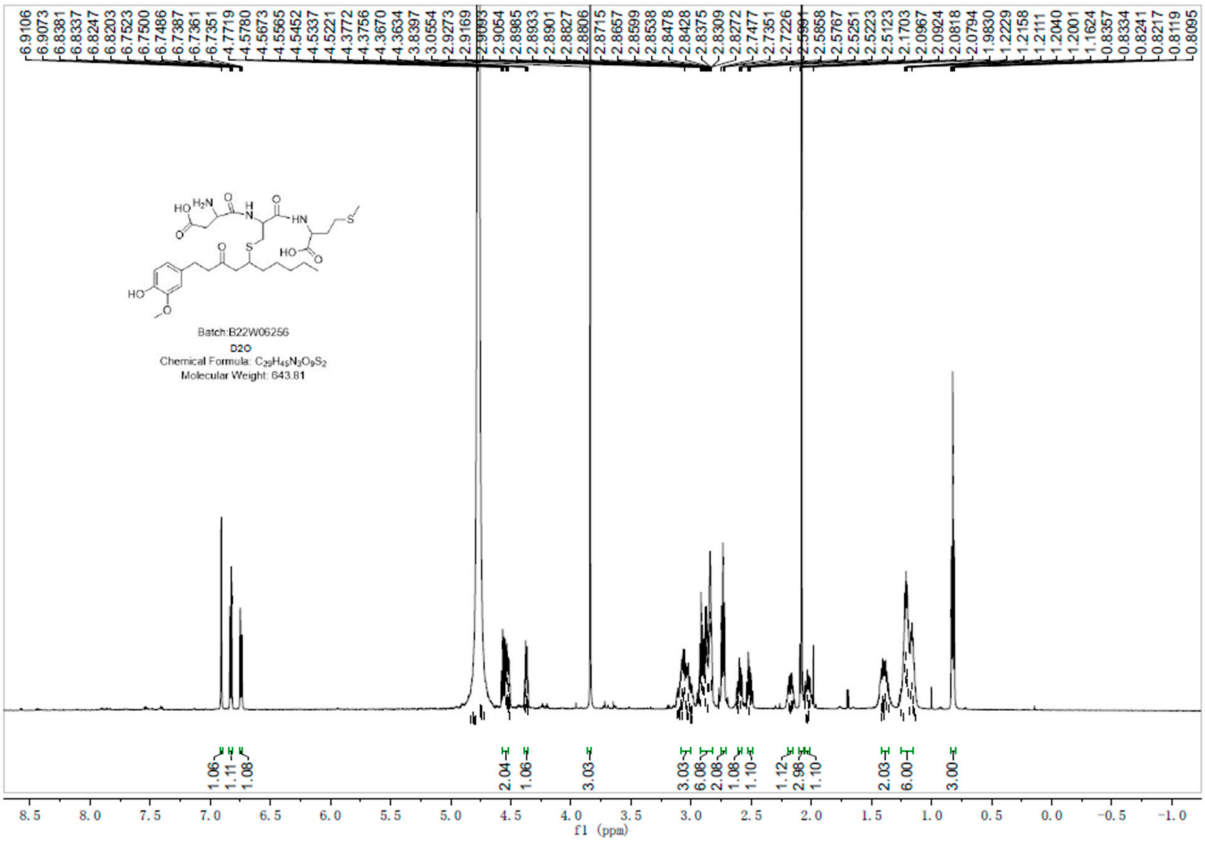

Table S1. Structures and NF-κB inhibitory activities of the 38 MLY analogs

Compound	R1	R2	Structure	NF-κB activity at 20 μM (% of control), mean ± SD
M13	Glu	Gly	Glu(γ)-Cys(6-shogaol)-Gly	78.96 ± 4.04
MLY1	Glu	Ala	Glu(α)-Cys(6-shogaol)-Ala	30.30 ± 11.78
**MLY2**	**Glu**	**Leu**	**Glu(α)-Cys(6-shogaol)-Leu**	**0.56 ± 0.62**
MLY3	Glu	Pro	Glu(α)-Cys(6-shogaol)-Pro	64.80 ± 8.86
MLY4	Glu	Trp	Glu(α)-Cys(6-shogaol)-Trp	80.65 ± 2.22
MLY5	Glu	Tyr	Glu(α)-Cys(6-shogaol)-Tyr	67.18 ± 3.27
MLY6	Asp	Phe	Asp(α)-Cys(6-shogaol)-Phe	50.70 ± 1.38
MLY7	Asp	Leu	Asp(α)-Cys(6-shogaol)-Leu	38.60 ± 3.96
**MLY8**	**Asp**	**Met**	**Asp(α)-Cys(6-shogaol)-Met**	**8.04 ± 4.06**
MLY9	Asp	Pro	Asp(α)-Cys(6-shogaol)-Pro	92.86 ± 2.15
MLY10	Asp	Val	Asp(α)-Cys(6-shogaol)-Val	66.68 ± 11.06
MLY11	Asp	Trp	Asp(α)-Cys(6-shogaol)-Trp	24.75 ± 0.79
MLY12	Asp	Tyr	Asp(α)-Cys(6-shogaol)-Tyr	47.43 ± 5.50
MLY13	Glu	Met	Glu(α)-Cys(6-shogaol)-Met	60.22 ± 13.55
MLY14	Asp	Ile	Asp(α)-Cys(6-shogaol)-Ile	80.67 ± 11.28
MLY15	Glu	Phe	Glu(α)-Cys(6-shogaol)-Phe	47.98 ± 9.35
MLY16	Glu	Ile	Glu(α)-Cys(6-shogaol)-Ile	68.19 ± 4.46
MLY17	Glu	Val	Glu(α)-Cys(6-shogaol)-Val	45.13 ± 1.96
MLY18	Asp	Gly	Asp(α)-Cys(6-shogaol)-Gly	112.31 ± 7.28
MLY19	Asp	Ala	Asp(α)-Cys(6-shogaol)-Ala	67.04 ± 11.04
MLY20	Glu	Ala	Glu(γ)-Cys(6-shogaol)-Ala	49.75 ± 3.12
MLY21	Glu	Phe	Glu(γ)-Cys(6-shogaol)-Phe	75.28 ± 4.06
MLY22	Glu	Ile	Glu(γ)-Cys(6-shogaol)-Ile	83.39 ± 1.89
MLY23	Glu	Leu	Glu(γ)-Cys(6-shogaol)-Leu	61.27 ± 18.09
MLY24	Glu	Met	Glu(γ)-Cys(6-shogaol)-Met	15.92 ± 1.22
MLY25	Glu	Pro	Glu(γ)-Cys(6-shogaol)-Pro	33.82 ± 9.29
MLY26	Glu	Val	Glu(γ)-Cys(6-shogaol)-Val	15.15 ± 4.35
MLY27	Glu	Trp	Glu(γ)-Cys(6-shogaol)-Trp	26.63 ± 11.99
MLY28	Glu	Tyr	Glu(γ)-Cys(6-shogaol)-Tyr	26.25 ± 13.89
MLY29	Asp	Gly	Asp(β)-Cys(6-shogaol)-Gly	18.22 ± 2.59
MLY30	Asp	Ala	Asp(β)-Cys(6-shogaol)-Ala	16.07 ± 4.06
MLY31	Asp	Phe	Asp(β)-Cys(6-shogaol)-Phe	50.52 ± 3.10
MLY32	Asp	Ile	Asp(β)-Cys(6-shogaol)-Ile	40.60 ± 1.19
MLY33	Asp	Leu	Asp(β)-Cys(6-shogaol)-Leu	23.57 ± 6.50
MLY34	Asp	Met	Asp(β)-Cys(6-shogaol)-Met	14.76 ± 2.78
MLY35	Asp	Pro	Asp(β)-Cys(6-shogaol)-Pro	12.85 ± 5.39
MLY36	Asp	Val	Asp(β)-Cys(6-shogaol)-Val	36.83 ± 8.15
MLY37	Asp	Trp	Asp(β)-Cys(6-shogaol)-Trp	31.58 ± 5.42
MLY38	Asp	Tyr	Asp(β)-Cys(6-shogaol)-Tyr	15.98 ± 6.21

Note to Table S1. Compound identity, R1/R2 substitutions (see Figure S4 for the general structure of the MLY series), structures, and corresponding NF-κB activity at 20 μM (% of control; mean ± SD, n = 3) are presented to

enable direct structure–activity comparison. Analogs were considered more active than M13 when the mean ± SD of the analog did not overlap with the mean ± SD of M13; 29 of the 38 analogs met this criterion across the 38-compound library. Lower residual NF-κB activity indicates greater inhibition. Single-concentration screening values are used for SAR analysis.

Structure–activity relationship (SAR) analysis

The 38-compound library was generated by systematically modifying the amino acid residues flanking the common cysteine–6-shogaol core. The library comprised Glu(α)-, Asp(α)-, Glu(γ)-, and Asp(β)-linked series with variation at the terminal amino acid position, including Gly, Ala, Val, Leu, Ile, Met, Pro, Phe, Trp, and Tyr. This design preserved the common M13-derived core while exploring the influence of flanking-residue identity and linkage configuration on NF-κB inhibitory activity.

The NF-κB reporter cell screen revealed substantial activity differences across the series, demonstrating that both terminal amino acid identity and linkage configuration influence biological activity. Within the Glu(α) series, MLY2 [Glu(α)-Cys(6-shogaol)-Leu] showed the lowest residual NF-κB activity (0.56 ± 0.62% of control), whereas the remaining terminal residues gave substantially higher residual activities, ranging from 30.30 ± 11.78% for MLY1 (Ala) to 80.65 ± 2.22% for MLY4 (Trp), including MLY17 (Val; 45.13 ± 1.96%), MLY15 (Phe; 47.98 ± 9.35%), MLY13 (Met; 60.22 ± 13.55%), MLY3 (Pro; 64.80 ± 8.86%), MLY5 (Tyr; 67.18 ± 3.27%), and MLY16 (Ile; 68.19 ± 4.46%). These results indicate that the strong activity of MLY2 cannot be attributed simply to preservation of the common electrophilic core, because modification of the terminal residue within the same Glu(α) framework produced markedly different activities.

Linkage configuration also influenced activity in a residue-dependent manner. For example, the Met-containing analogs differed substantially among Glu(α), Asp(α), Glu(γ), and Asp(β) configurations: MLY13, MLY8, MLY24, and MLY34 showed residual NF-κB activities of 60.22 ± 13.55%, 8.04 ± 4.06%, 15.92 ± 1.22%, and 14.76 ± 2.78% of control, respectively. Similarly, Leu-containing analogs ranged from 0.56 ± 0.62% for MLY2 [Glu(α)] to 61.27 ± 18.09% for MLY23 [Glu(γ)], with 38.60 ± 3.96% for MLY7 [Asp(α)] and 23.57 ± 6.50% for MLY33 [Asp(β)]. Conversely, the effect of changing linkage configuration was not uniform across terminal residues. For example, Val-containing analogs showed the strongest activity in the Glu(γ) series (MLY26, 15.15 ± 4.35%) and the weakest in the Asp(α) series (MLY10, 66.68 ± 11.06%), with intermediate values for MLY17 [Glu(α); 45.13 ± 1.96%] and MLY36 [Asp(β); 36.83 ± 8.15%]. Collectively, these matched comparisons indicate an interdependence between terminal amino acid identity and linkage configuration rather than a uniform effect attributable to either structural variable alone.

Overall, the SAR analysis demonstrates that the tripeptide environment surrounding the cysteine–6-shogaol core is an important determinant of NF-κB inhibitory activity. The pronounced differences among closely related analogs support the use of systematic tripeptide editing as a means of modulating the biological activity of the M13-derived scaffold and provided the basis for advancing MLY2 and MLY8 to concentration–response and subsequent biological studies. Because the library screen was performed at a single concentration, the 20 μM data define SAR based on relative activity at the screening concentration and are not interpreted as quantitative rankings of potency. Quantitative concentration–response analysis was therefore performed separately for the selected leads MLY2 and MLY8.

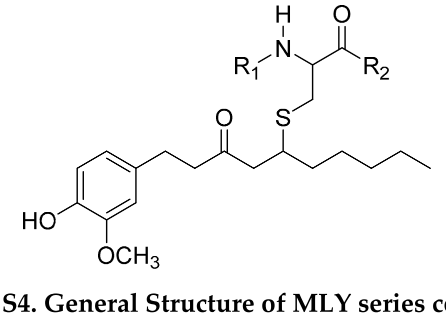

Figure S4. General Structure of MLY series compounds

Figure S5. Characterization of MLY1 (Glu(α)-Cys(6-shogaol)-Ala). Molecular formula: $C_{28}H_{43}N_3O_9S$; molecular weight (MW): 597.72 Da. ESI-MS and 1H NMR spectra are shown below.

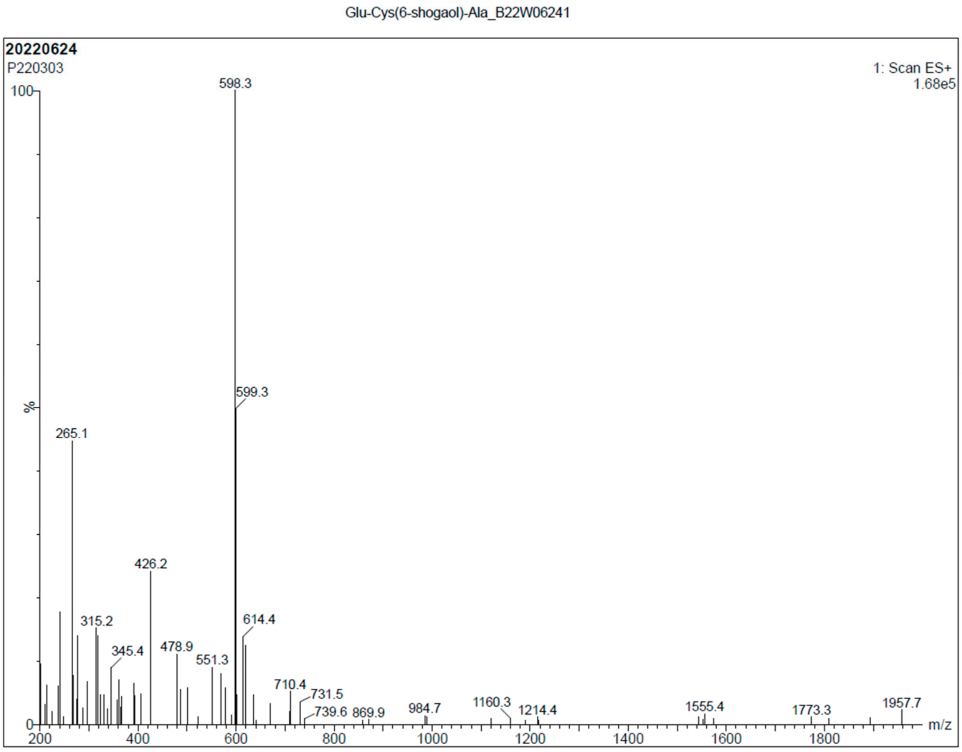

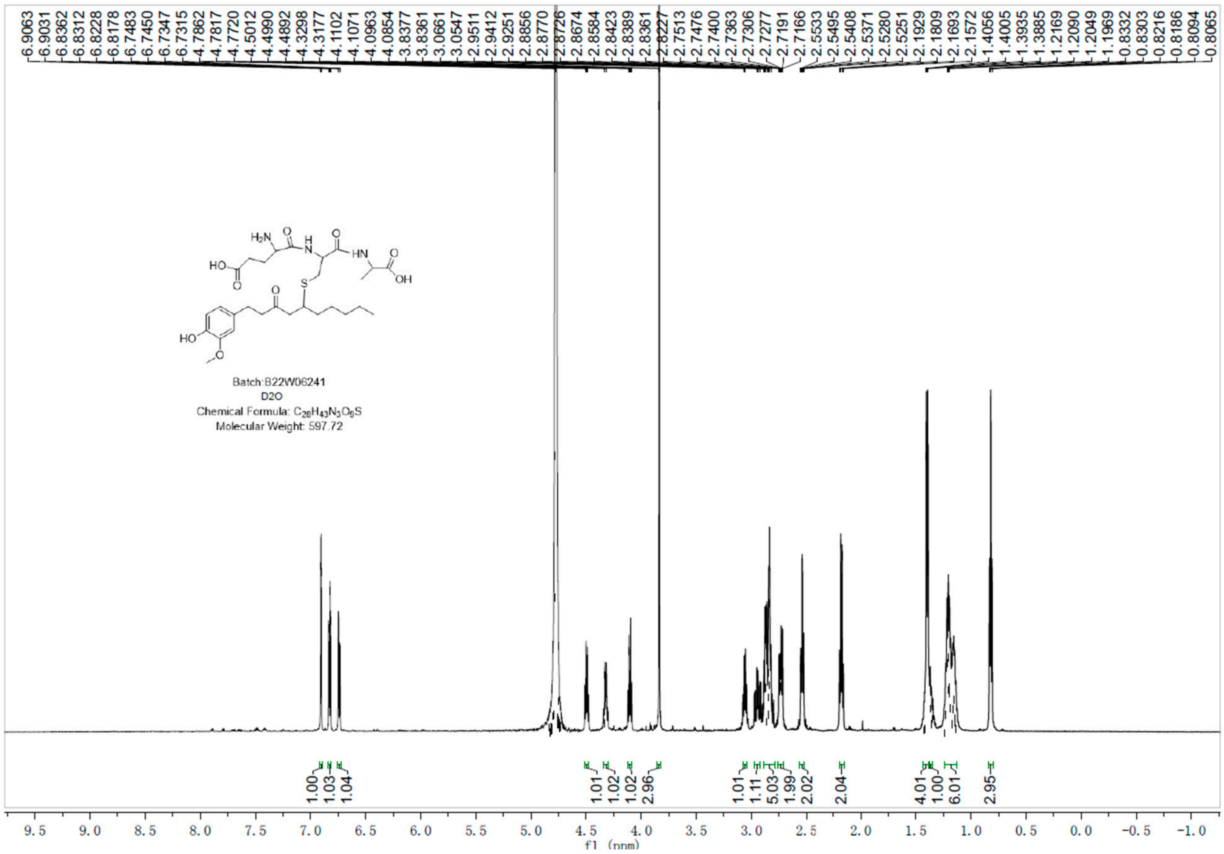

Figure S6. MLY2: (Glu(α)-Cys(6-shogaol)-Leu). Molecular formula: $C_{31}H_{49}N_3O_9S$; molecular weight (MW): 639.81 Da. ESI-MS and 1H NMR spectra are shown below.

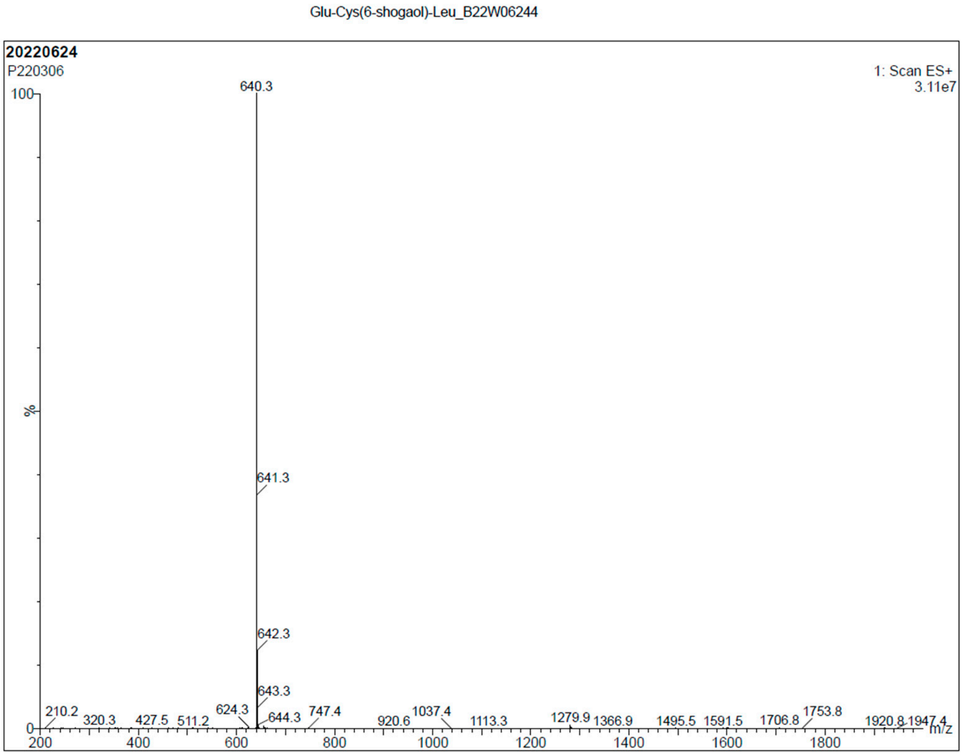

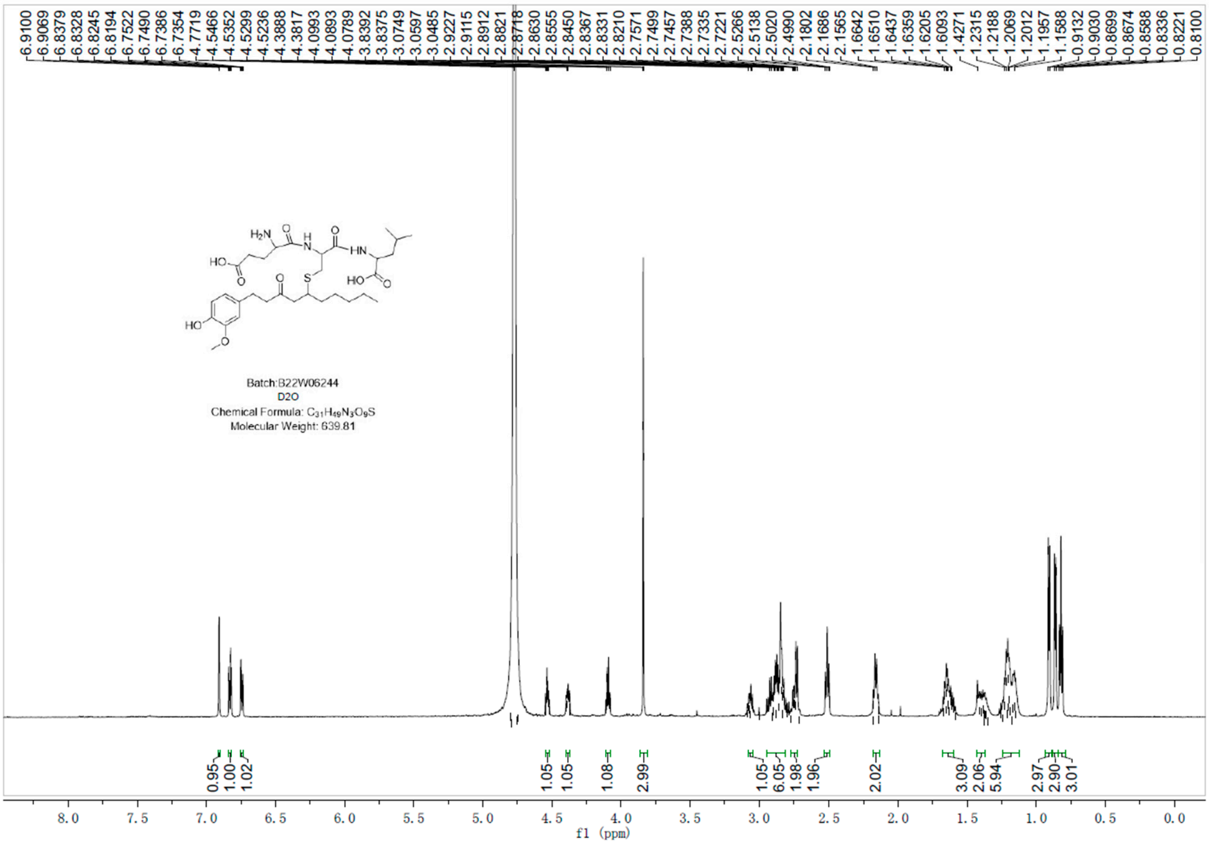

Figure S7. MLY3: (Glu(α)-Cys(6-shogaol)-Pro). Molecular formula: $C_{30}H_{45}N_3O_9S$; molecular weight (MW): 623.76 Da. ESI-MS and 1H NMR spectra are shown below.

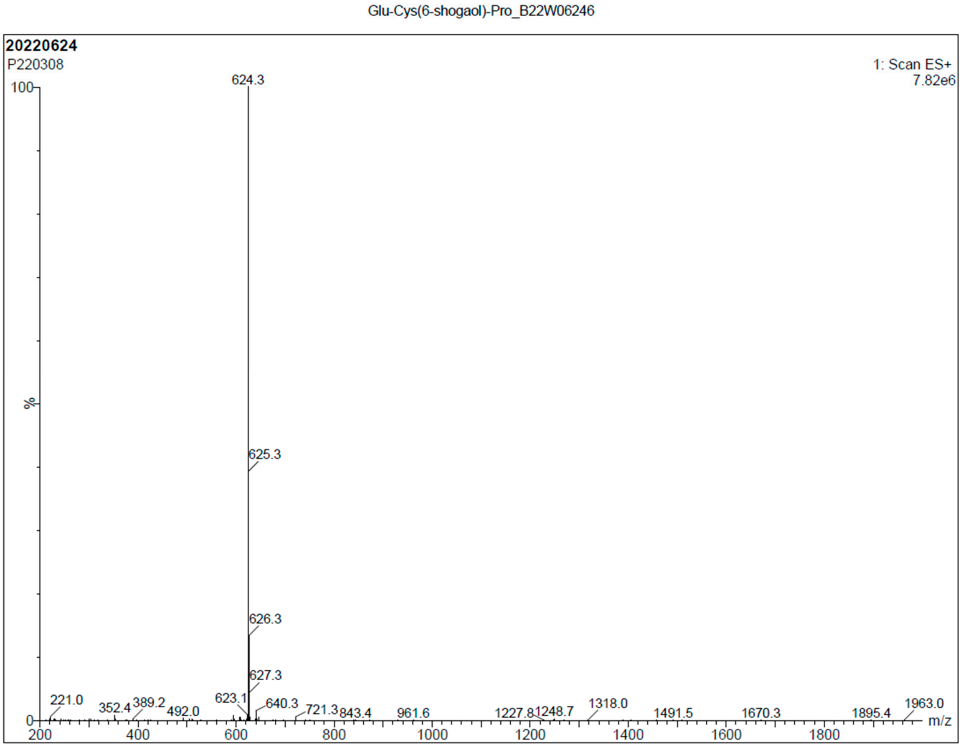

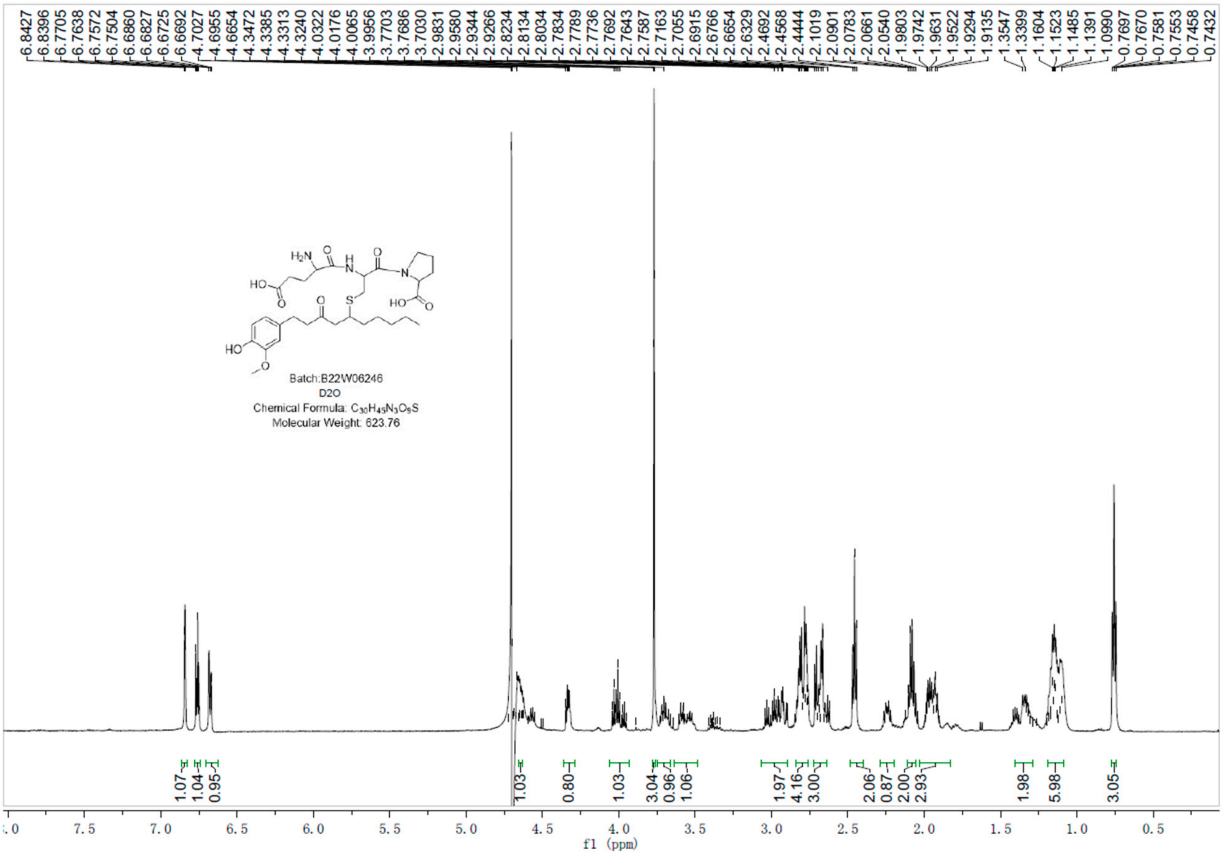

Figure S8. MLY4: (Glu(α)-Cys(6-shogaol)-Trp). Molecular formula: $C_{36}H_{48}N_4O_9S$; molecular weight (MW): 712.86 Da. ESI-MS and 1H NMR spectra are shown below.

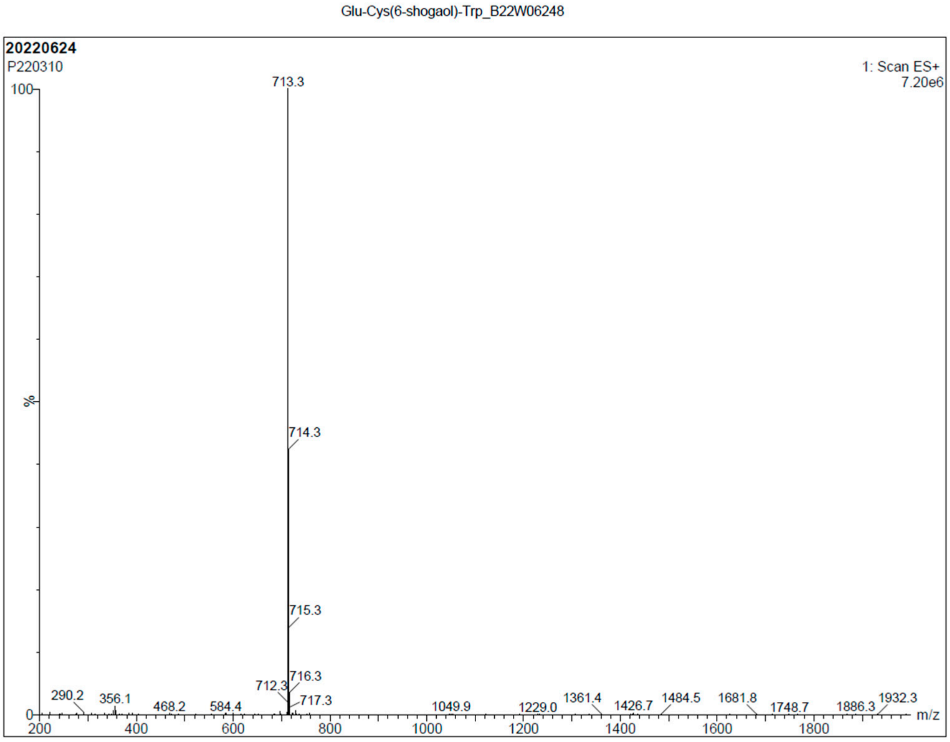

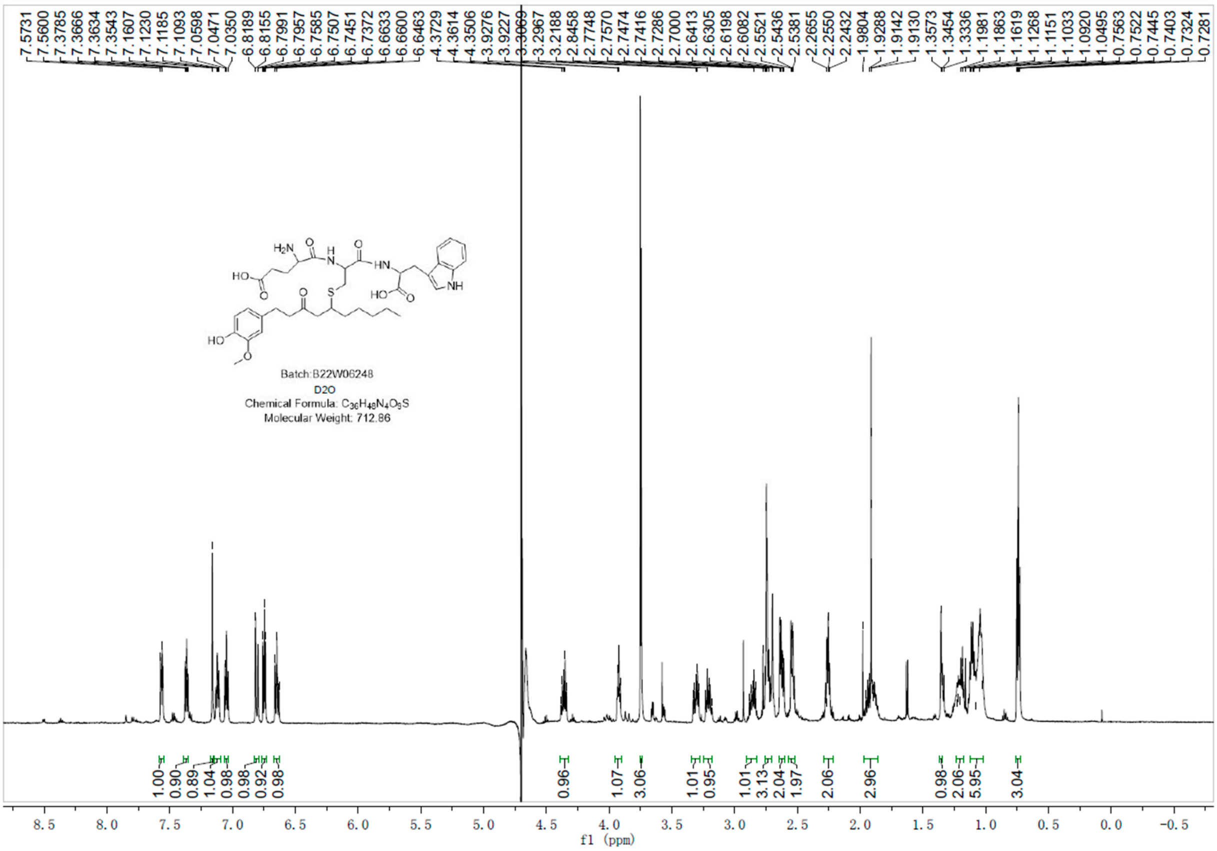

Figure S9. MLY5: (Glu(α)-Cys(6-shogaol)-Tyr). Molecular formula: C34H47N3O10S; molecular weight (MW): 689.82 Da. ESI-MS and 1H NMR spectra are shown below.

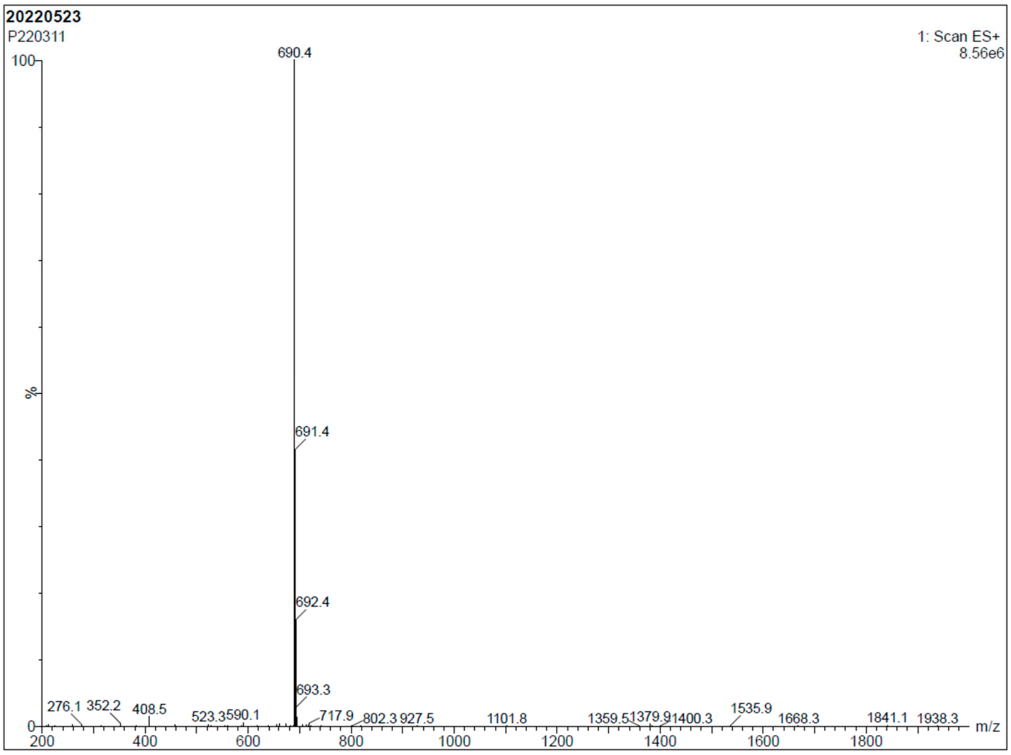

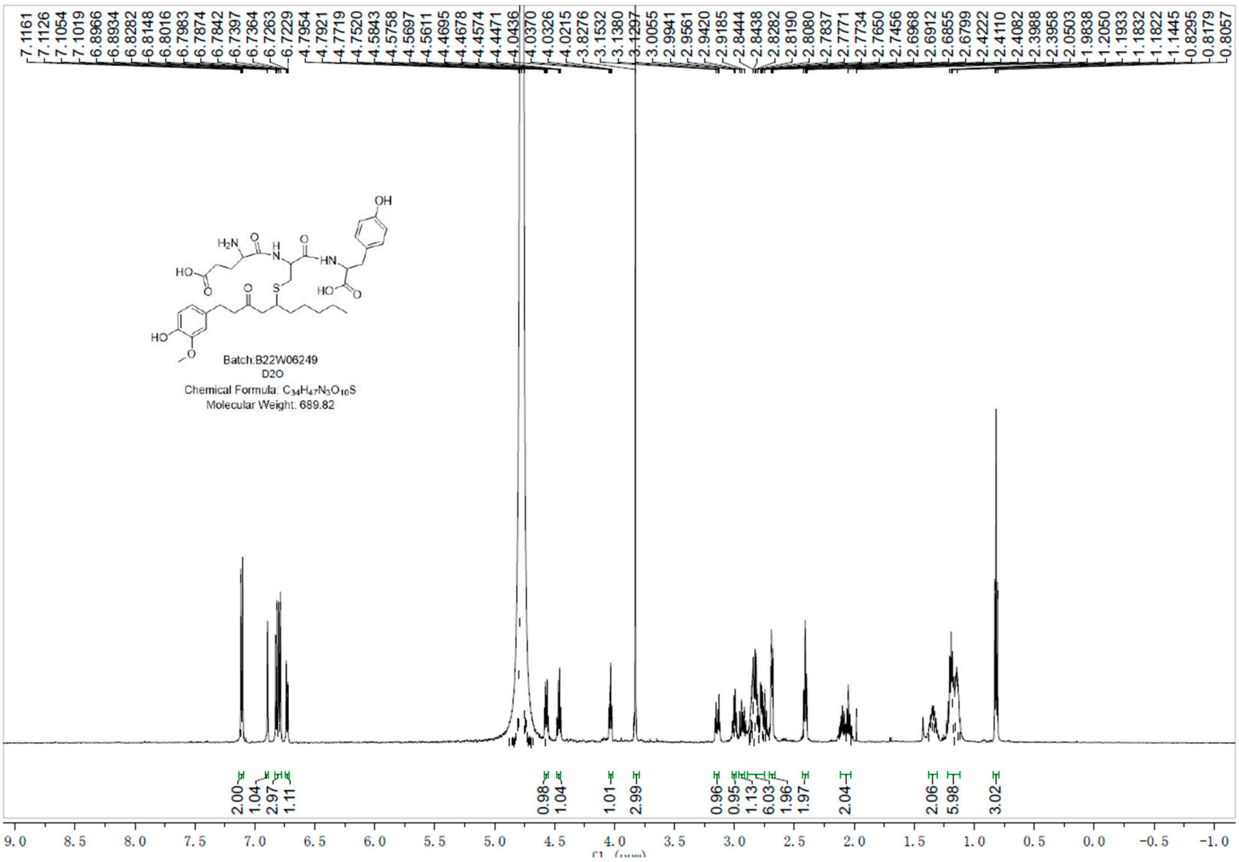

Figure S10. MLY6: (Asp(α)-Cys(6-shogaol)-Phe). Molecular formula: $C_{33}H_{45}N_3O_9S$; molecular weight (MW): 659.80 Da. ESI-MS and 1H NMR spectra are shown below.

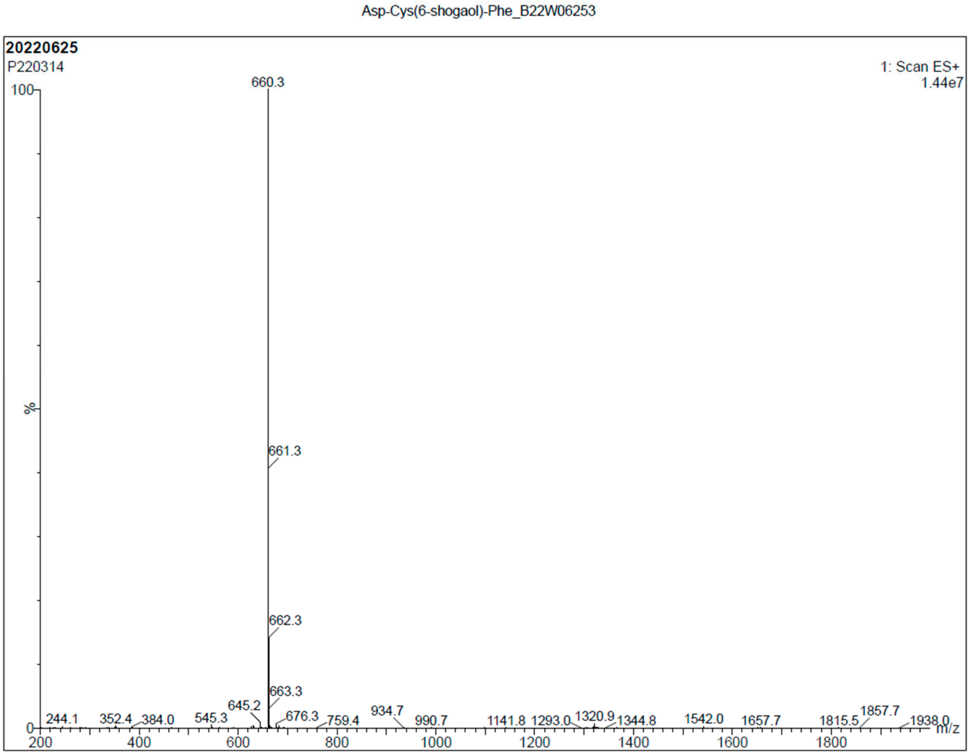

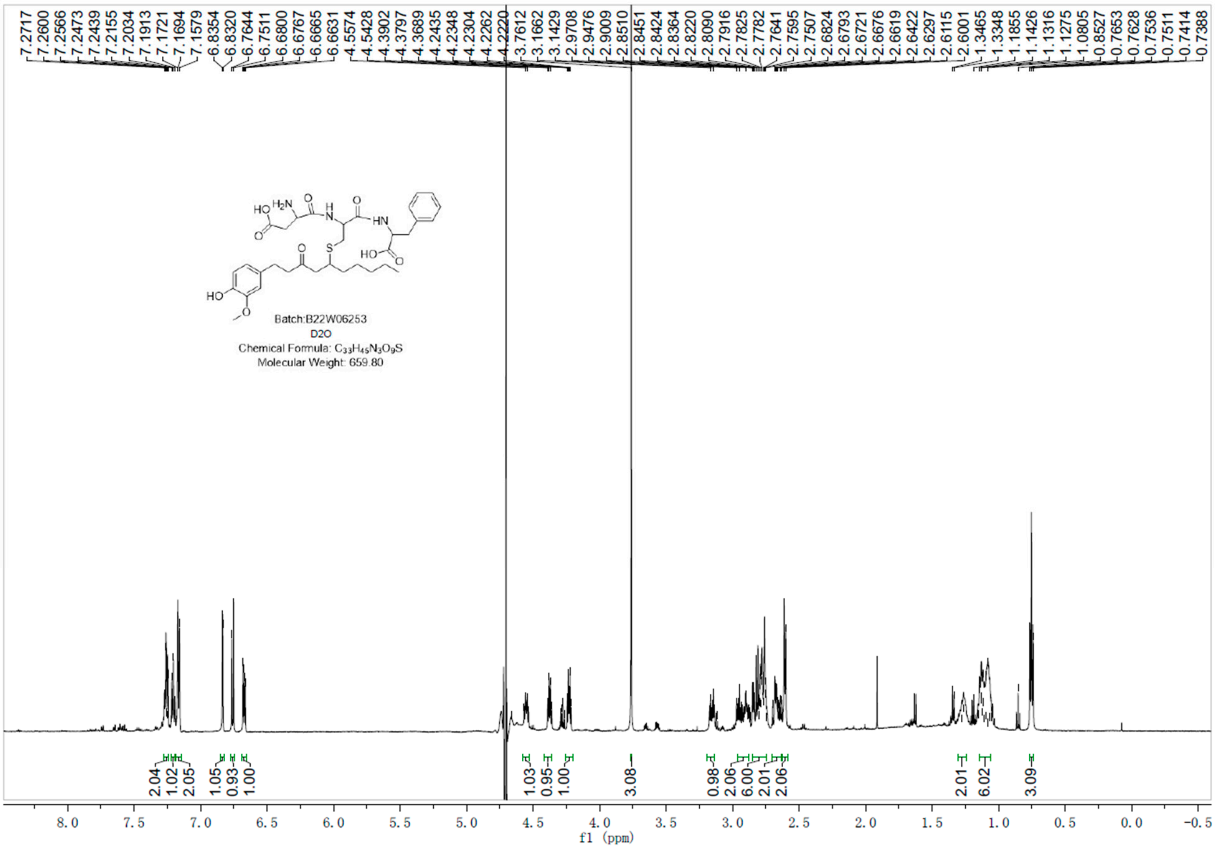

Figure S11. MLY7: (Asp(α)-Cys(6-shogaol)-Leu). Molecular formula: $C_{30}H_{47}N_3O_9S$; molecular weight (MW): 625.78 Da. ESI-MS and 1H NMR spectra are shown below.

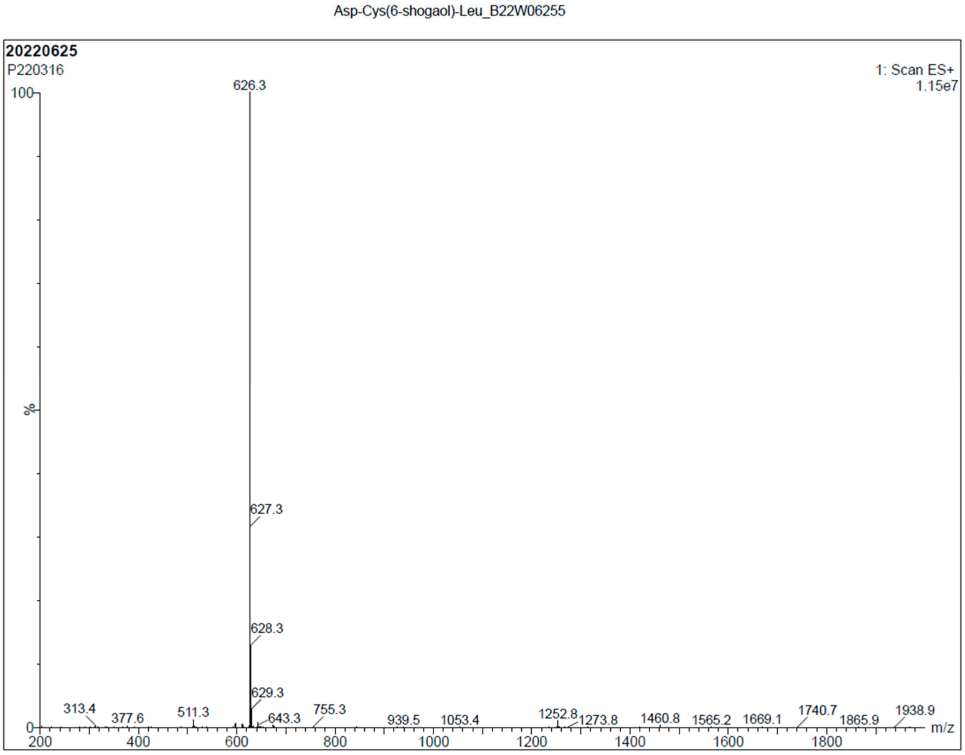

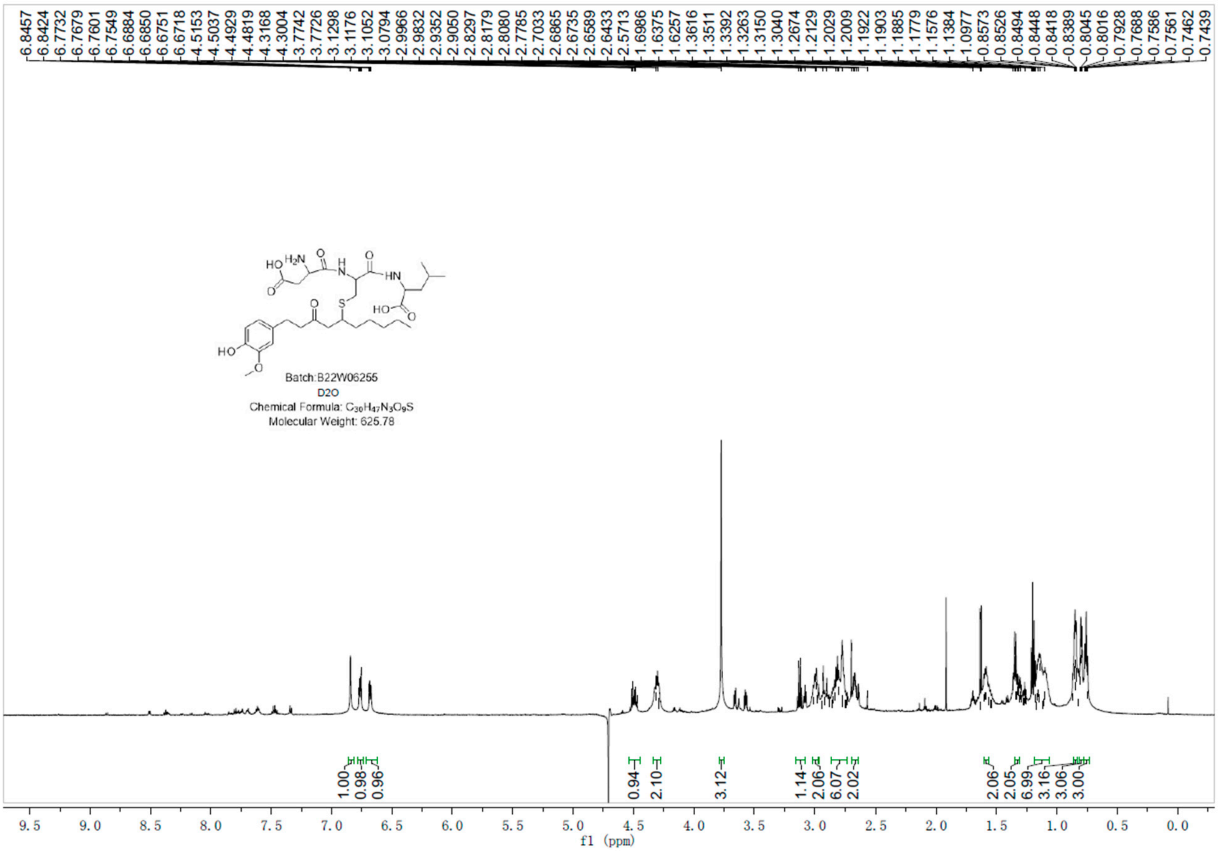

Figure S12. MLY9: (Asp(α)-Cys(6-shogaol)-Pro). Molecular formula: $C_{29}H_{43}N_3O_9S$; molecular weight (MW): 609.74 Da. ESI-MS and 1H NMR spectra are shown below.

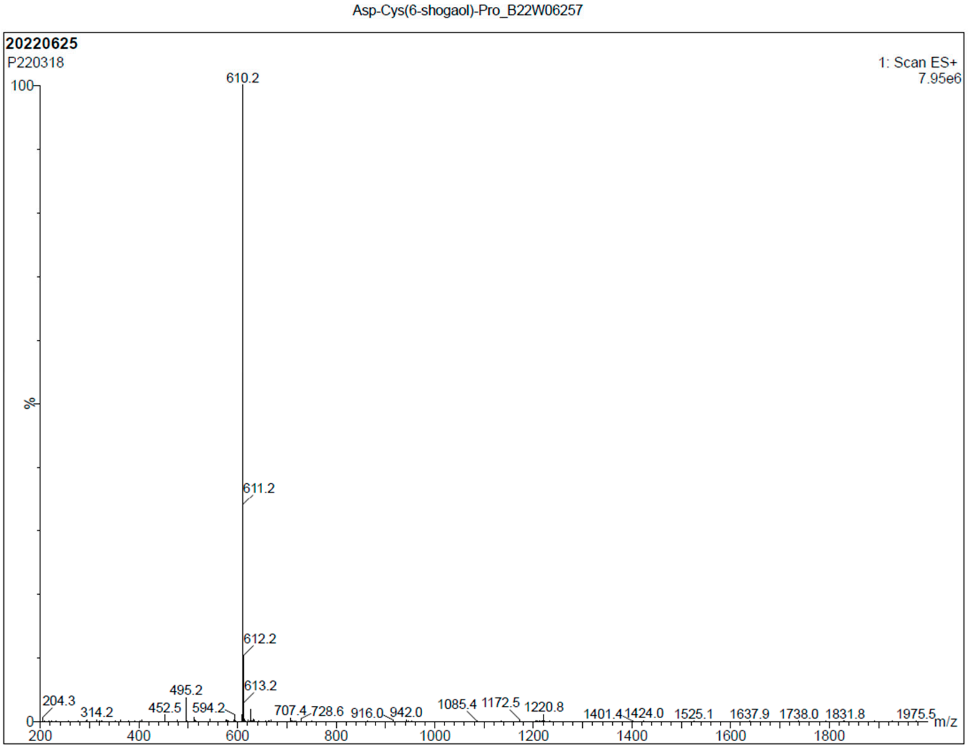

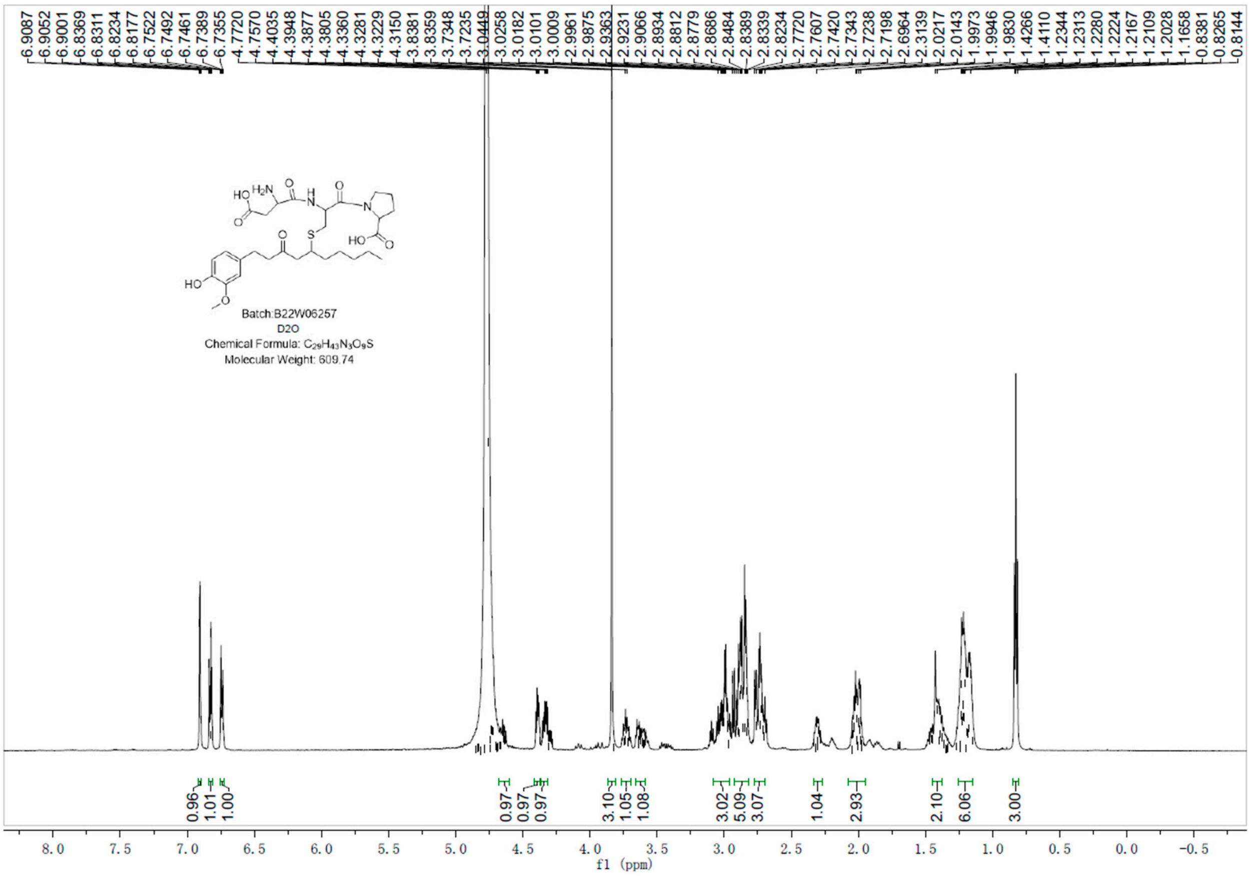

Figure S13. MLY10: (Asp(α)-Cys(6-shogaol)-Val). Molecular formula: $C_{29}H_{45}N_3O_9S$; molecular weight (MW): 611.75 Da. ESI-MS and 1H NMR spectra are shown below.

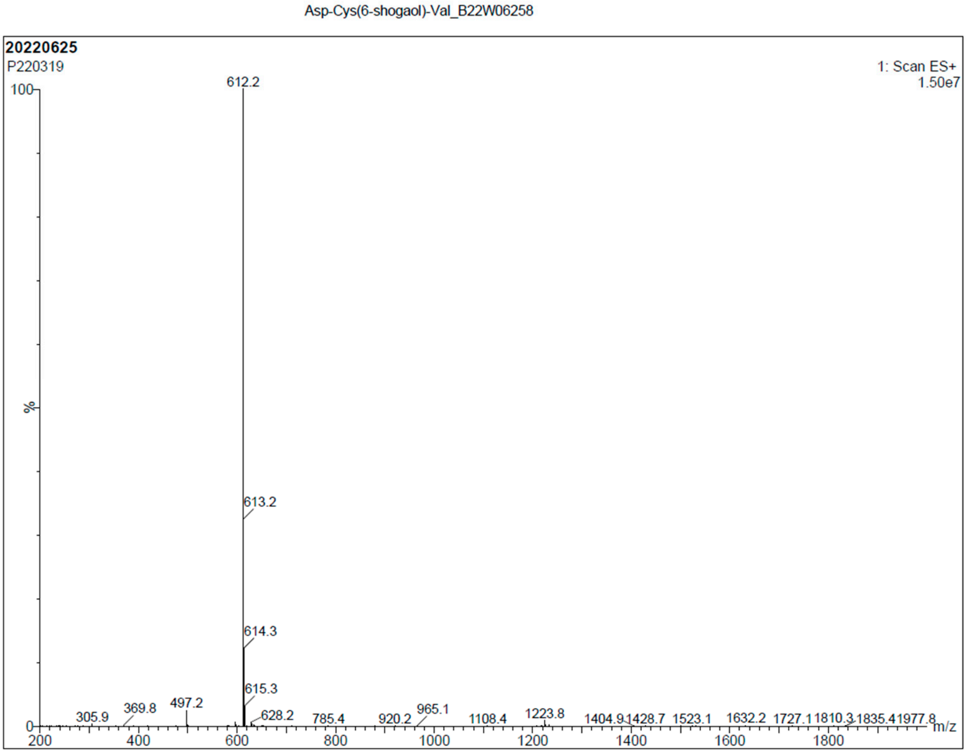

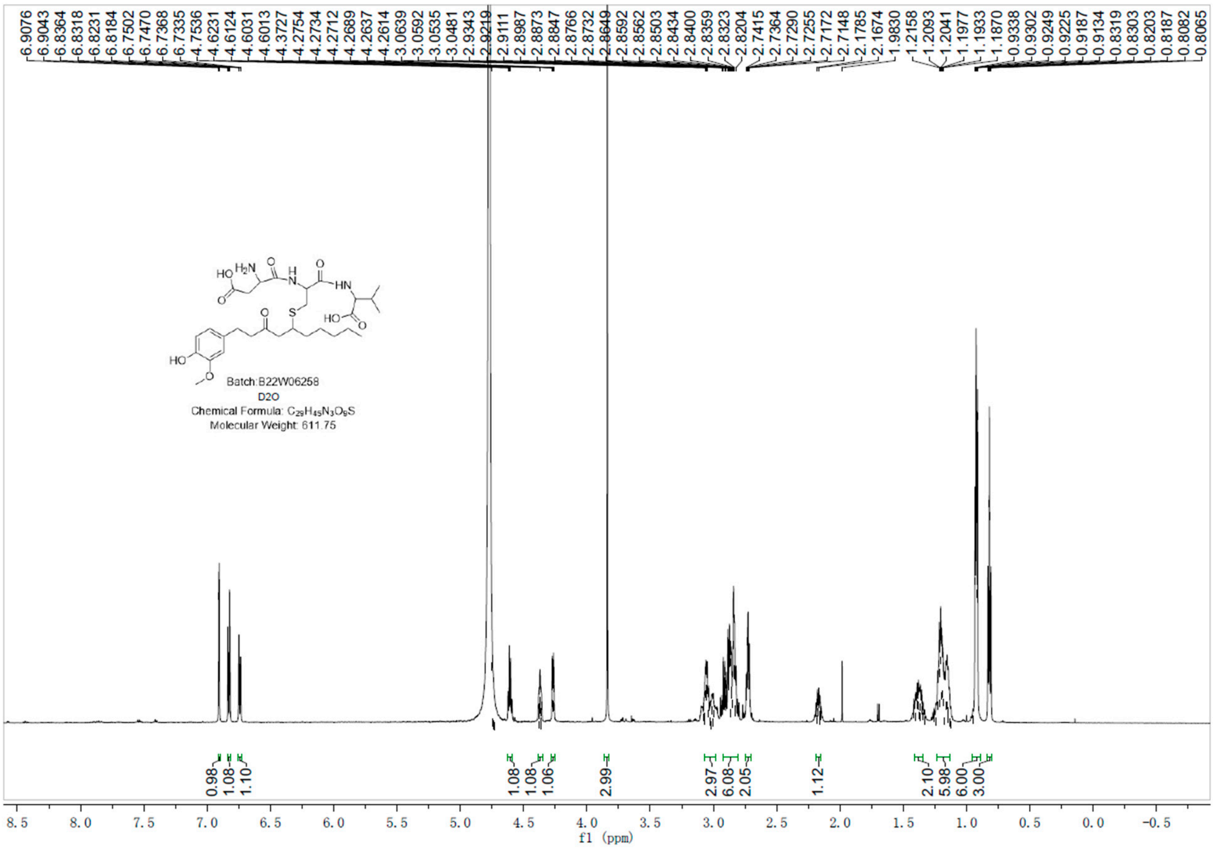

Figure S14. MLY11: (Asp(α)-Cys(6-shogaol)-Trp). Molecular formula: C35H46N4O9S; molecular weight (MW): 698.83 Da. ESI-MS and 1H NMR spectra are shown below.

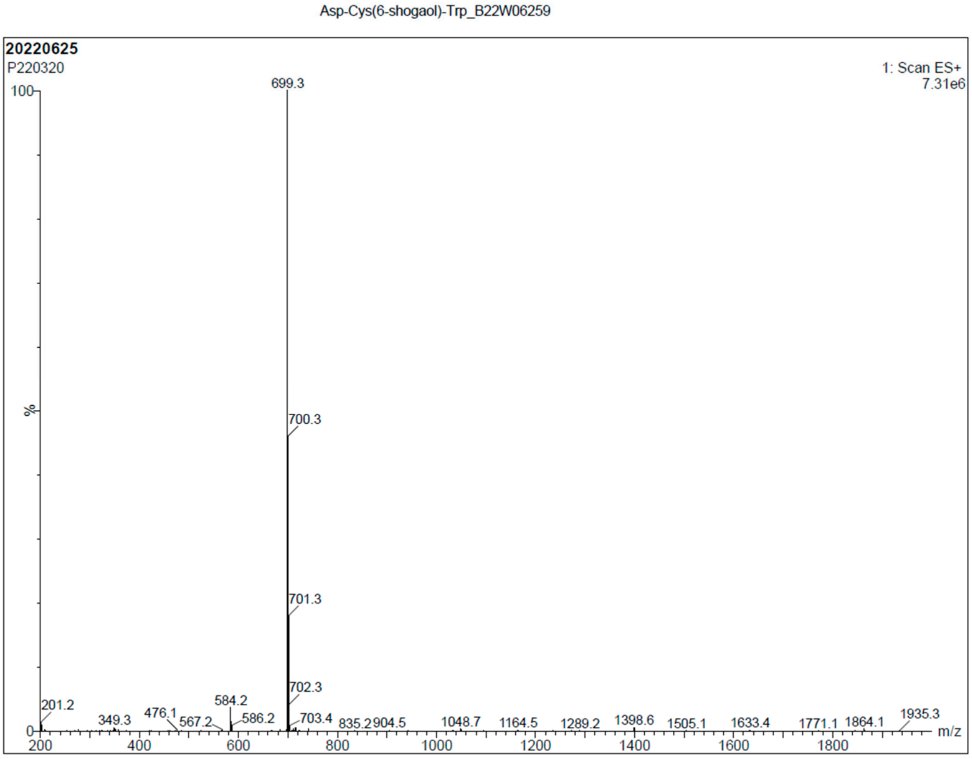

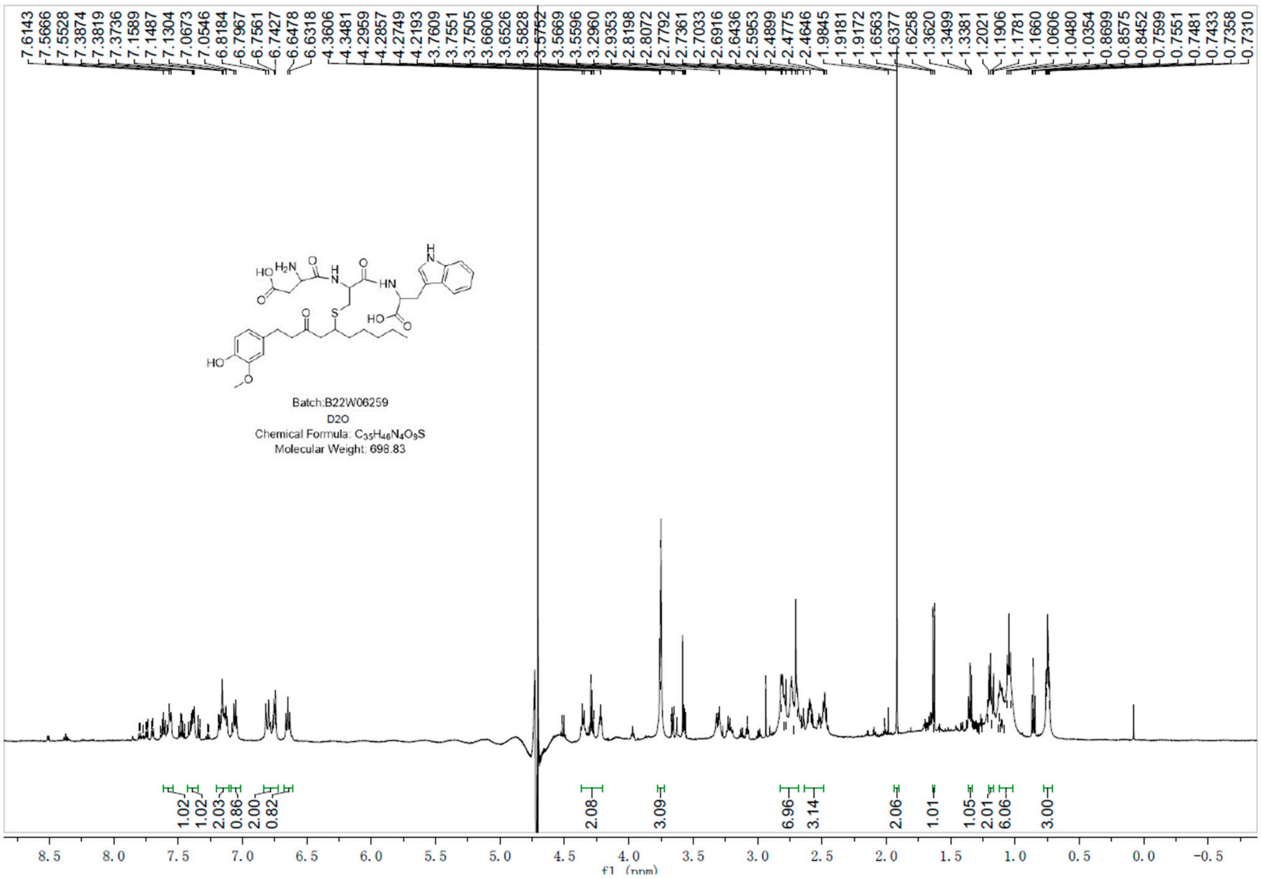

Figure S15. MLY12: (Asp(α)-Cys(6-shogaol)-Tyr). Molecular formula: $C_{33}H_{45}N_3O_{10}S$; molecular weight (MW): 675.79 Da. ESI-MS and 1H NMR spectra are shown below.

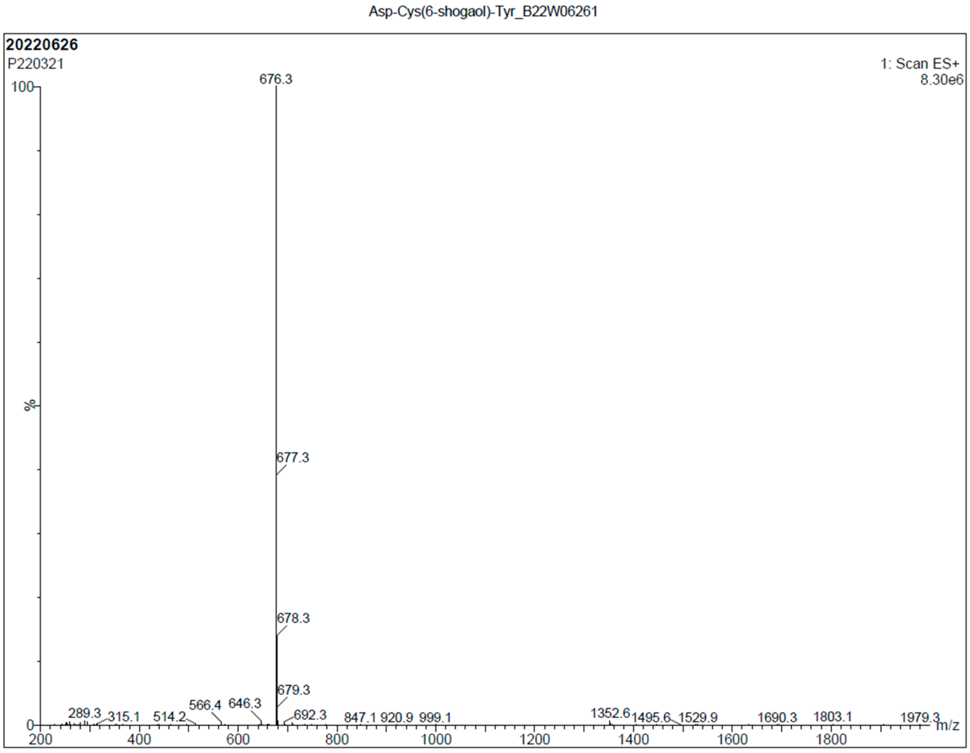

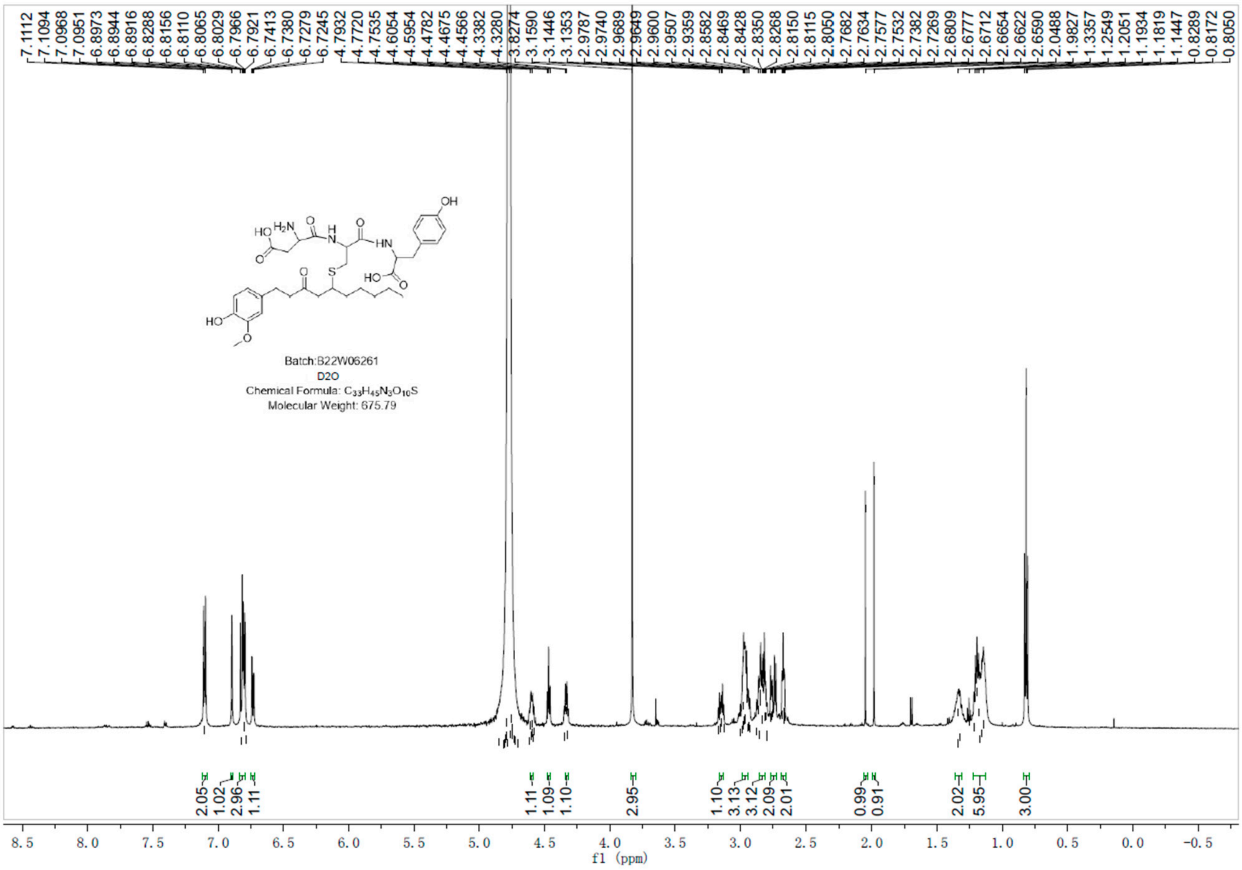

Figure S16. MLY13: (Glu(α)-Cys(6-shogaol)-Met). Molecular formula: $C_{30}H_{47}N_3O_9S_2$; molecular weight (MW): 657.84 Da. ESI-MS and 1H NMR spectra are shown below.

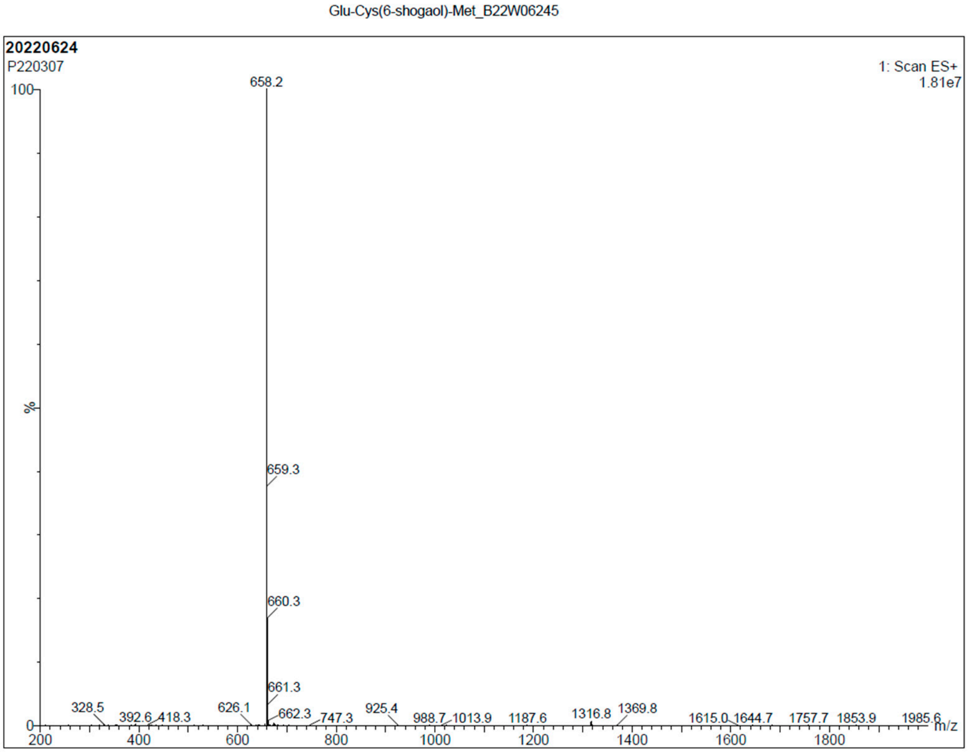

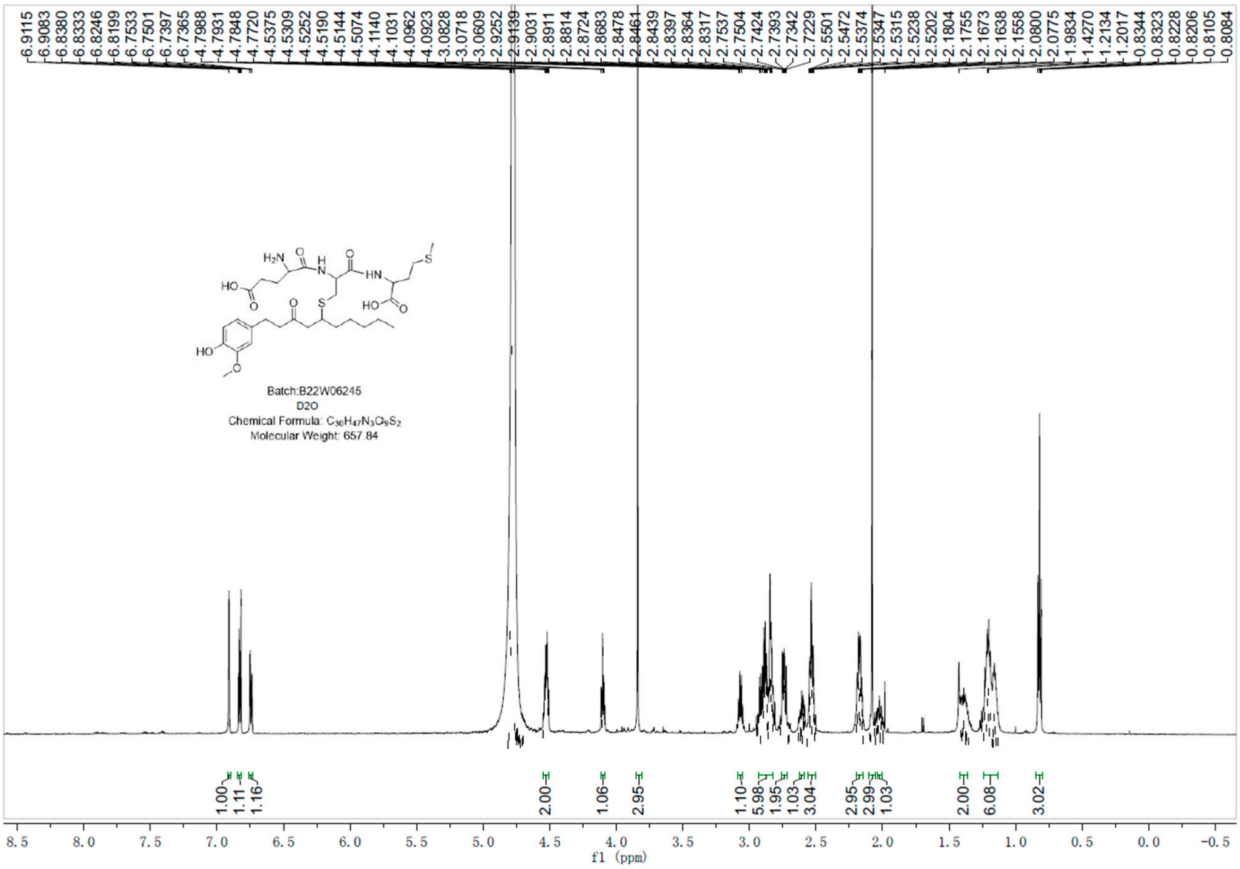

Figure S17. MLY14: (Asp(α)-Cys(6-shogaol)-Ile). Molecular formula: $C_{30}H_{47}N_3O_9S$; molecular weight (MW): 625.78 Da. ESI-MS and 1H NMR spectra are shown below.

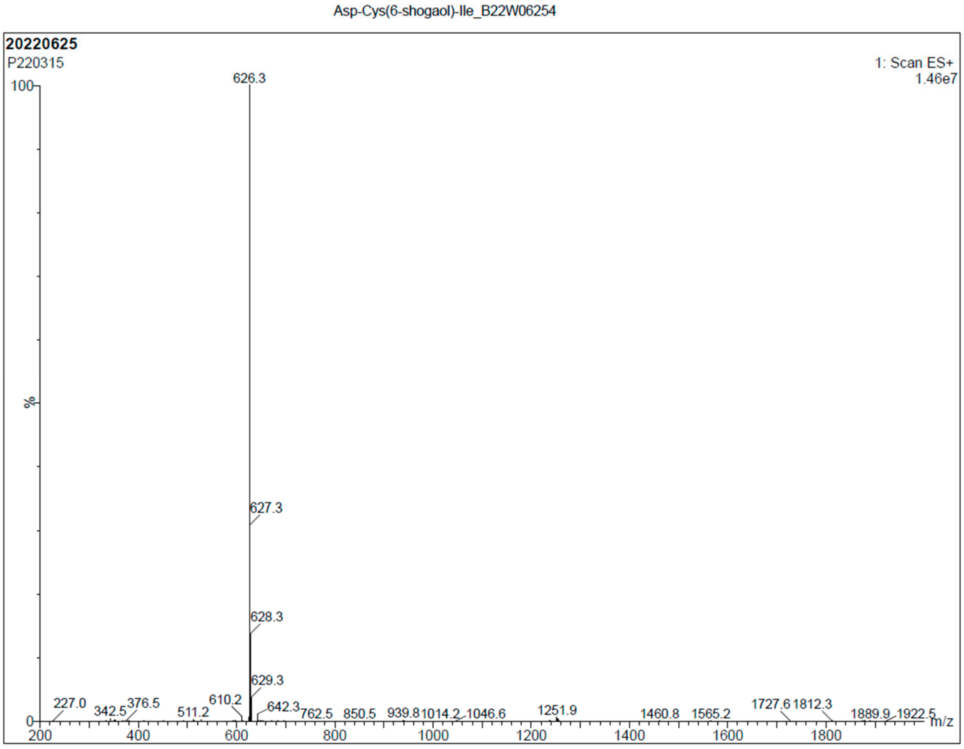

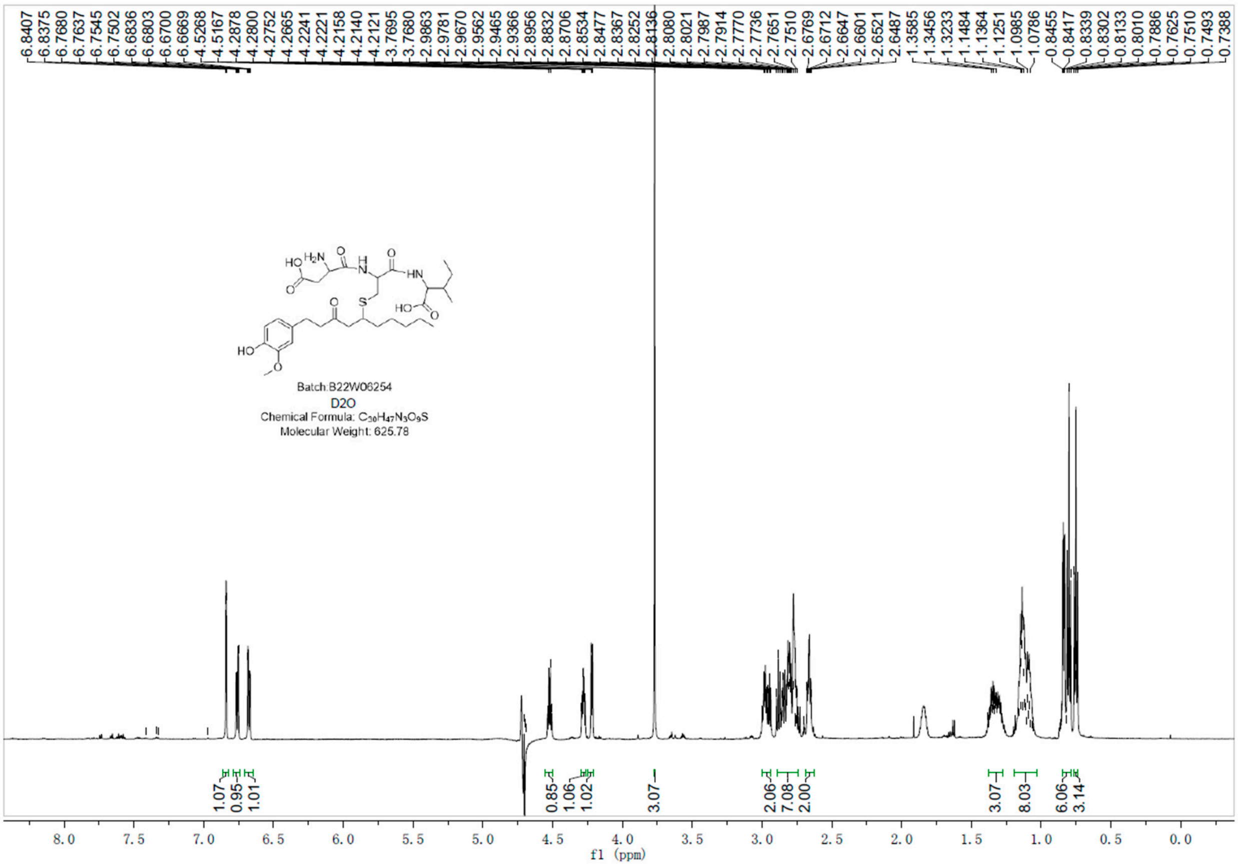

Figure S18. MLY15: (Glu(α)-Cys(6-shogaol)-Phe). Molecular formula: $C_{34}H_{47}N_3O_9S$; molecular weight (MW): 673.82 Da. ESI-MS and 1H NMR spectra are shown below.

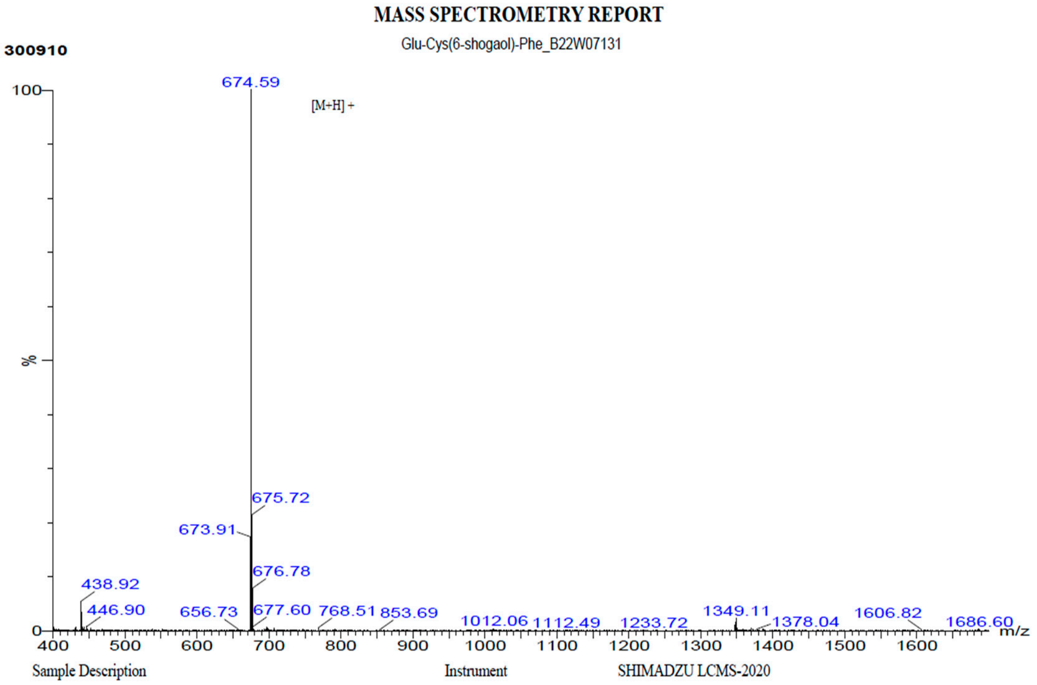

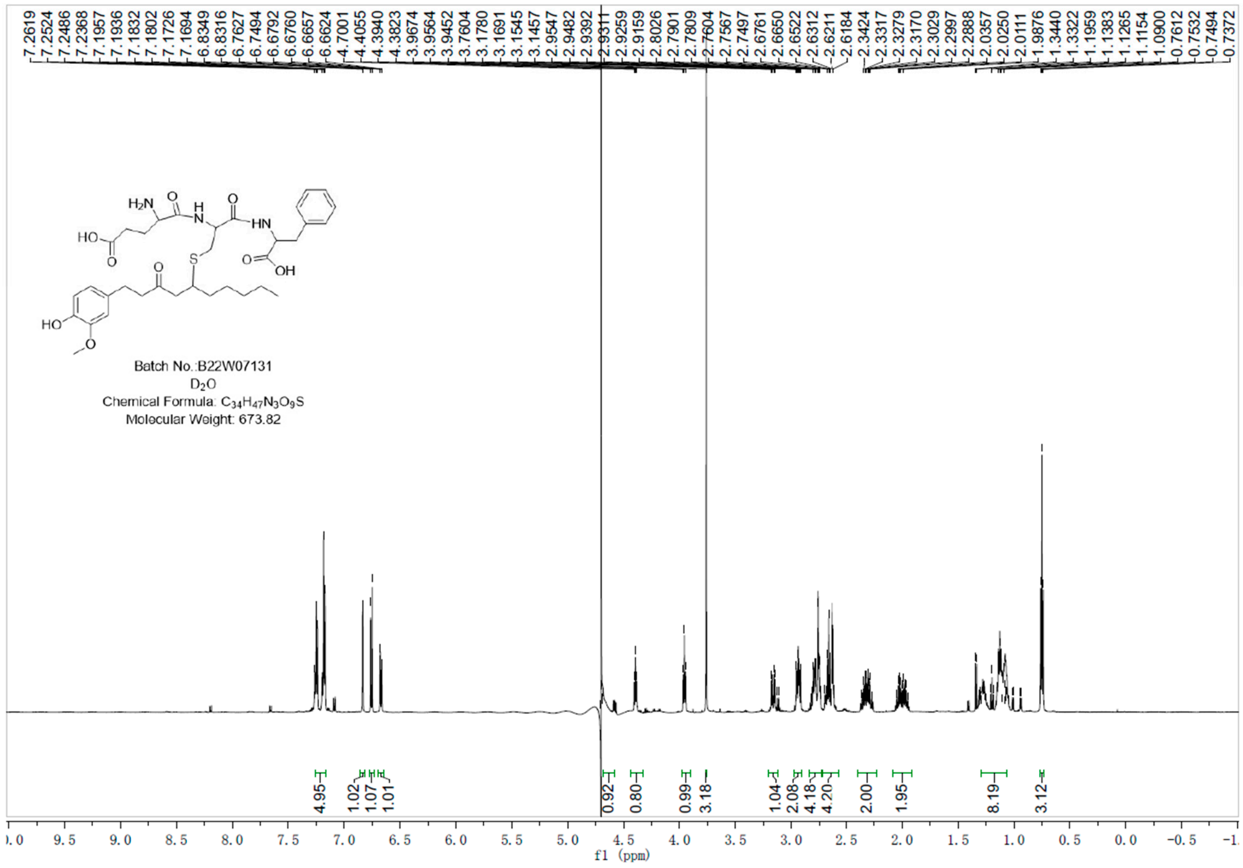

Figure S19. MLY16: (Glu(α)-Cys(6-shogaol)-Ile). Molecular formula: $C_{31}H_{49}N_3O_9S$; molecular weight (MW): 639.81 Da. ESI-MS and 1H NMR spectra are shown below.

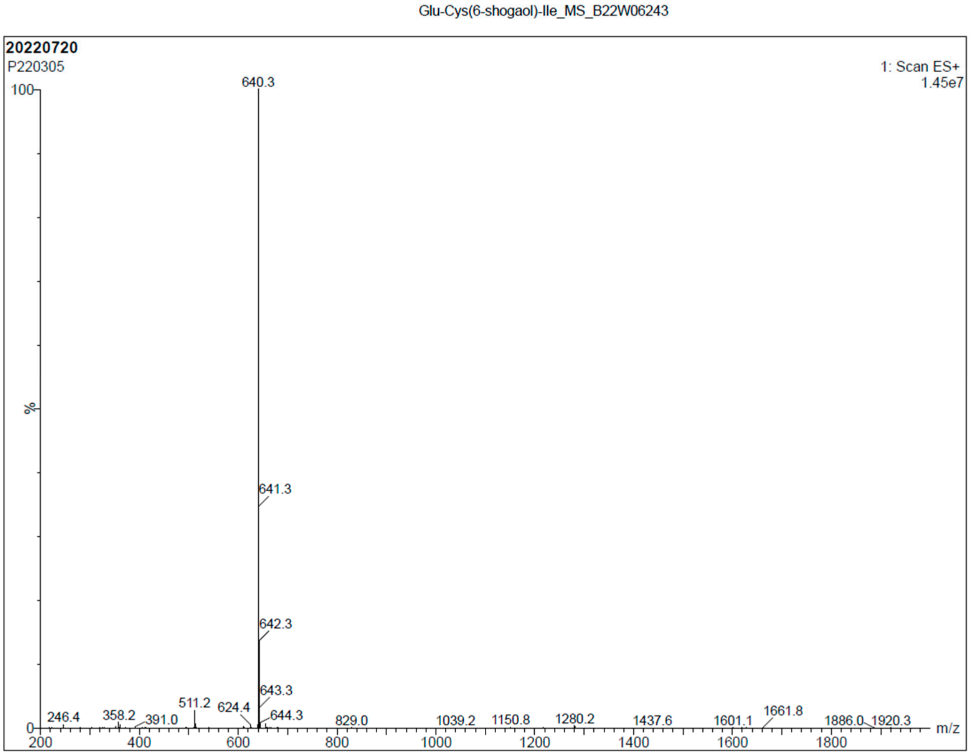

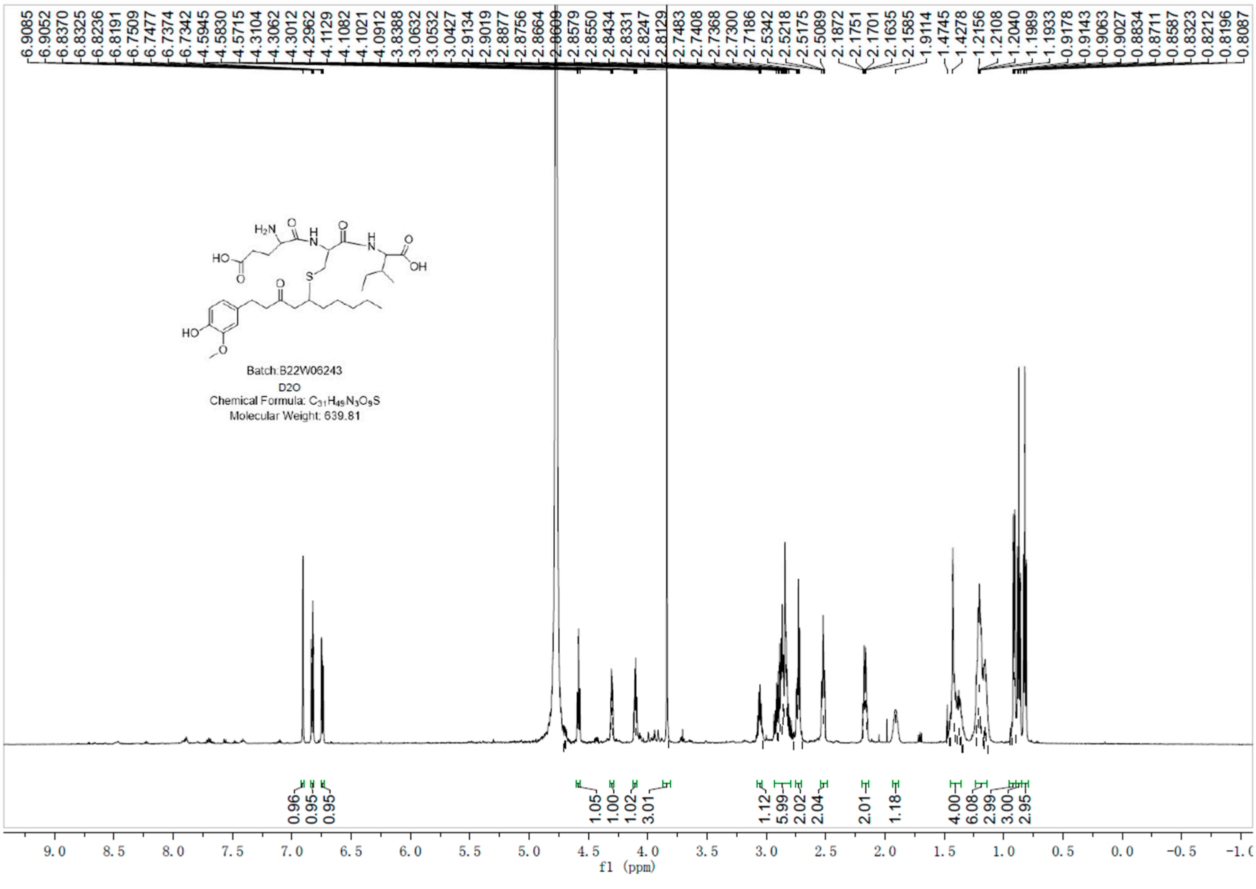

Figure S20. MLY17: (Glu(α)-Cys(6-shogaol)-Val). Molecular formula: $C_{30}H_{47}N_3O_9S$; molecular weight (MW): 625.78 Da. ESI-MS and 1H NMR spectra are shown below.

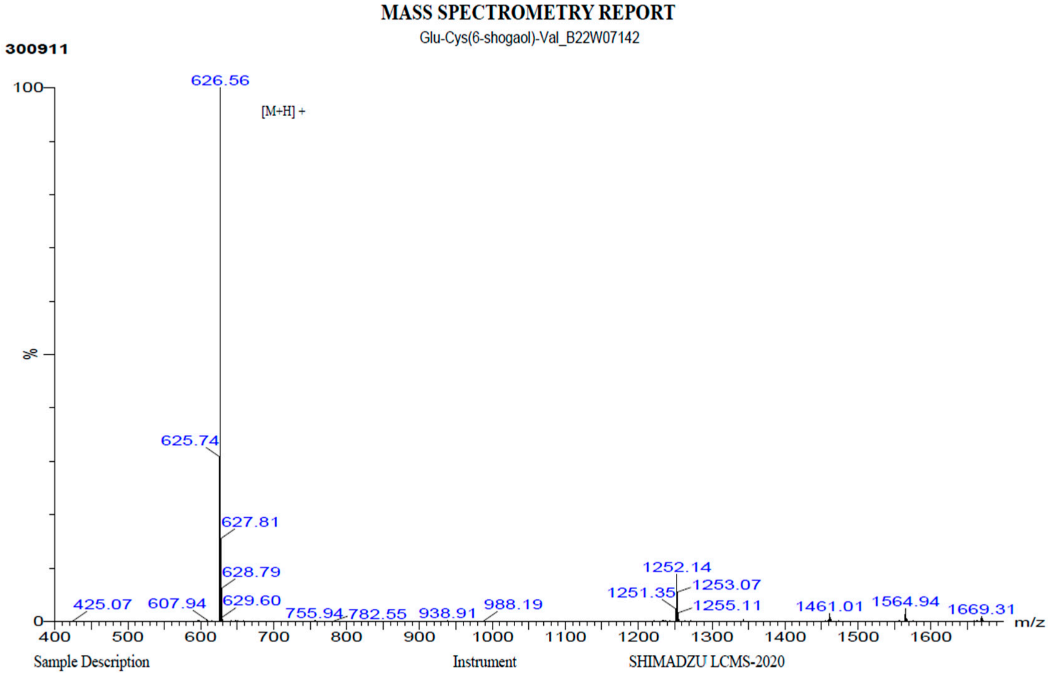

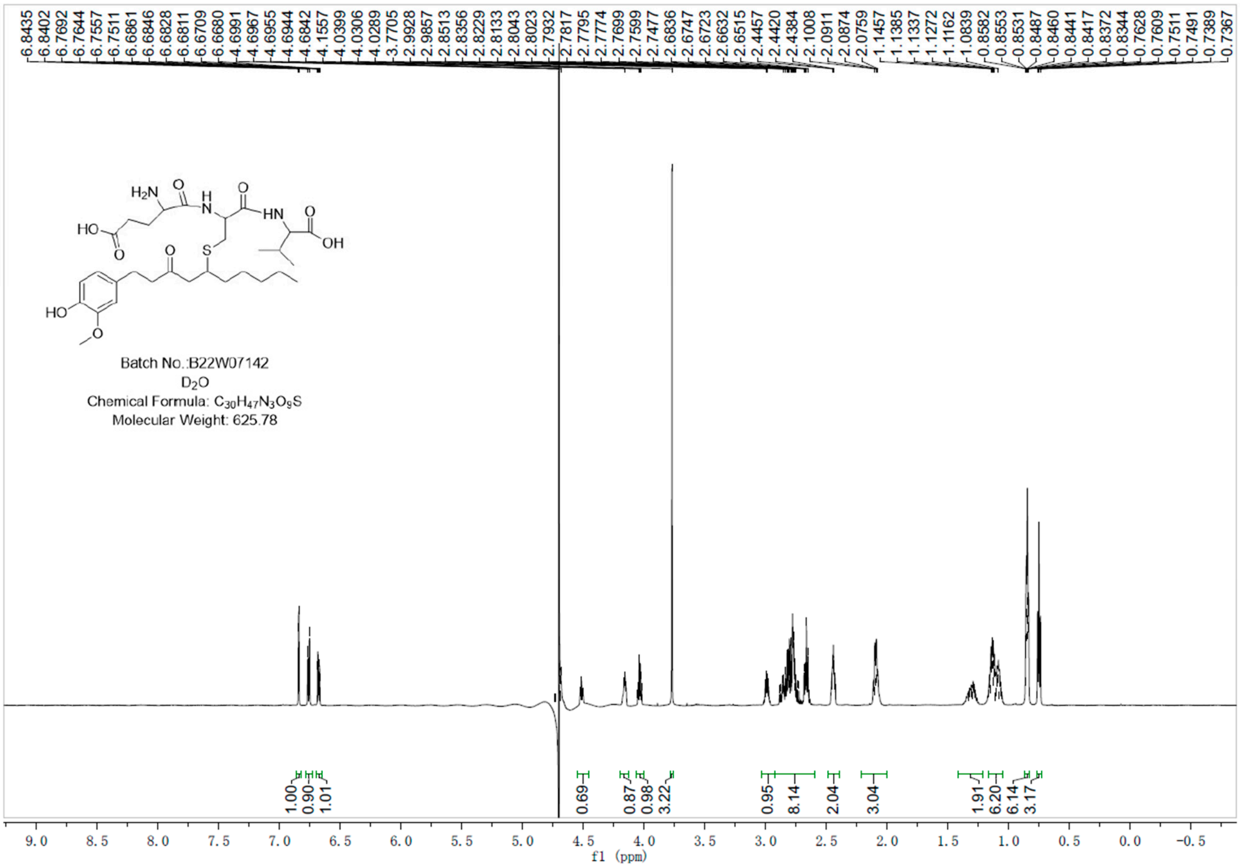

Figure S21. MLY18: (Asp(α)-Cys(6-shogaol)-Gly). Molecular formula: $C_{26}H_{39}N_3O_9S$; molecular weight (MW): 569.67 Da. ESI-MS spectrum is shown below. The ^{1}H NMR spectrum is unavailable due to a technical issue.

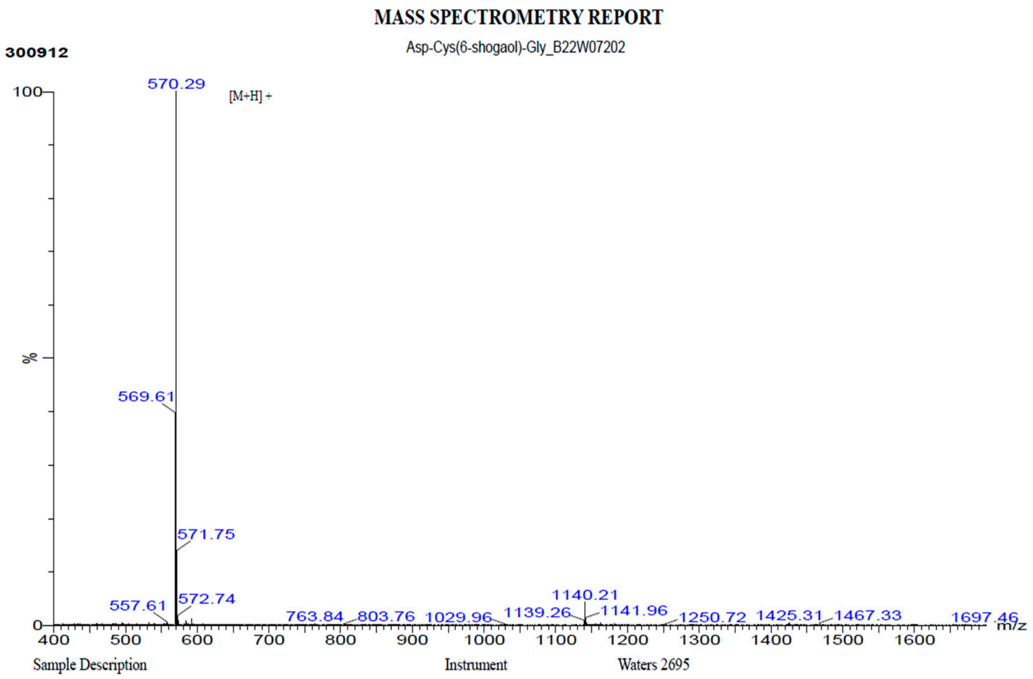

Figure S22. MLY19: (Asp(α)-Cys(6-shogaol)-Ala). Molecular formula: $C_{27}H_{41}N_3O_9S$; molecular weight (MW): 583.70 Da. ESI-MS and 1H NMR spectra are shown below.

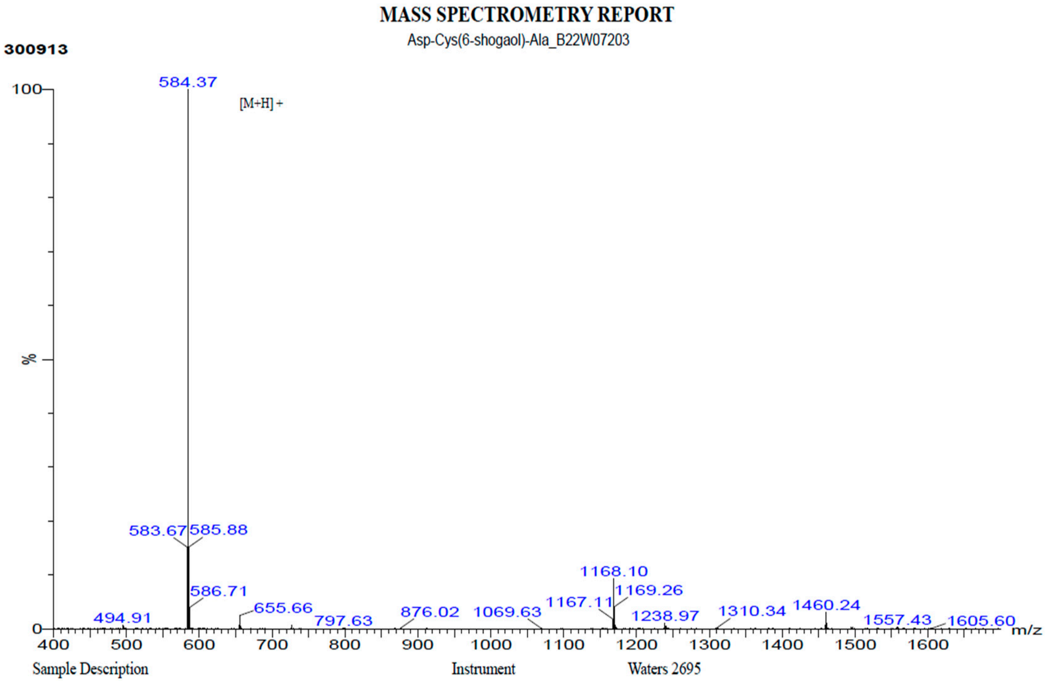

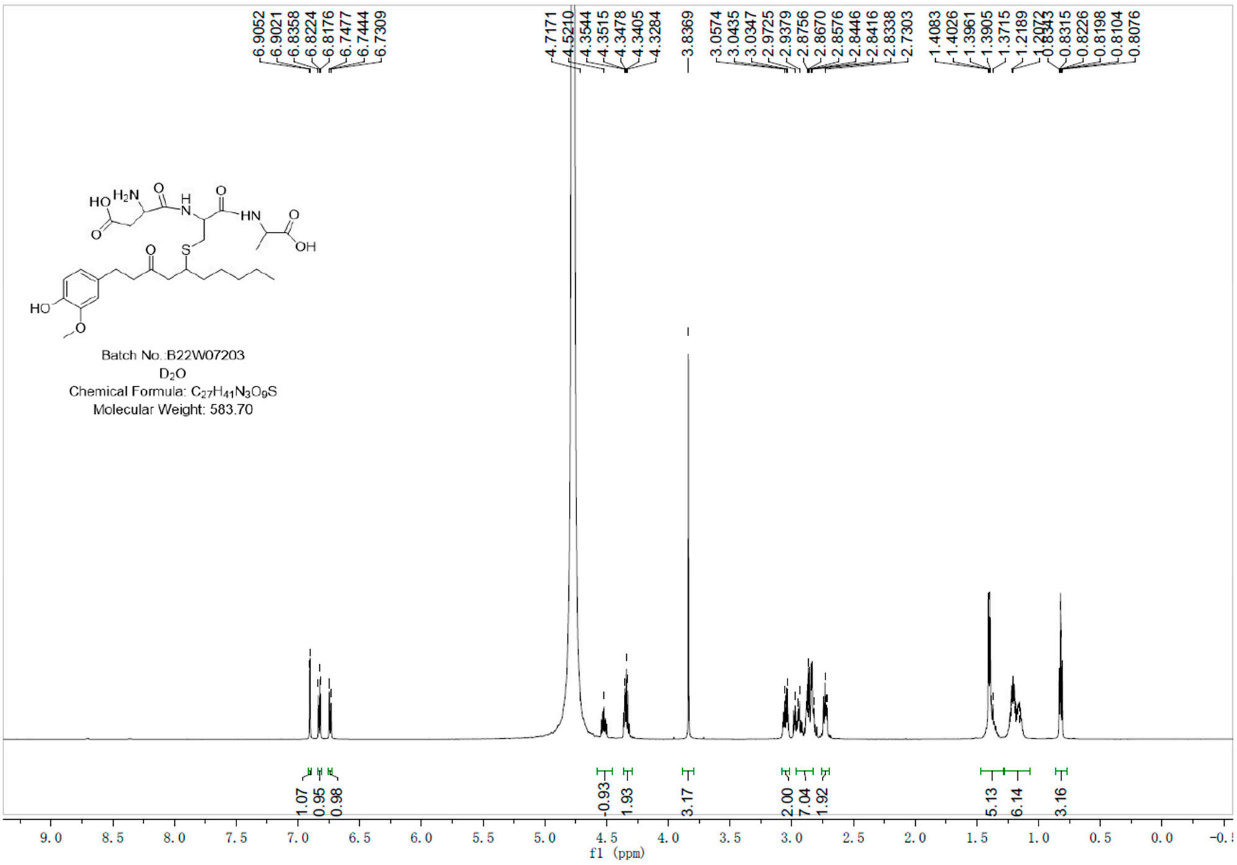

Figure S23. MLY20: (Glu(γ)-Cys(6-shogaol)-Ala). Molecular formula: C28H43N3O9S; molecular weight (MW): 597.72 Da. ESI-MS and 1H NMR spectra are shown below.

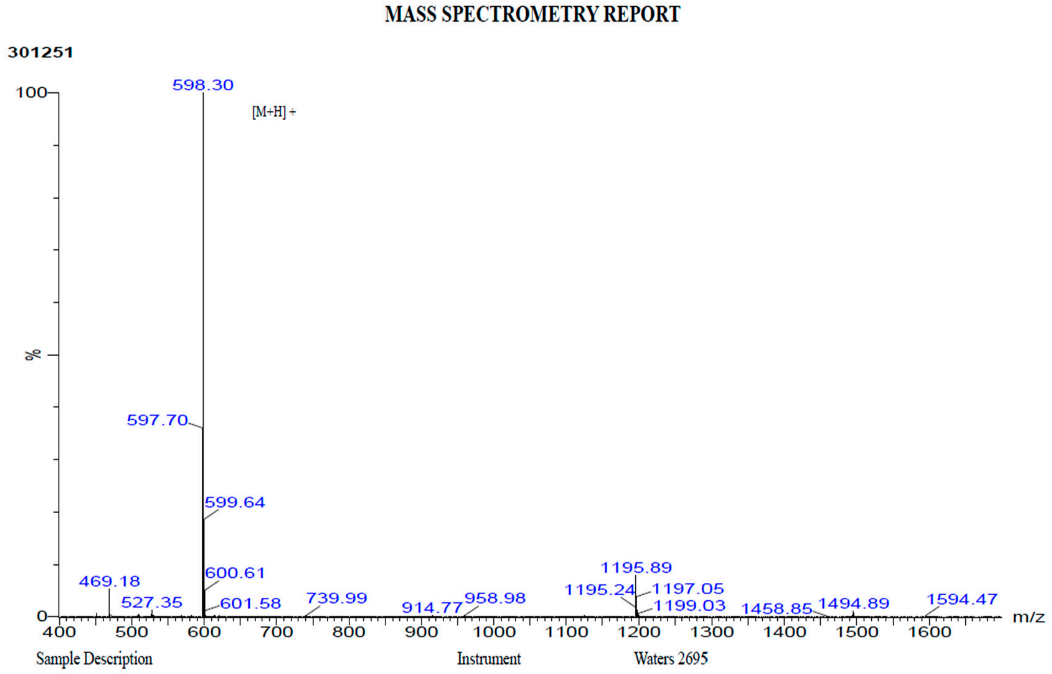

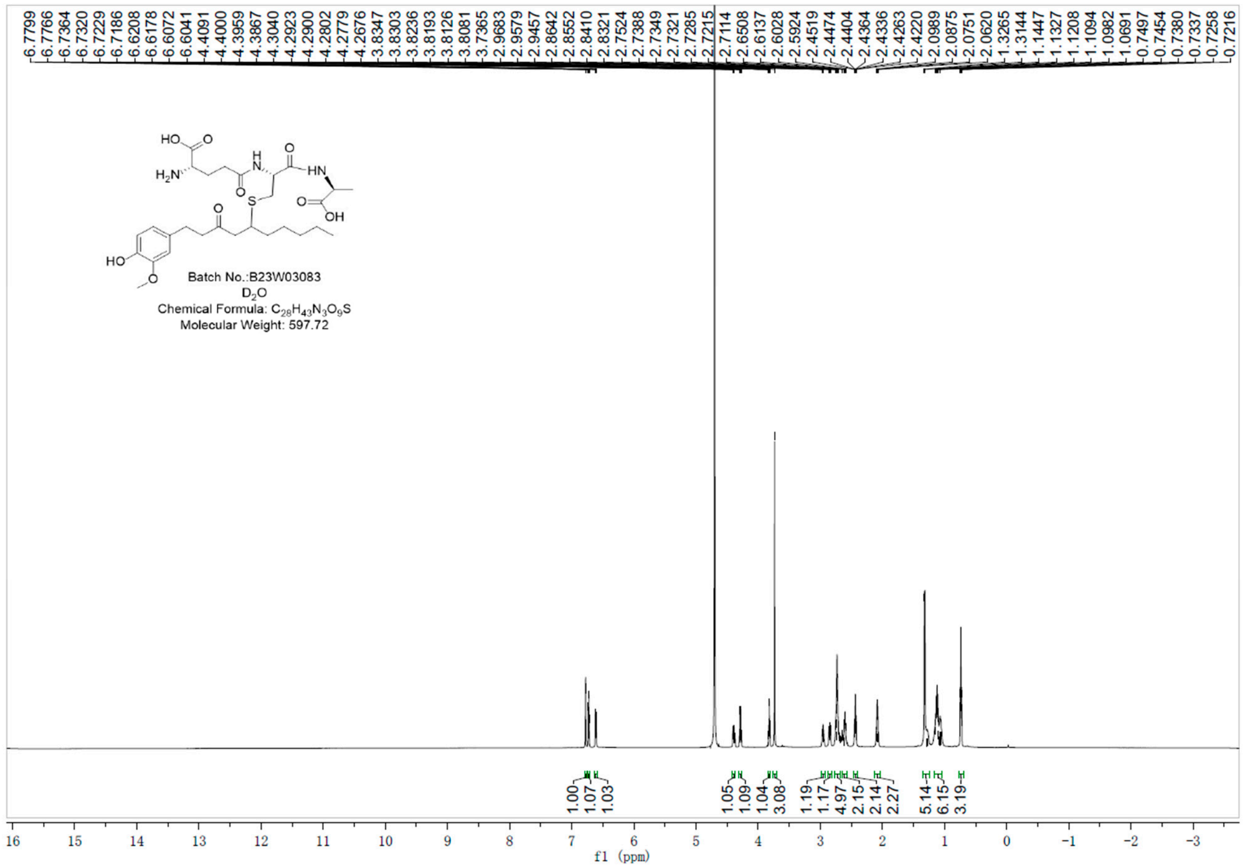

Figure S24. MLY21: (Glu(γ)-Cys(6-shogaol)-Phe). Molecular formula: C34H47N3O9S; molecular weight (MW): 673.82 Da. ESI-MS and 1H NMR spectra are shown below.

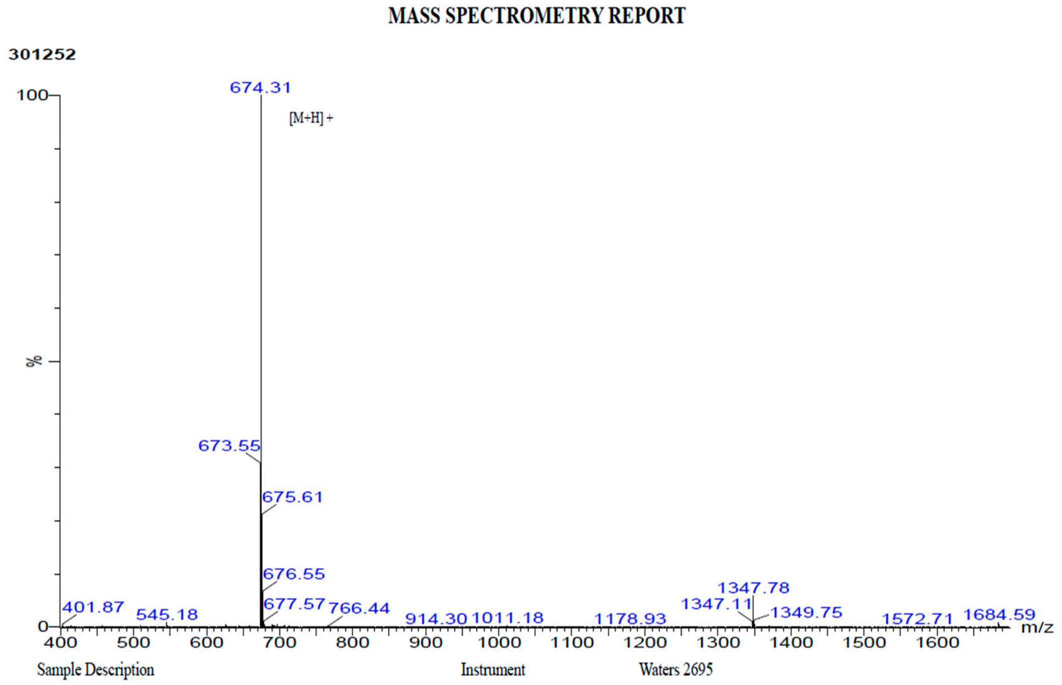

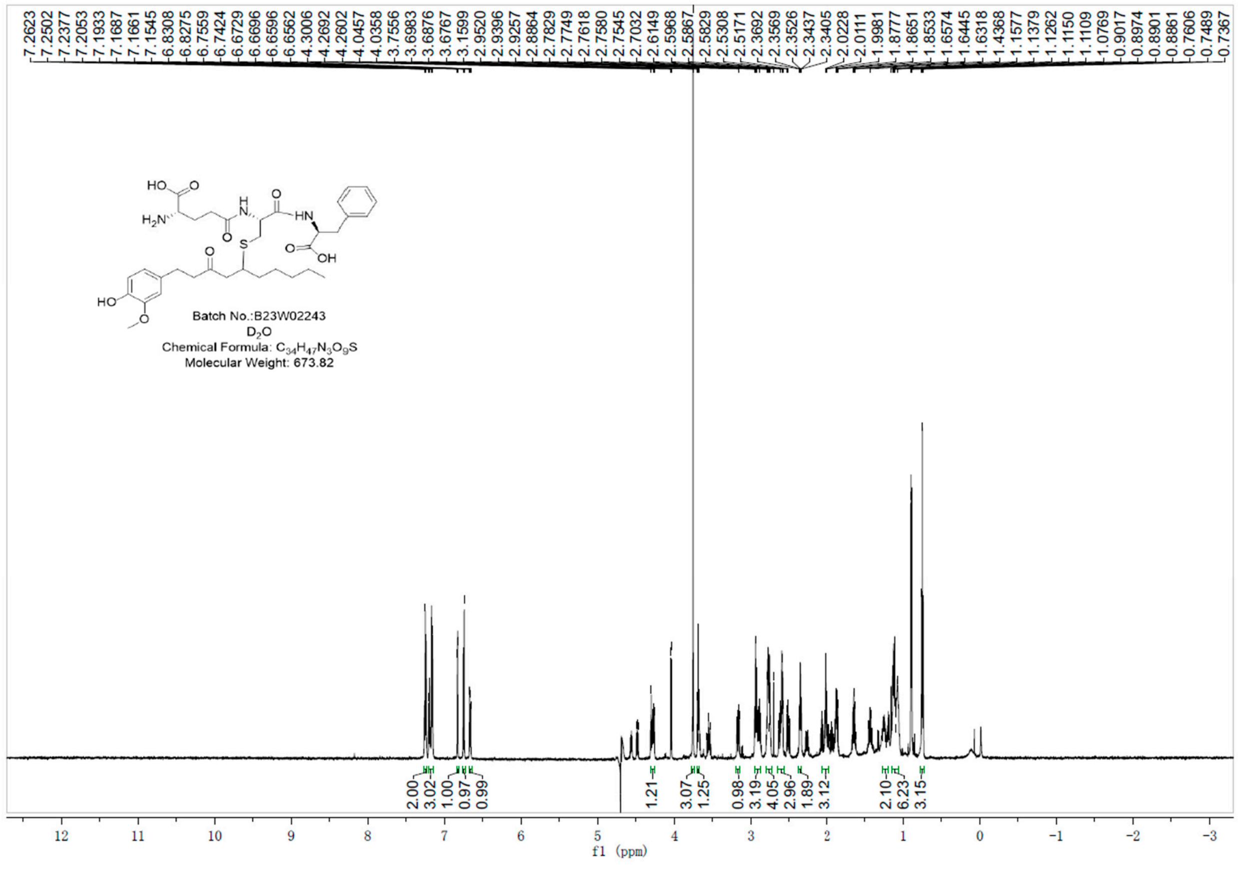

Figure S25. MLY22: (Glu(γ)-Cys(6-shogaol)-Ile). Molecular formula: $C_{31}H_{49}N_3O_9S$; molecular weight (MW): 639.81 Da. ESI-MS and 1H NMR spectra are shown below.

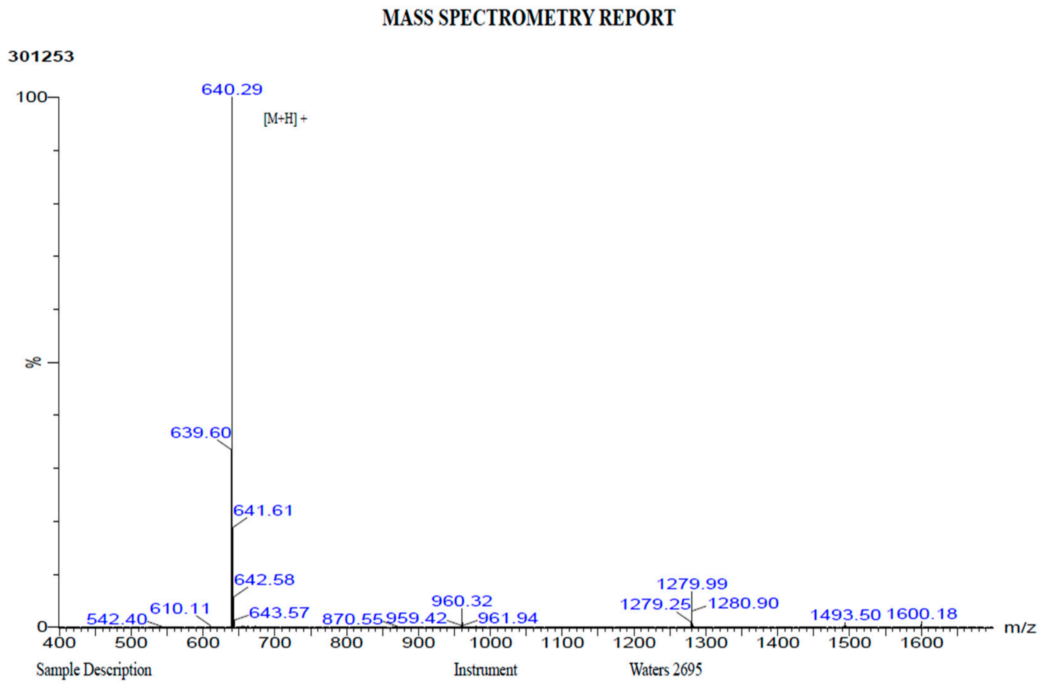

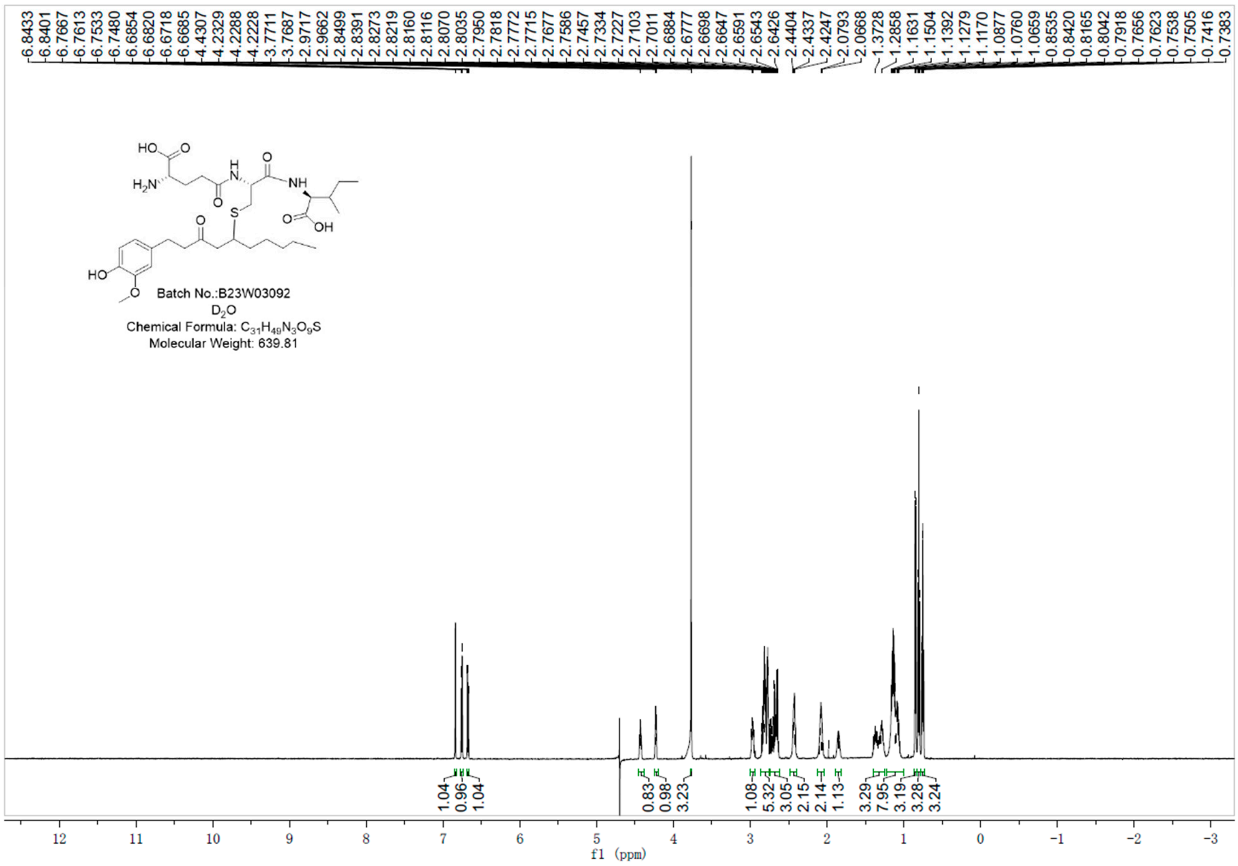

Figure S26. MLY23: (Glu(γ)-Cys(6-shogaol)-Leu). Molecular formula: C31H49N3O9S; molecular weight (MW): 639.81 Da. ESI-MS and 1H NMR spectra are shown below.

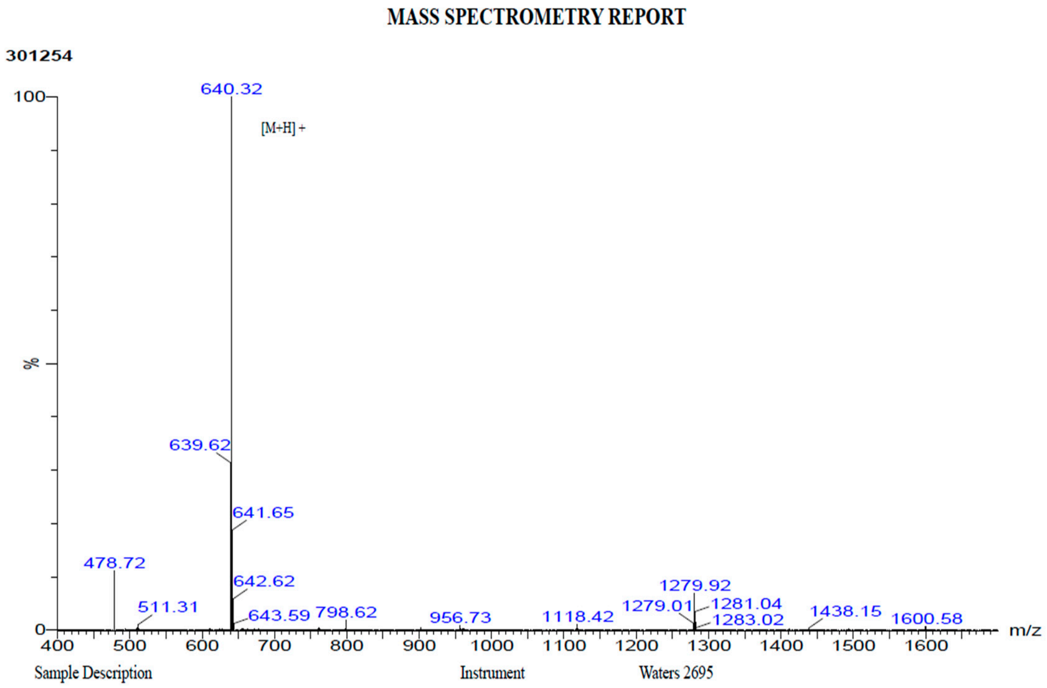

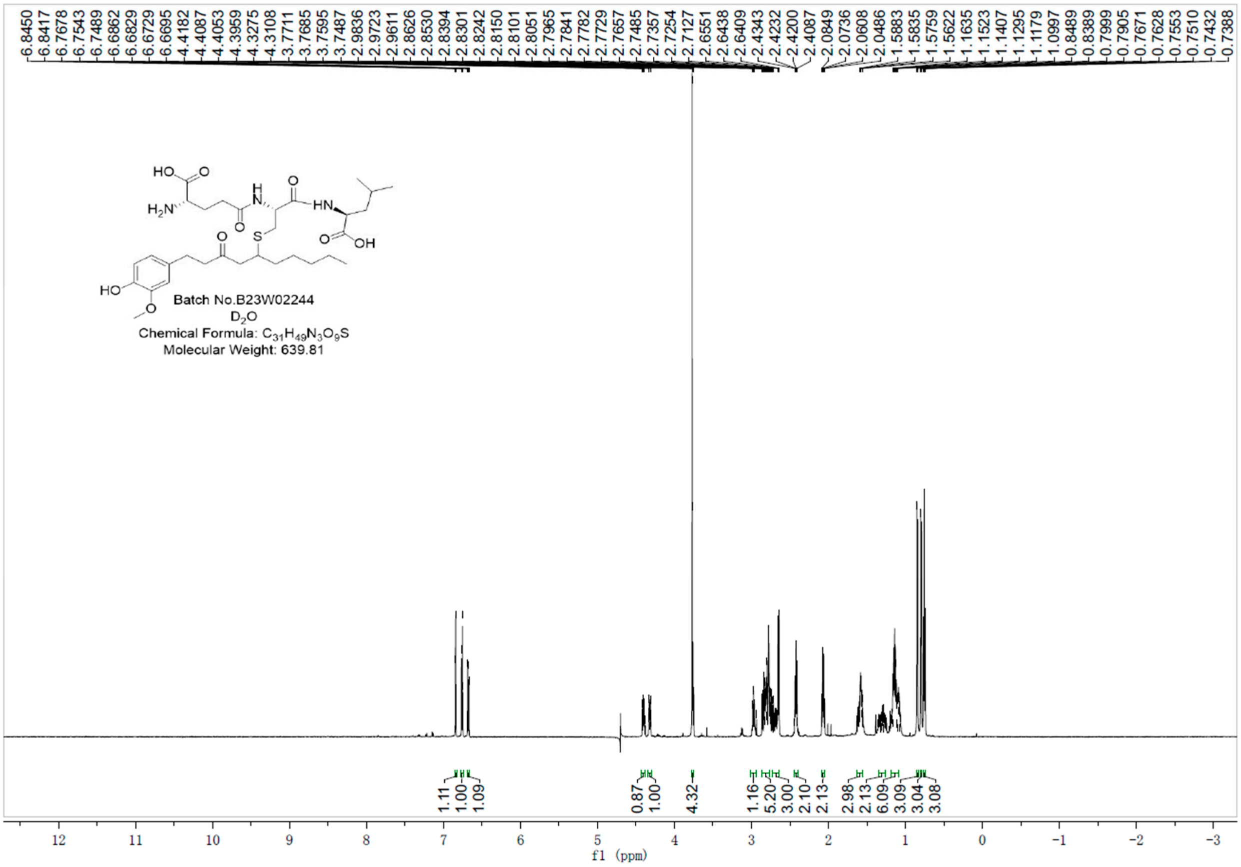

Figure S27. MLY24: (Glu(γ)-Cys(6-shogaol)-Met). Molecular formula: $C_{30}H_{47}N_3O_9S_2$; molecular weight (MW): 657.84 Da. ESI-MS and 1H NMR spectra are shown below.

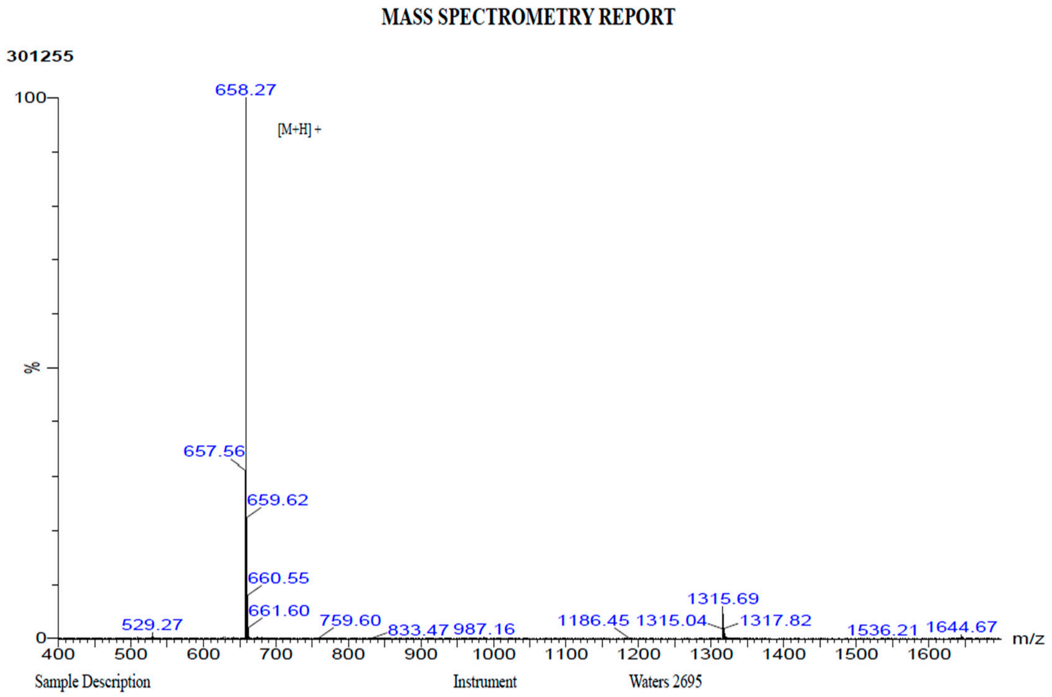

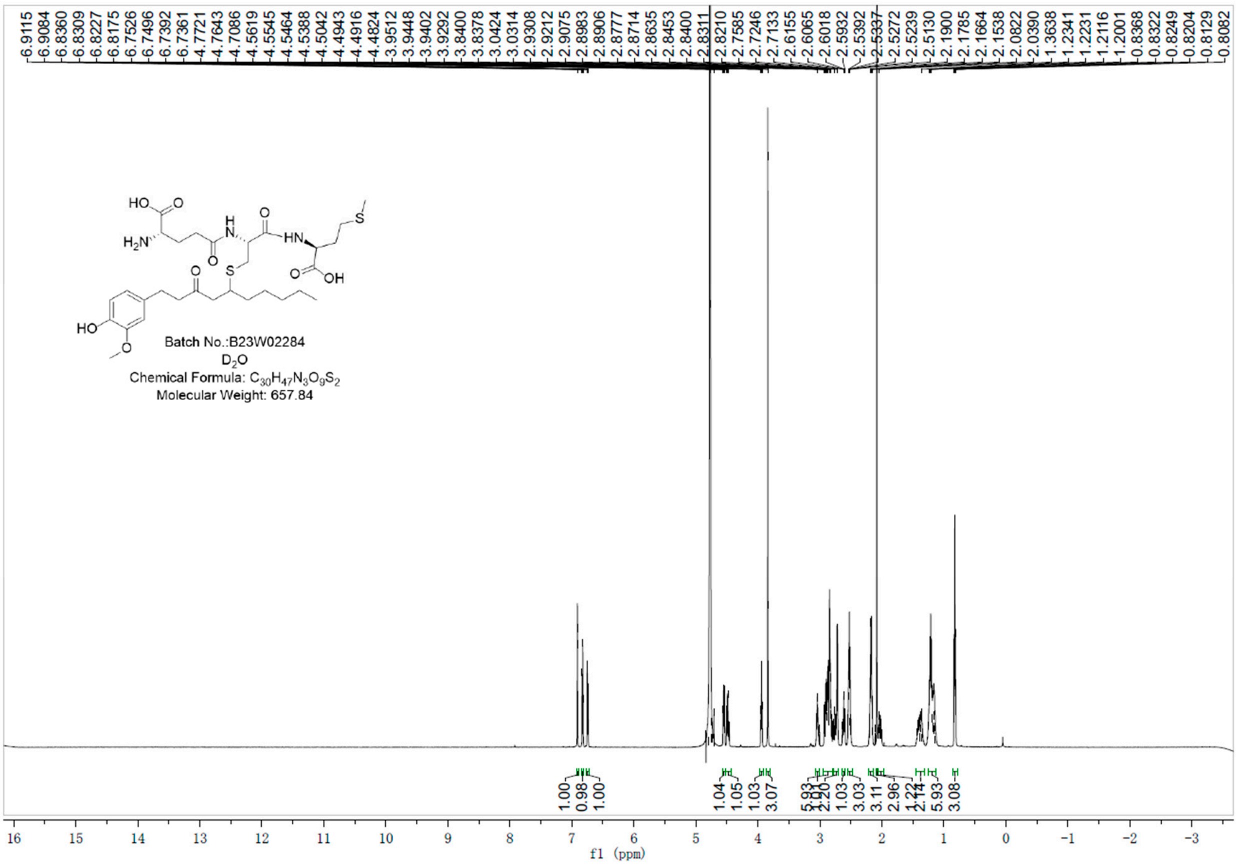

Figure S28. MLY25: (Glu(γ)-Cys(6-shogaol)-Pro). Molecular formula: $C_{30}H_{45}N_3O_9S$; molecular weight (MW): 623.76 Da. ESI-MS and 1H NMR spectra are shown below.

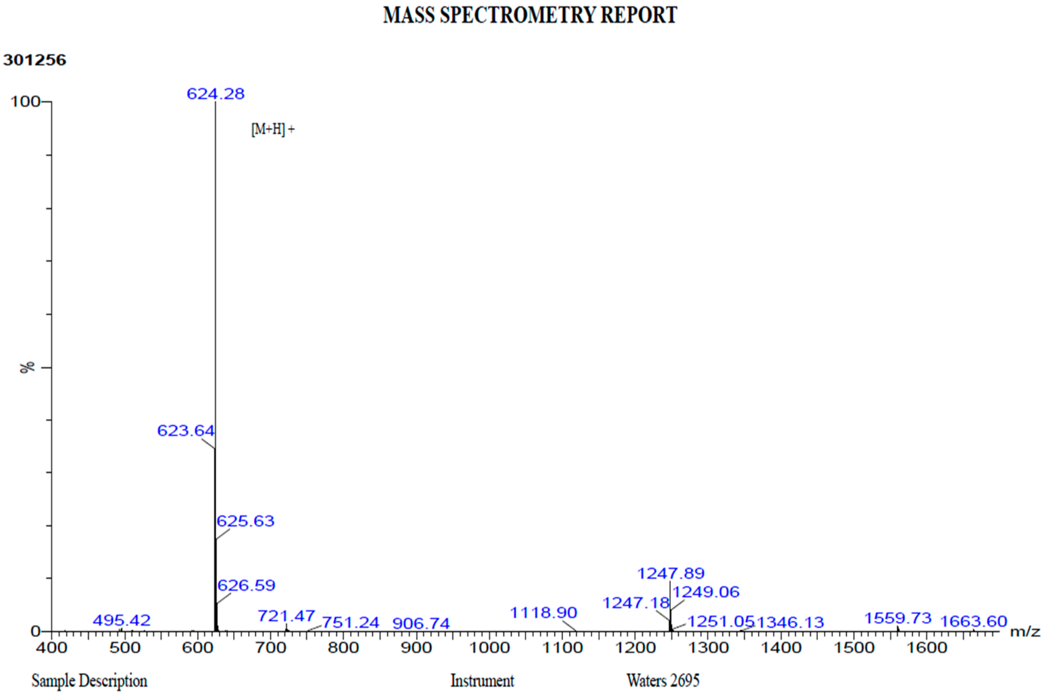

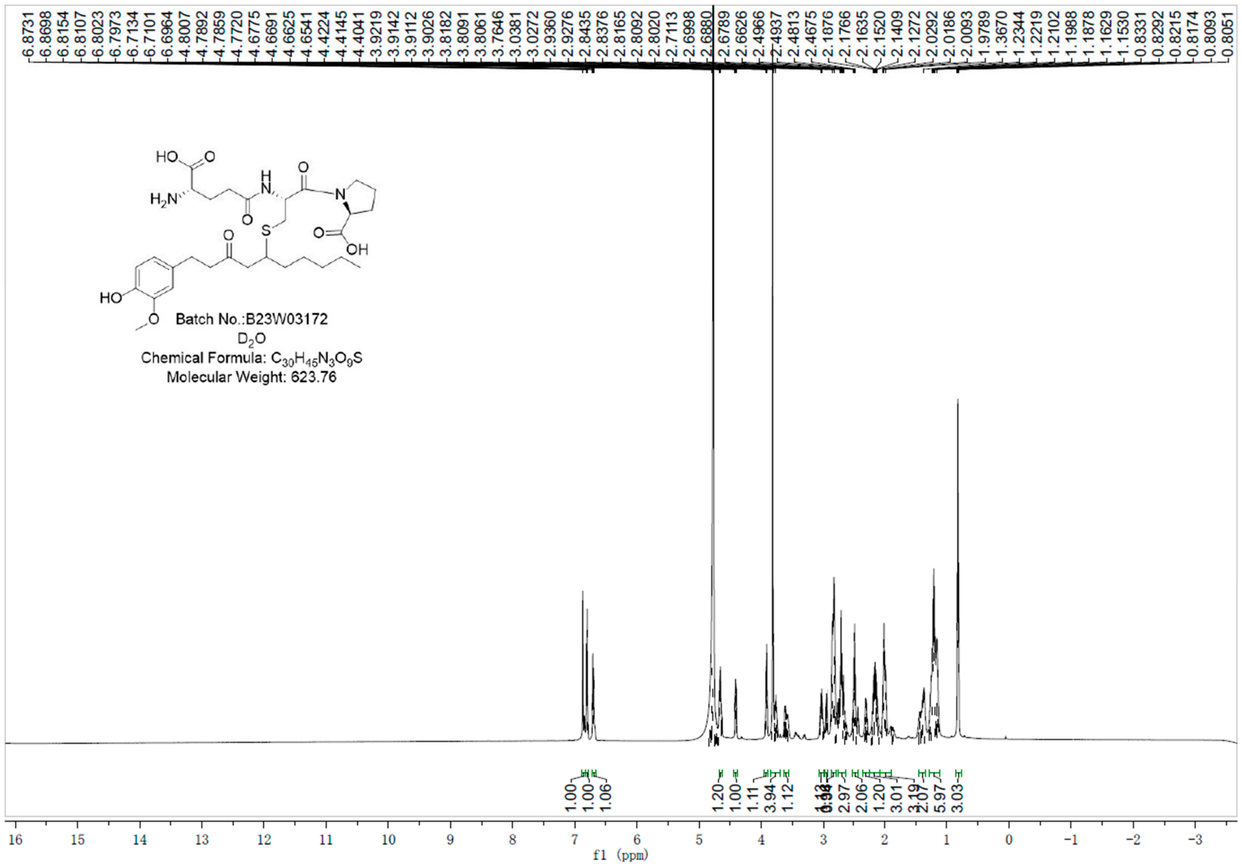

Figure S29. MLY26: (Glu(γ)-Cys(6-shogaol)-Val). Molecular formula: $C_{30}H_{47}N_3O_9S$; molecular weight (MW): 625.78 Da. ESI-MS and 1H NMR spectra are shown below.

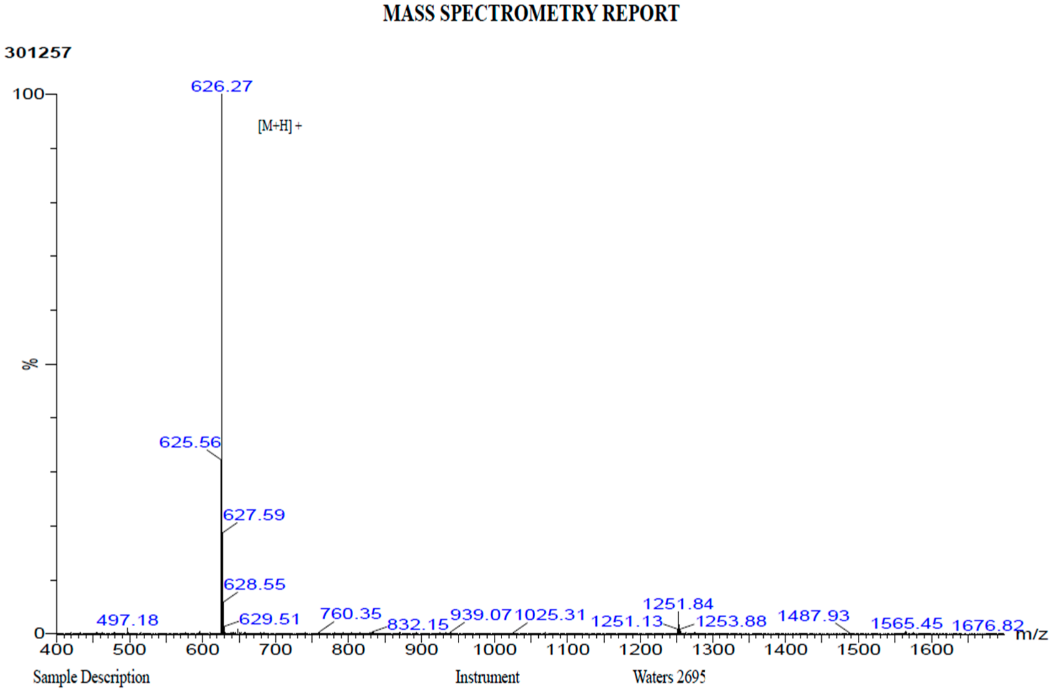

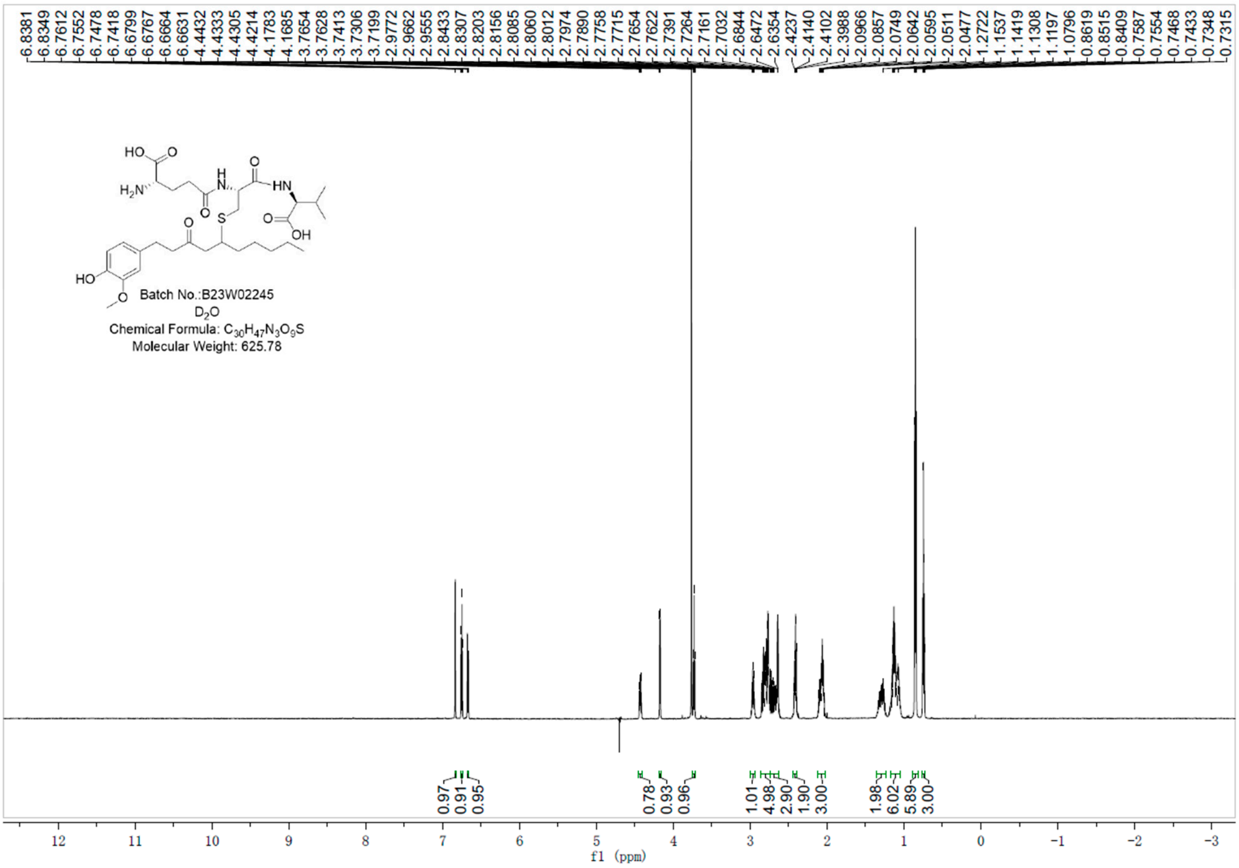

Figure S30. MLY27: (Glu(γ)-Cys(6-shogaol)-Trp). Molecular formula: $C_{36}H_{48}N_4O_9S$; molecular weight (MW): 712.86 Da. ESI-MS and 1H NMR spectra are shown below.

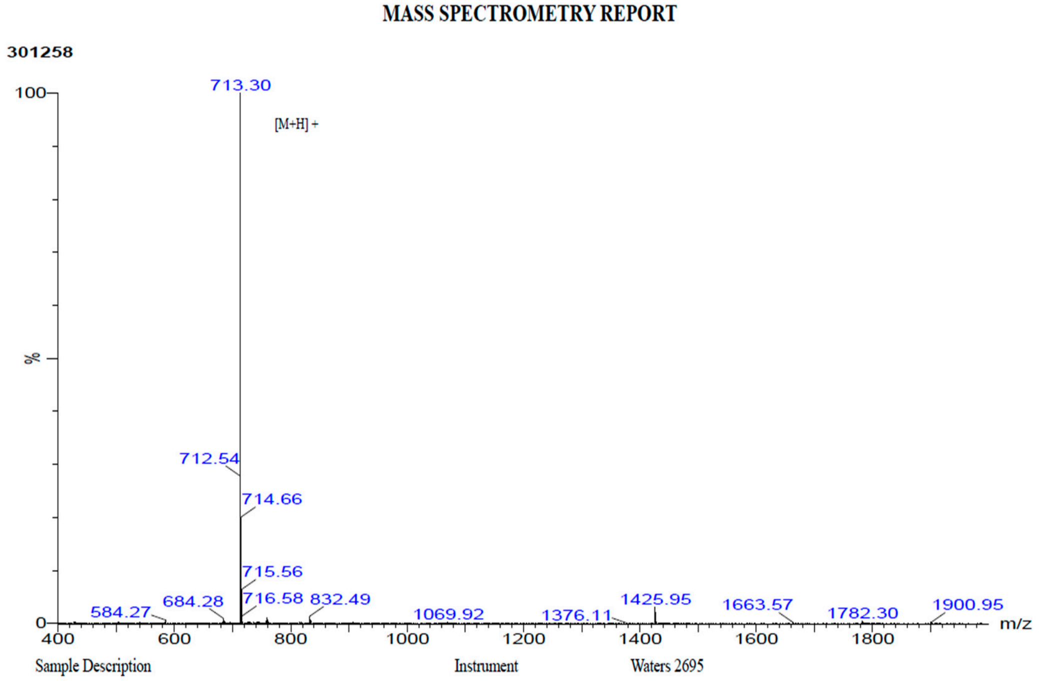

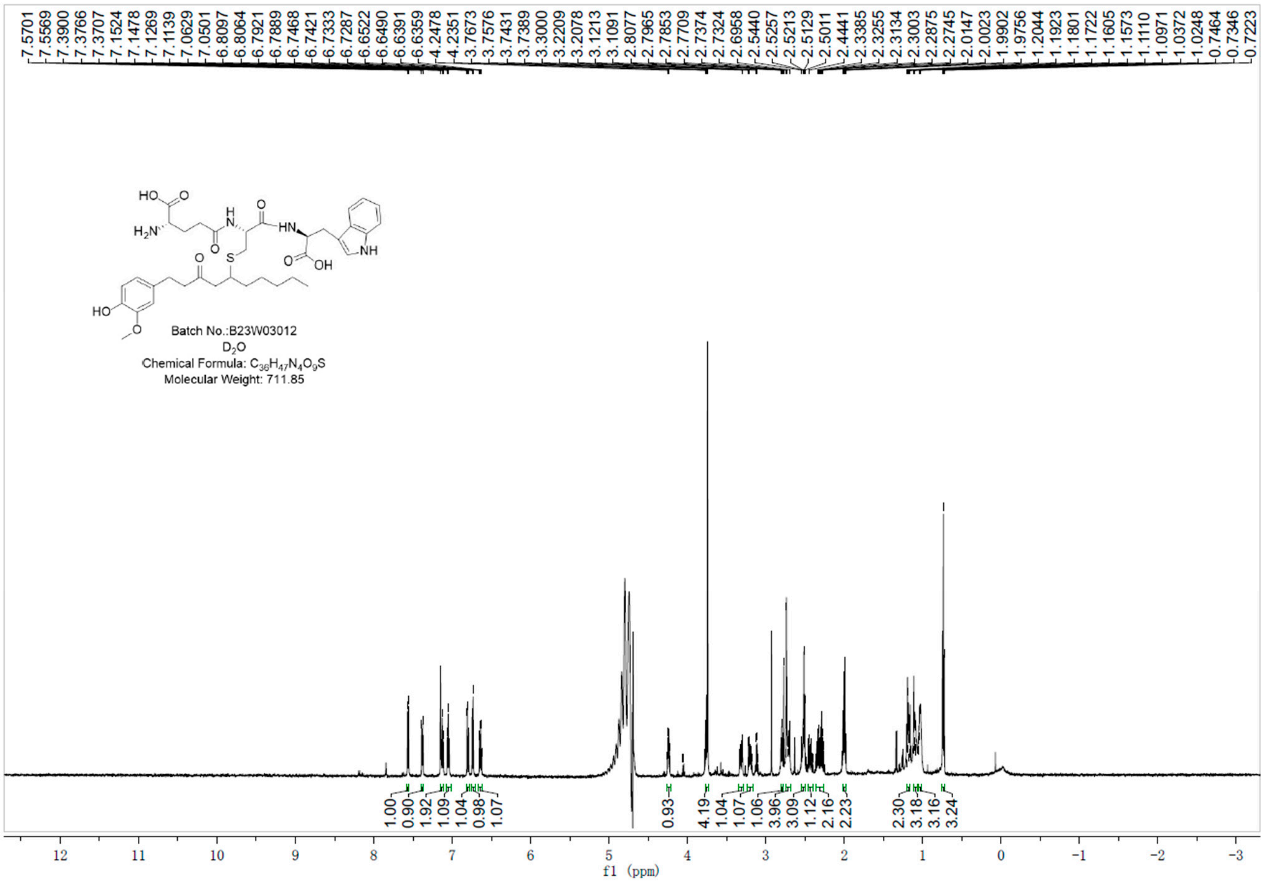

Figure S31. MLY28: (Glu(γ)-Cys(6-shogaol)-Tyr). Molecular formula: $C_{34}H_{47}N_3O_{10}S$; molecular weight (MW): 689.82 Da. ESI-MS and 1H NMR spectra are shown below.

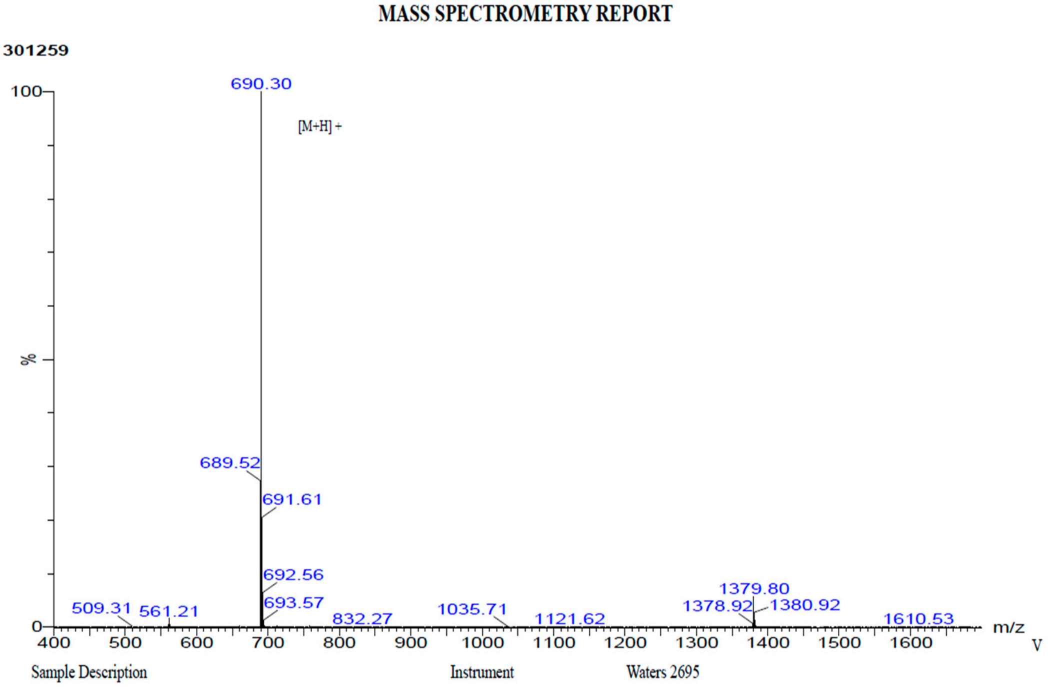

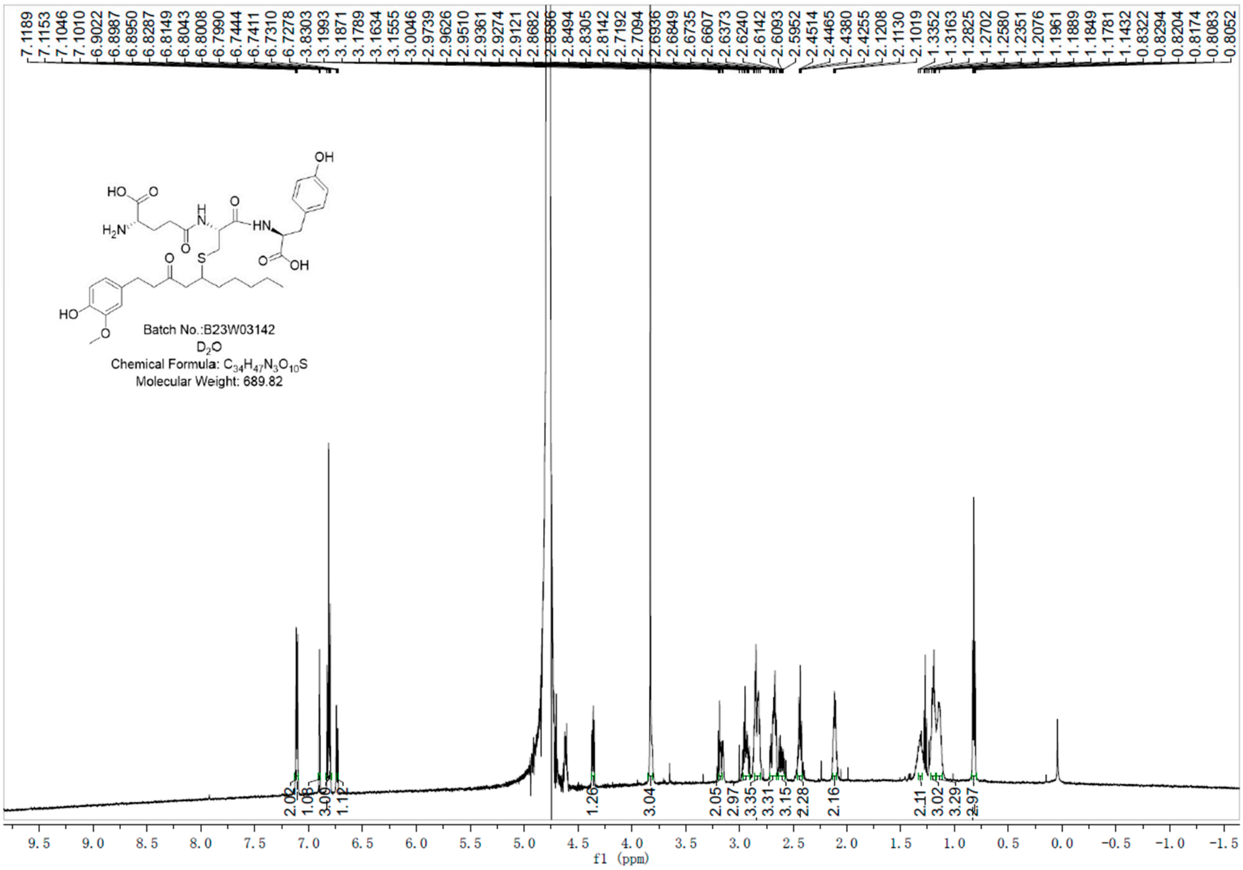

Figure S32. MLY29: (Asp(β)-Cys(6-shogaol)-Gly). Molecular formula: $C_{26}H_{39}N_3O_9S$; molecular weight (MW): 569.67 Da. ESI-MS and 1H NMR spectra are shown below.

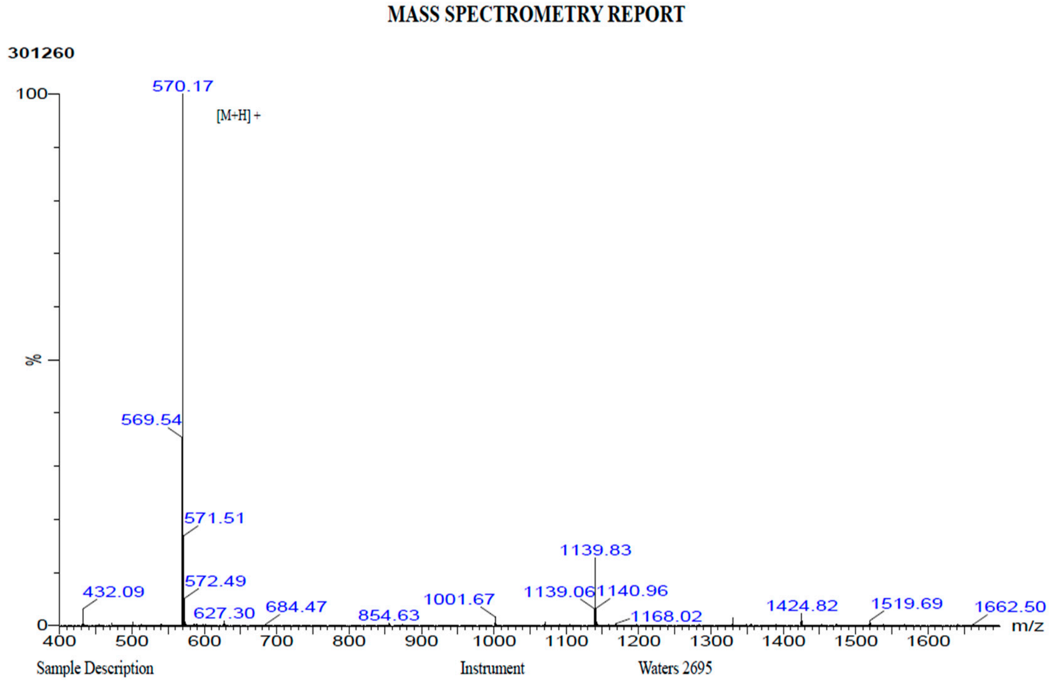

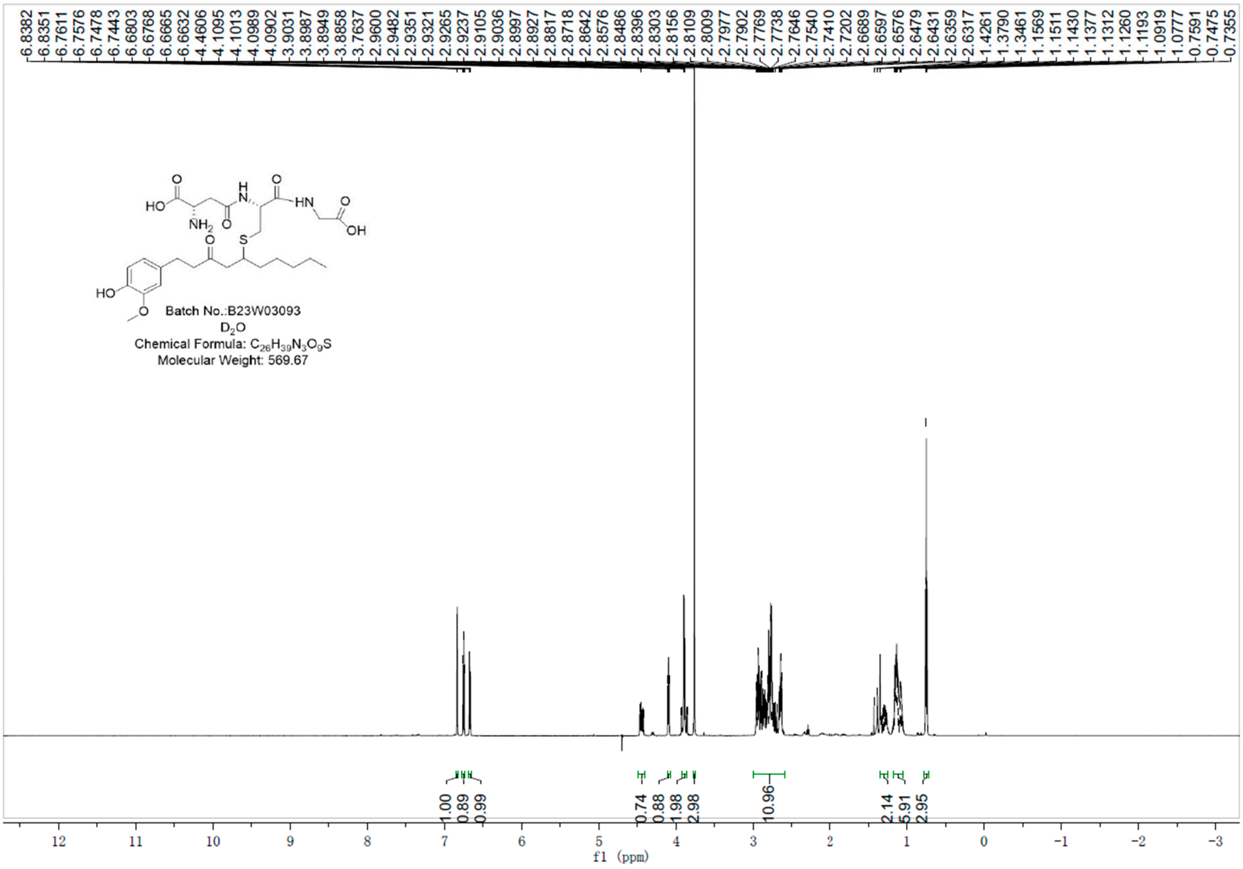

Figure S33. MLY30: (Asp(β)-Cys(6-shogaol)-Ala). Molecular formula: $C_{27}H_{41}N_3O_9S$; molecular weight (MW): 583.70 Da. ESI-MS and 1H NMR spectra are shown below.

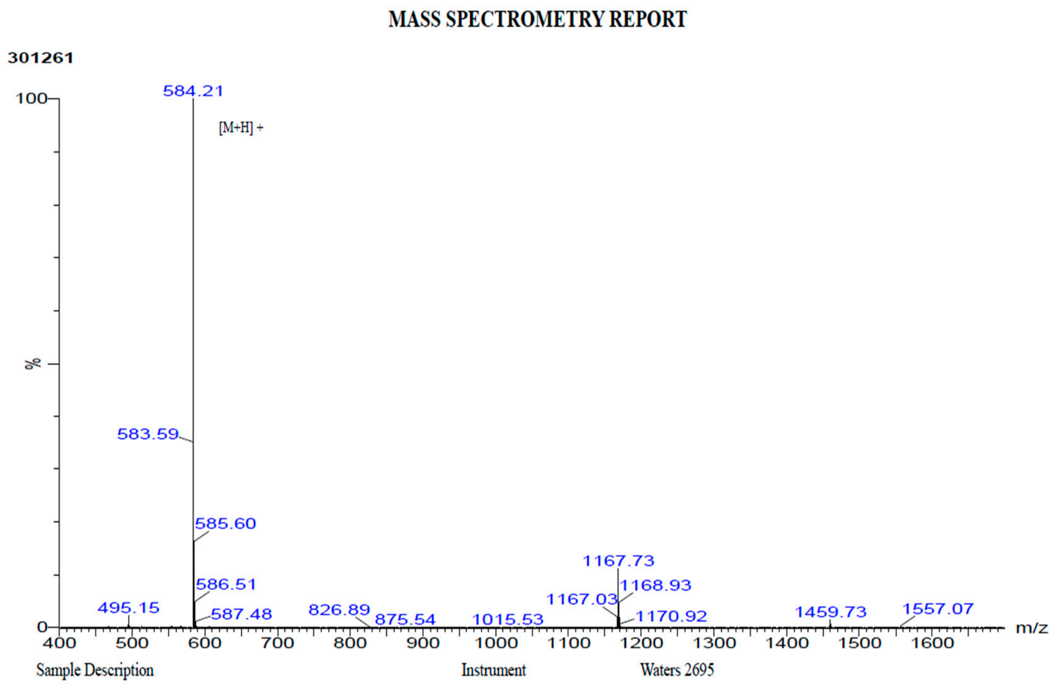

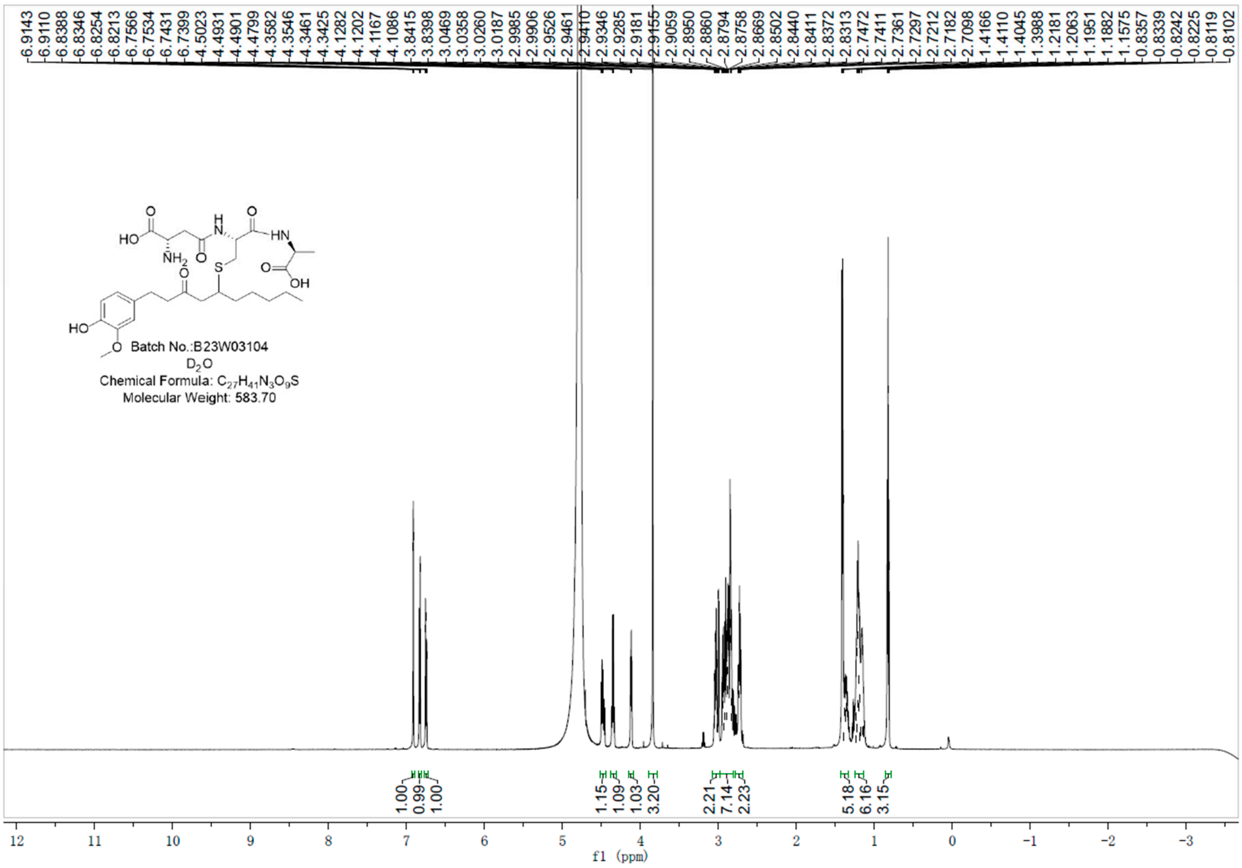

Figure S34. MLY31: (Asp(β)-Cys(6-shogaol)-Phe). Molecular formula: $C_{33}H_{45}N_3O_9S$; molecular weight (MW): 659.80 Da. ESI-MS and 1H NMR spectra are shown below.

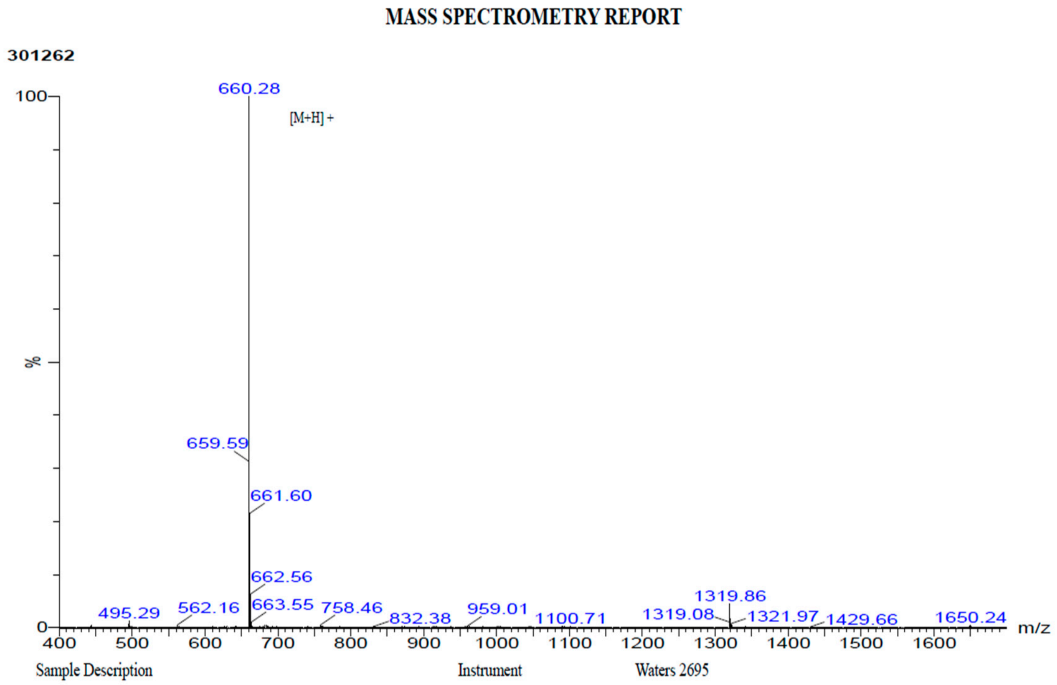

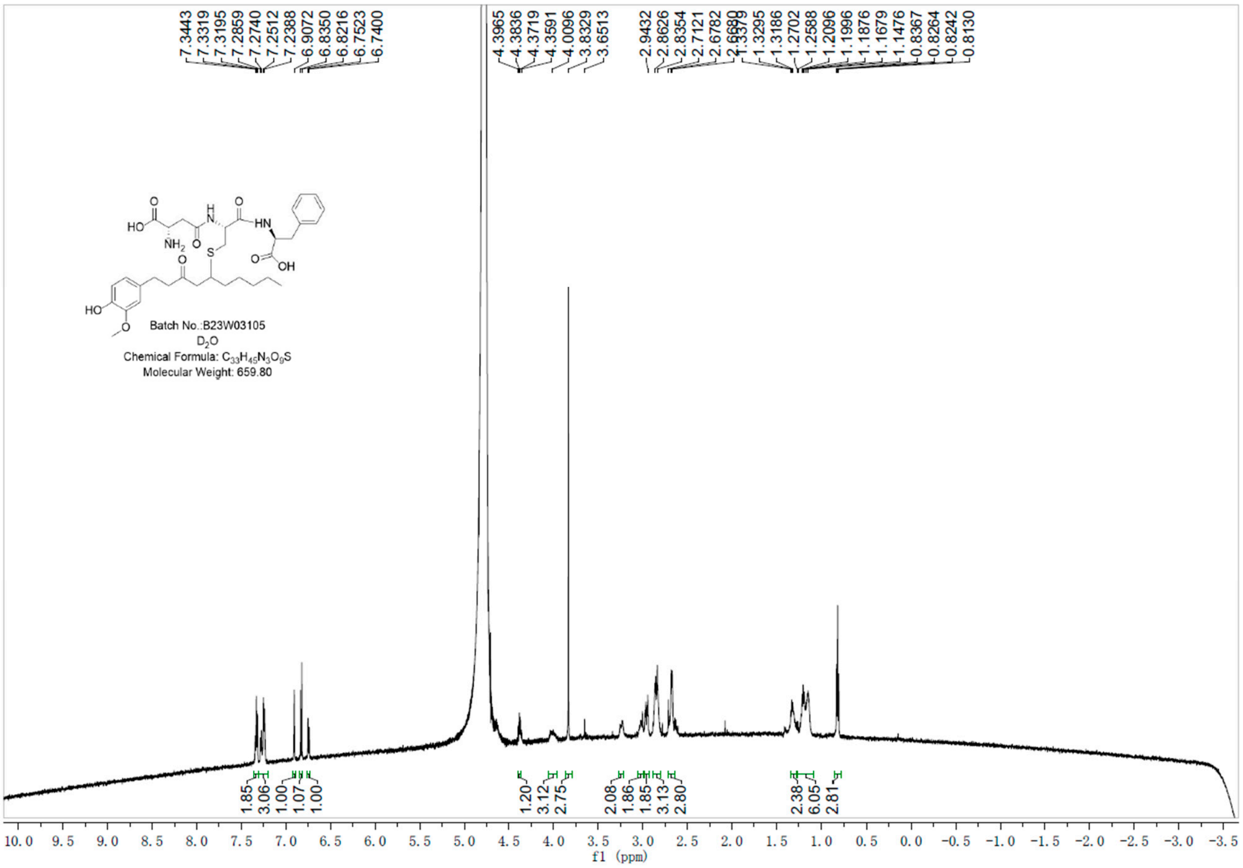

Figure S35. MLY32: (Asp(β)-Cys(6-shogaol)-Ile). Molecular formula: $C_{30}H_{47}N_3O_9S$; molecular weight (MW): 625.78 Da. ESI-MS and 1H NMR spectra are shown below.

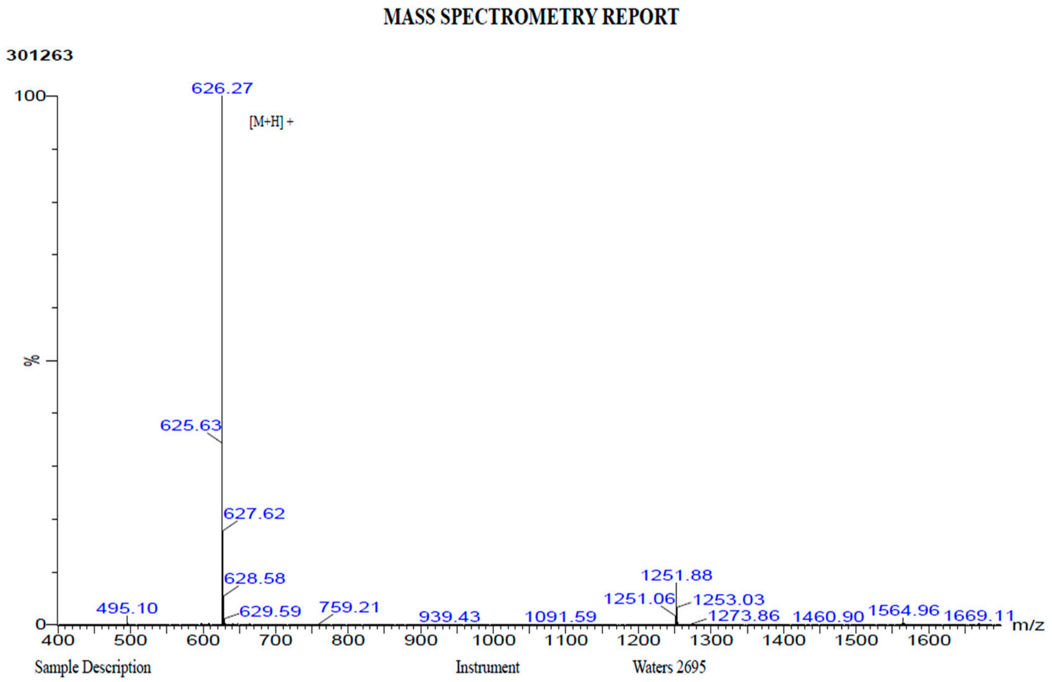

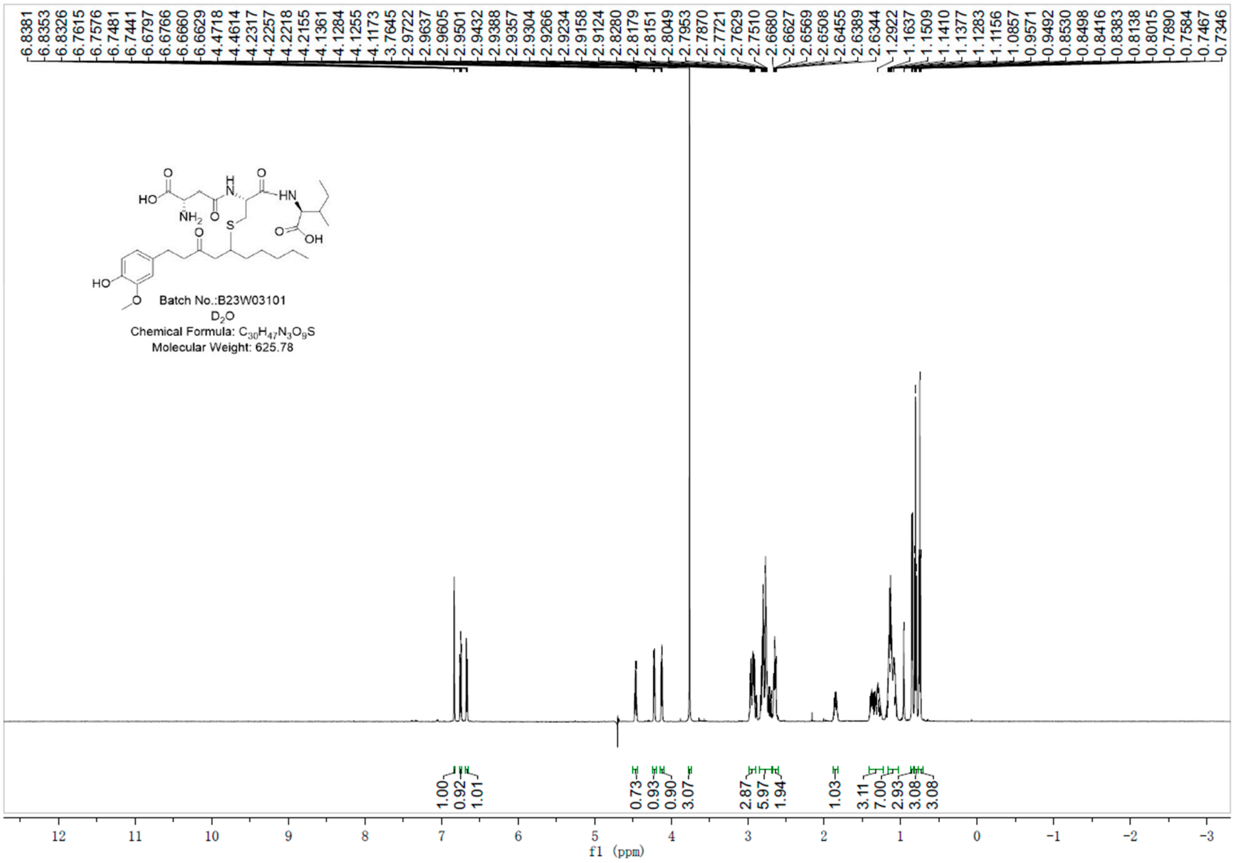

Figure S36. MLY33: (Asp(β)-Cys(6-shogaol)-Leu). Molecular formula: $C_{30}H_{47}N_3O_9S$; molecular weight (MW): 625.78 Da. ESI-MS and 1H NMR spectra are shown below.

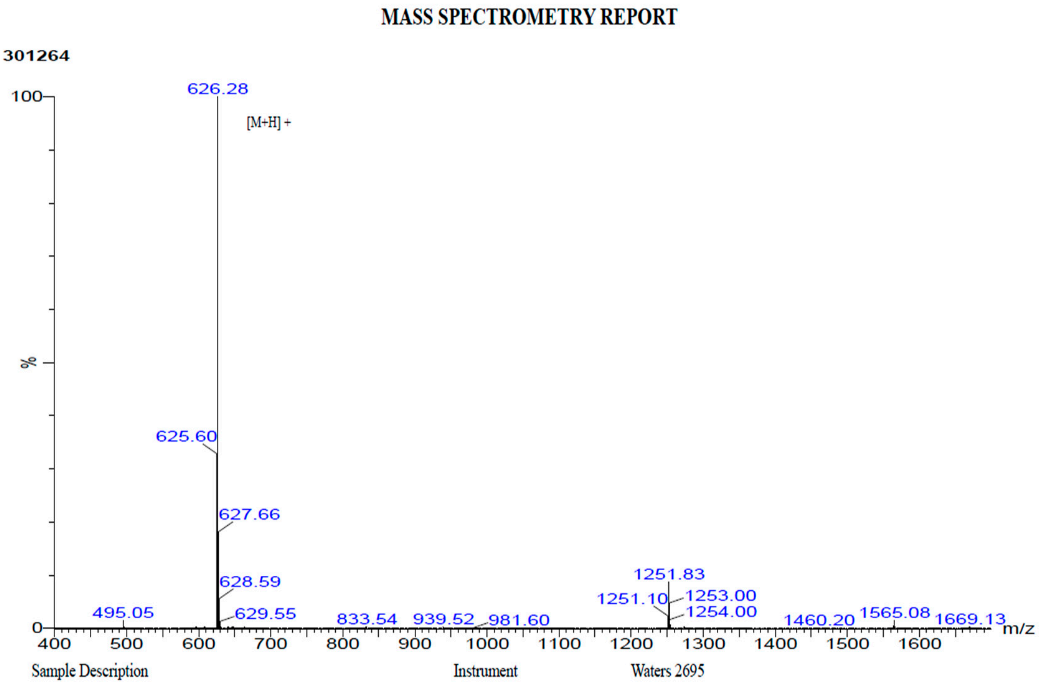

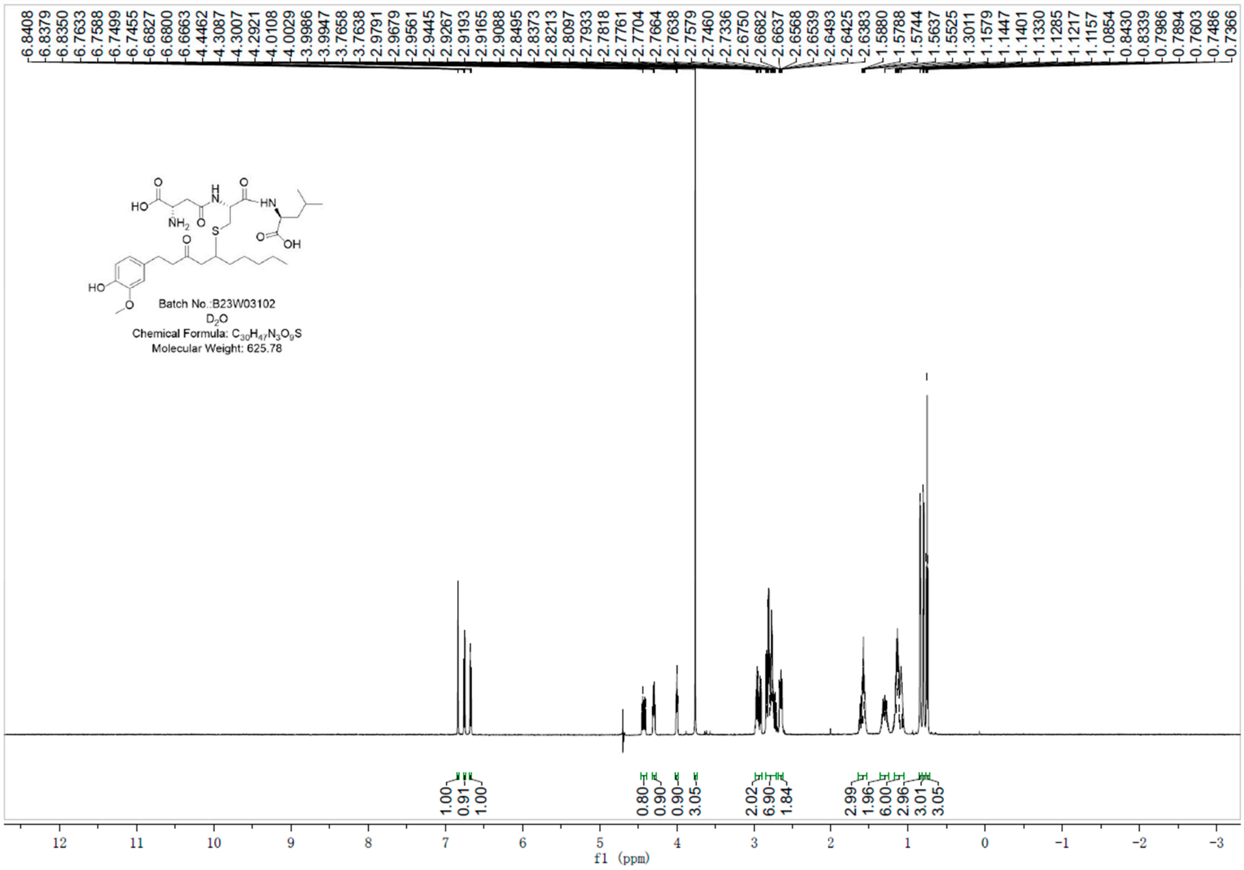

Figure S37. MLY34: (Asp(β)-Cys(6-shogaol)-Met). Molecular formula: $C_{29}H_{45}N_3O_9S_2$; molecular weight (MW): 643.81 Da. ESI-MS and 1H NMR spectra are shown below.

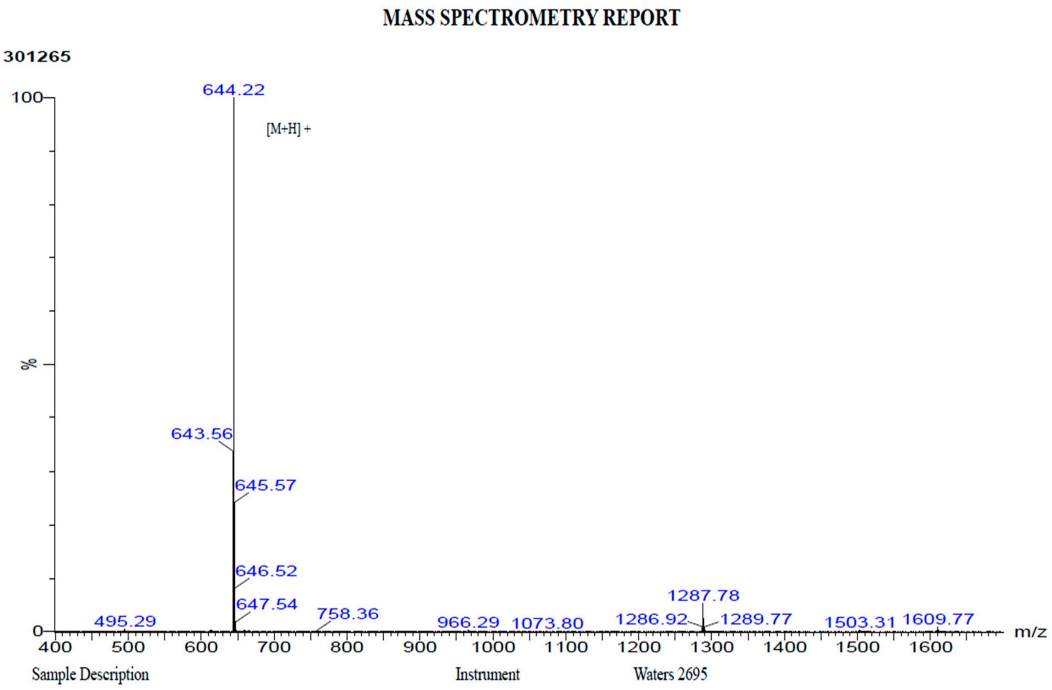

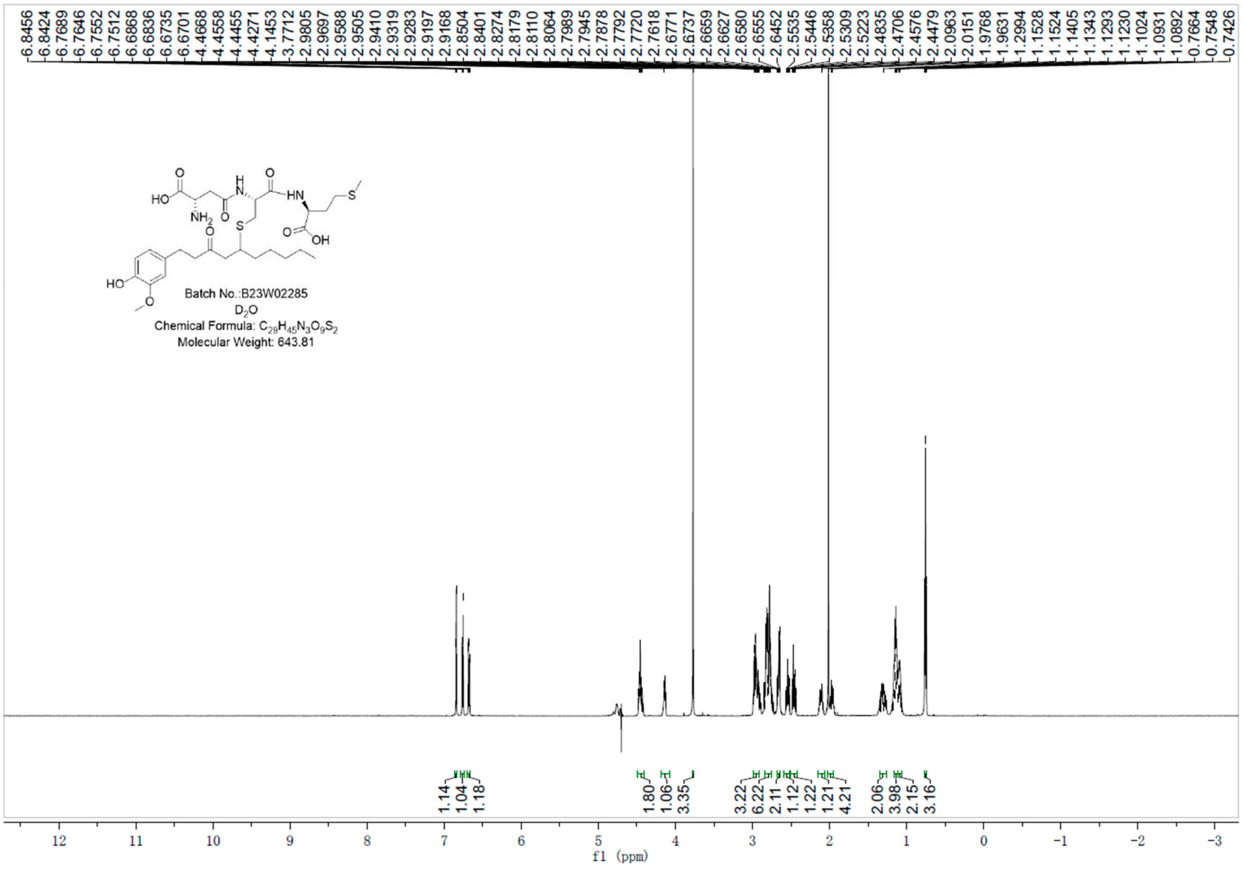

Figure S38. MLY35: (Asp(β)-Cys(6-shogaol)-Pro). Molecular formula: $C_{29}H_{43}N_3O_9S$; molecular weight (MW): 609.74 Da. ESI-MS and 1H NMR spectra are shown below.

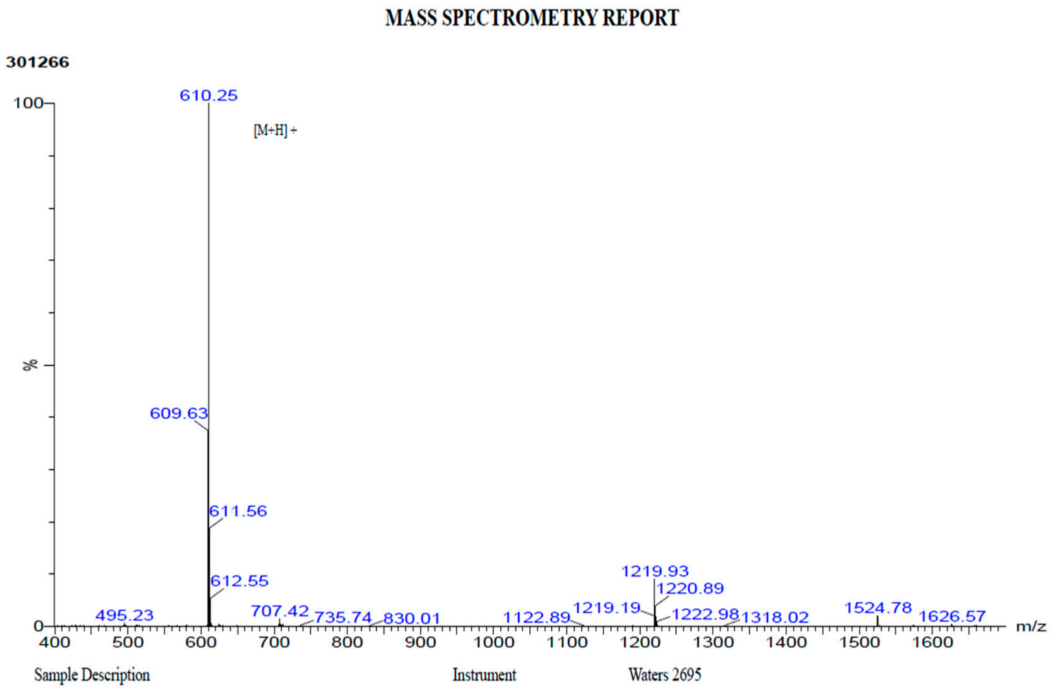

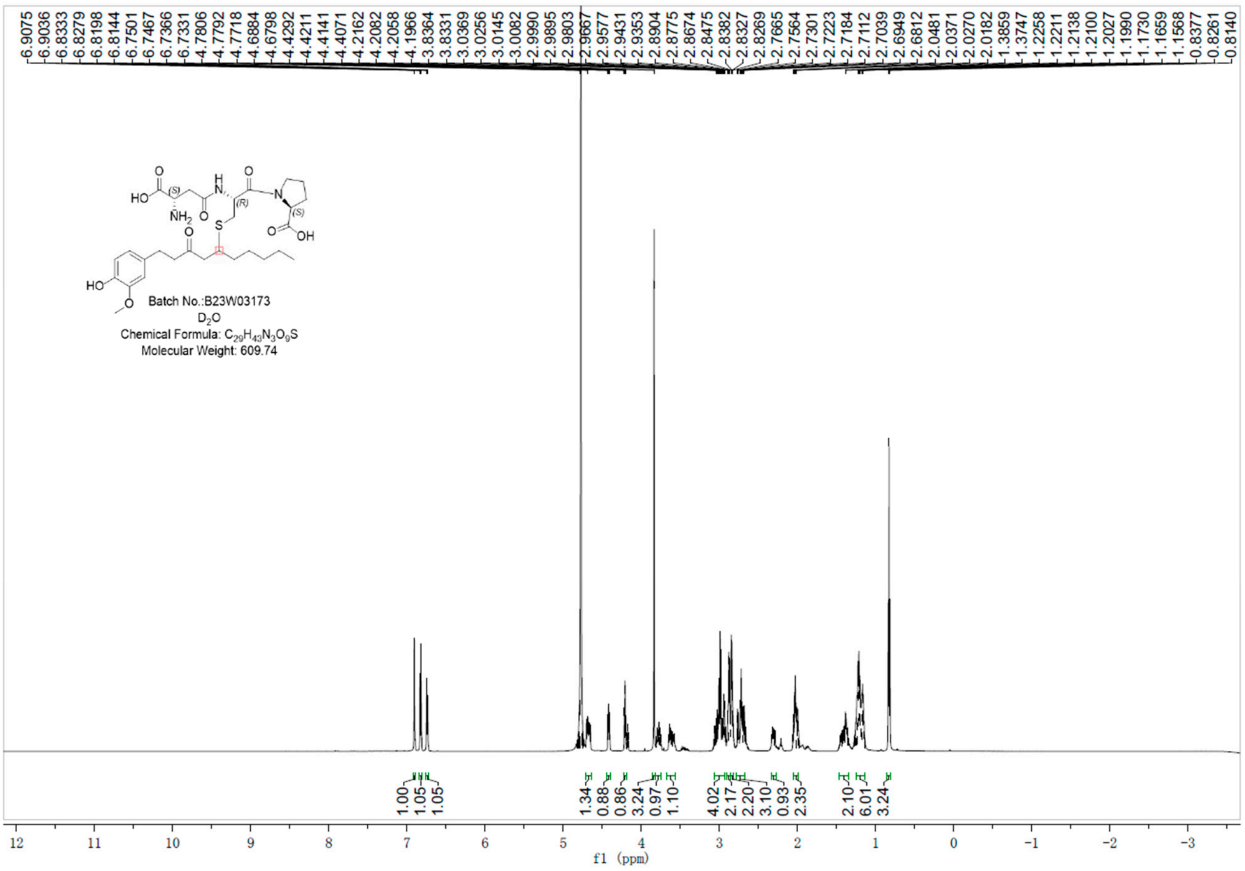

Figure S39. MLY36: (Asp(β)-Cys(6-shogaol)-Val). Molecular formula: $C_{29}H_{45}N_3O_9S$; molecular weight (MW): 611.75 Da. ESI-MS and 1H NMR spectra are shown below.

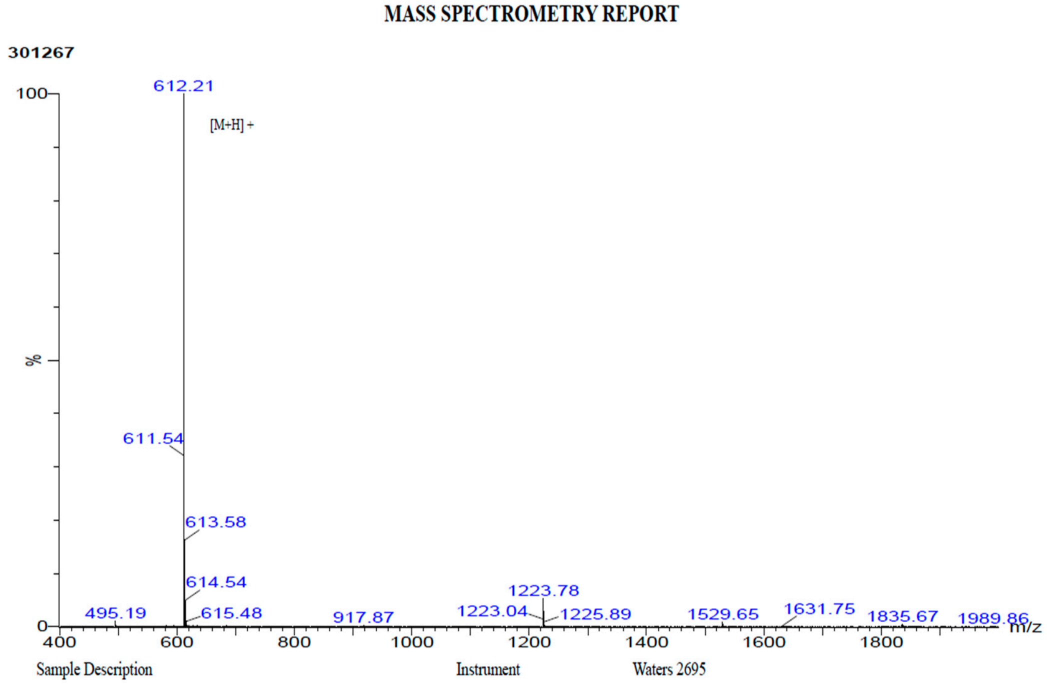

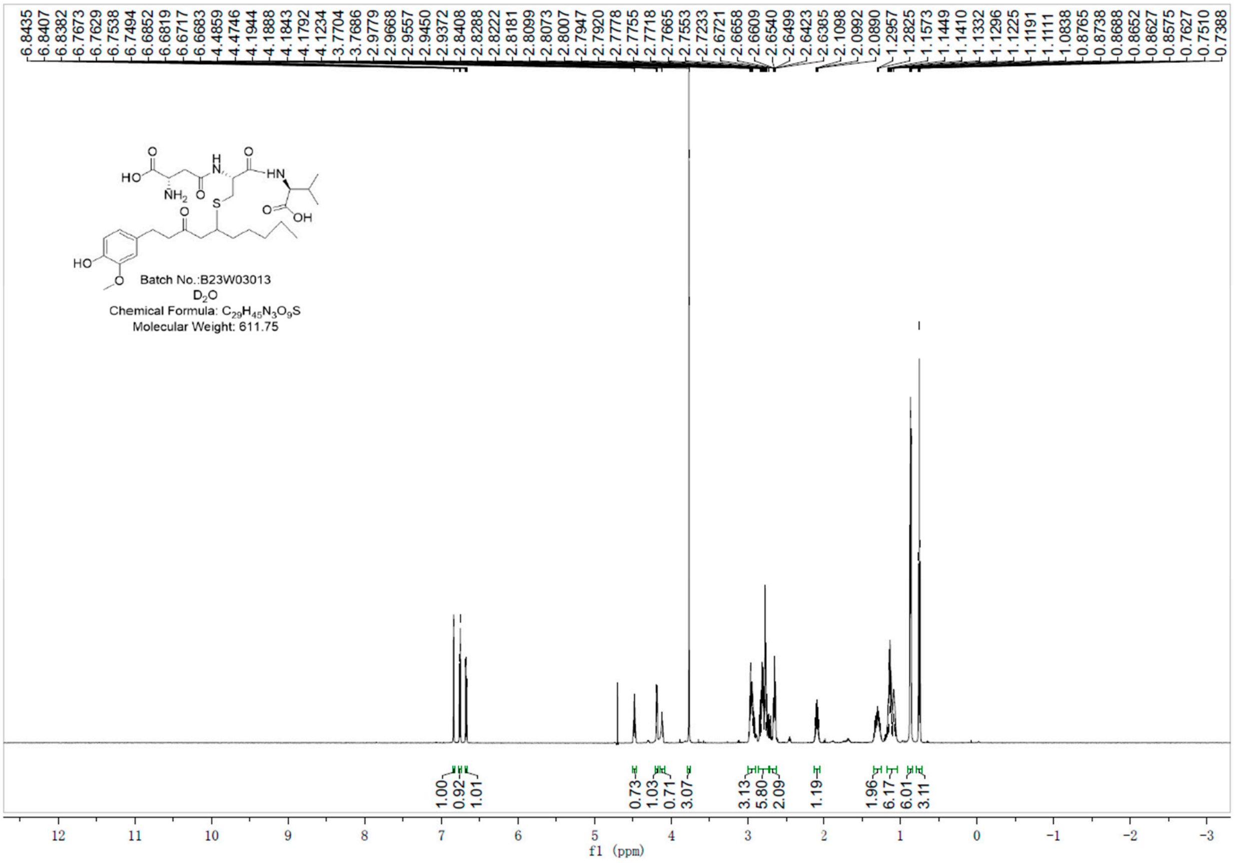

Supplement: Supplementary file 1 [file ijms-27-08285-s001.zip › ijms-4518521-supplementary.pdf]
